# Marine Sponges as *Chloroflexi* Hot Spots: Genomic Insights and High-Resolution Visualization of an Abundant and Diverse Symbiotic Clade

**DOI:** 10.1128/mSystems.00150-18

**Published:** 2018-12-26

**Authors:** Kristina Bayer, Martin T. Jahn, Beate M. Slaby, Lucas Moitinho-Silva, Ute Hentschel

**Affiliations:** a GEOMAR-Helmholtz Centre for Ocean Research, RD3-Marine Ecology, RU-Marine Microbiology, Kiel, Germany; b University of Wuerzburg, Imaging Core Facility at the Theodor Boveri Institute of Bioscience, Wuerzburg, Germany; c Centre for Marine Bio-Innovation, University of New South Wales, Sydney, New South Wales, Australia; d Christian-Albrechts University of Kiel, Kiel, Germany; University of Tennessee at Knoxville

**Keywords:** *Chloroflexi*, DOM degradation, FISH-CLEM, metabolism, metagenomic binning, single-cell genomics, sponge symbiosis

## Abstract

Members of the widespread bacterial phylum *Chloroflexi* can dominate high-microbial-abundance (HMA) sponge microbiomes. In the Sponge Microbiome Project, *Chloroflexi* sequences amounted to 20 to 30% of the total microbiome of certain HMA sponge genera with the classes/clades SAR202, *Caldilineae*, and *Anaerolineae* being the most prominent. We performed metagenomic and single-cell genomic analyses to elucidate the functional gene repertoire of *Chloroflexi* symbionts of Aplysina aerophoba. Eighteen draft genomes were reconstructed and placed into phylogenetic context of which six were investigated in detail. Common genomic features of *Chloroflexi* sponge symbionts were related to central energy and carbon converting pathways, amino acid and fatty acid metabolism, and respiration. Clade-specific metabolic features included a massively expanded genomic repertoire for carbohydrate degradation in *Anaerolineae* and *Caldilineae* genomes, but only amino acid utilization by SAR202. While *Anaerolineae* and *Caldilineae* import cofactors and vitamins, SAR202 genomes harbor genes encoding components involved in cofactor biosynthesis. A number of features relevant to symbiosis were further identified, including CRISPR-Cas systems, eukaryote-like repeat proteins, and secondary metabolite gene clusters. *Chloroflexi* symbionts were visualized in the sponge extracellular matrix at ultrastructural resolution by the fluorescence *in situ* hybridization-correlative light and electron microscopy (FISH-CLEM) method. Carbohydrate degradation potential was reported previously for “*Candidatus* Poribacteria” and SAUL, typical symbionts of HMA sponges, and we propose here that HMA sponge symbionts collectively engage in degradation of dissolved organic matter, both labile and recalcitrant. Thus, sponge microbes may not only provide nutrients to the sponge host, but they may also contribute to dissolved organic matter (DOM) recycling and primary productivity in reef ecosystems via a pathway termed the sponge loop.

**IMPORTANCE** *Chloroflexi* represent a widespread, yet enigmatic bacterial phylum with few cultivated members. We used metagenomic and single-cell genomic approaches to characterize the functional gene repertoire of *Chloroflexi* symbionts in marine sponges. The results of this study suggest clade-specific metabolic specialization and that *Chloroflexi* symbionts have the genomic potential for dissolved organic matter (DOM) degradation from seawater. Considering the abundance and dominance of sponges in many benthic environments, we predict that the role of sponge symbionts in biogeochemical cycles is larger than previously thought.

## Abstract (precis)

*Chloroflexi* represent a widespread, yet enigmatic bacterial phylum with few cultivated members. We used metagenomic and single-cell genomic approaches to characterize the functional gene repertoire of *Chloroflexi* symbionts in marine sponges. The results of this study suggest clade-specific metabolic specialization and that *Chloroflexi* symbionts have the genomic potential for dissolved organic matter (DOM) degradation from seawater. Considering the abundance and dominance of sponges in many benthic environments, we predict that the role of sponge symbionts in biogeochemical cycles is larger than previously thought.

## INTRODUCTION

Sponges (*Porifera*) represent one of the oldest, still extant animal phyla. Fossil evidence shows their existence in the Precambrian long before the radiation of all other animal phyla (1, 2). Nowadays, sponges are globally distributed in all aquatic habitats from warm tropical reefs to the cold deep sea and are even present in freshwater lakes and streams (3). Sponges are increasingly recognized as important components of marine environments due to their immense filter-feeding capacities and consequent impacts upon coastal food webs and biogeochemical (e.g., carbon, nitrogen) cycles (4, 5). Many marine sponges contain dense and diverse microbial consortia within their extracellular mesohyl matrix. To date, 41 bacterial phyla (among them many candidate phyla) have been recorded from sponges, with recent amplicon sequencing studies suggesting up to 14,000 operational taxonomic units (OTUs) per sponge individual (6, 7). Sponges also constitute one of the most abundant natural sources of secondary metabolites, which are of commercial interest for the development of pharmaceuticals and new drugs (8) and are often produced by the microbial symbionts (9, 10).

Sponges can be classified into the so-called high-microbial-abundance (HMA) sponges harboring dense and diverse microbial consortia within their mesohyl tissues and the low-microbial-abundance (LMA) sponges containing microbial numbers on the order of those found in seawater (11–13). While HMA sponges are enriched in *Chloroflexi*, *Acidobacteria*, and “*Candidatus* Poribacteria,” the LMA sponges are dominated by *Gamma-* and *Betaproteobacteria* as well as *Cyanobacteria*, while *Chloroflexi* are typically absent. Differences have also been observed with respect to functional gene content (14), pumping rates (15), and exchange of carbon and nitrogen compounds (16). There is mounting evidence that HMA sponges are specialized to feed on dissolved organic matter (DOM), while the LMA sponges preferably feed on particulate organic matter (POM) (7, 17, 18). It is thus tempting to speculate that the symbiotic microbiota of HMA sponges is involved in DOM degradation, and indeed, the microbiomes analyzed so far encode a diverse repertoire for carbon metabolism pathways and transporters for low-molecular-weight compounds (10, 19–21). However, the precise fluxes and mechanisms how DOM and POM are taken up and processed within the sponge holobiont remain unknown. Recently, it was proposed that members of the phylum *Chloroflexi* are involved in recalcitrant DOM recycling in the water column (22, 23).

In the present study, we focused our metagenomic analyses on *Chloroflexi* as abundant and characteristic, yet understudied members of HMA sponge microbiota. The phylum *Chloroflexi* comprises taxonomically and physiologically highly diverse lineages that populate a wide range of habitats (24–27) including the deep sea (22), uranium-contaminated aquifers (28), and the human oral cavity and gut (29, 30). *Chloroflexi* metabolism is very diverse, ranging from anoxygenic photosynthesizers, obligate aerobic/anaerobic heterotrophs, thermophiles, halophiles, clades capable of reductive halogenation, and even predators with gliding motility. Because only a few *Chloroflexi* lineages have been cultivated (87) and because draft genomes are limited in number (22, 23, 32), the specific functions of *Chloroflexi* within the marine ecosystem but especially in the symbiont context remain largely unknown.

*Chloroflexi* are members of HMA sponge microbiota, with representatives of classes/clades SAR202, *Anaerolineae*, and *Caldilineae* being the most abundant (31). Visualization of *Chloroflexi* by fluorescence *in situ* hybridization (FISH) revealed bright and abundant signals (32, 33). Because *Chloroflexi* likely play an important role in the HMA sponge holobiont, we had the following aims: (i) to assess their relative abundances and distributions in diverse HMA sponge species by using the largest data set currently available (Earth Microbiome Project [EMP] sponge microbiome [31]), (ii) to provide their phylogenetic affiliation, (iii) to characterize the functional gene repertoire with a particular focus on carbon degradation and symbiotic lifestyle, and (iv) to visualize *Chloroflexi* in mesohyl tissues at ultrastructural resolution by FISH-correlative light and electron microscopy (CLEM) methodology. We applied a broad range of state-of-the-art methods, from global sponge surveys to single-cell genomics and microscopy, to acquire comprehensive insights into the lifestyle of *Chloroflexi* symbionts.

## RESULTS AND DISCUSSION

### *Chloroflexi* abundance in HMA sponges.

**FIG 1:**
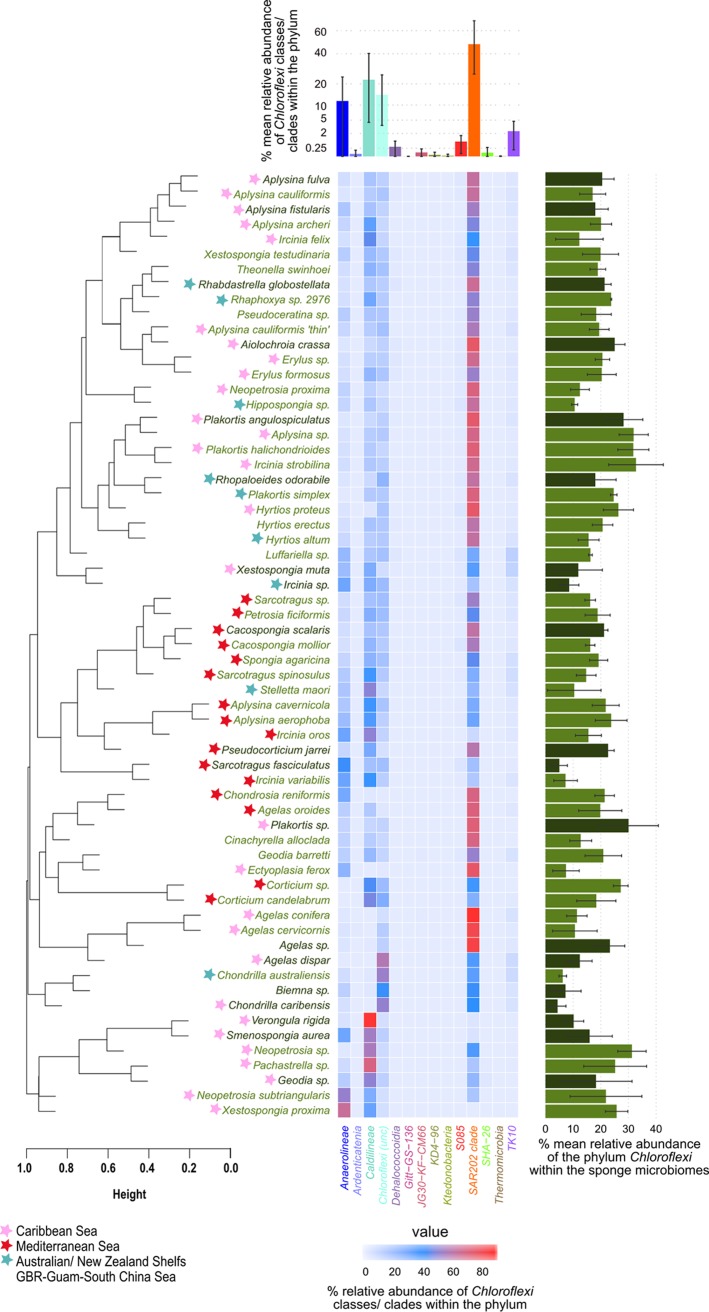
Heatmap showing the relative abundance of *Chloroflexi* classes/clades in 63 HMA sponges extracted from Earth Microbiome Project (EMP) data (31). The top panel shows the mean relative abundance of *Chloroflexi* classes/clades in all sponges (means ± standard deviations [error bars]). The right panel displays the mean relative abundance of the phylum *Chloroflexi* in predicted (light green names and medium green bars) and classified (dark green names and bars) HMA sponges (means ± standard deviations) determined by machine learning (12). Results of cluster analysis based on Bray-Curtis dissimilarities on mean relative abundances of OTUs within the phylum are presented on the left side. Sponges are marked with stars when all species samples came from one of the three major sample locations.

Recently, members of the phylum *Chloroflexi* were shown to be present in much higher abundance and diversity in HMA sponges than in LMA sponges, which is why they were termed indicator species for HMA sponges (12). Here, we provide further details for the presence and abundance of *Chloroflexi* in HMA sponges (12) (Fig. 1; see also Tables S2A and S2B in the supplemental material). The recently compiled Sponge Microbiome Project (6, 31) was used as a reference database. In these 63 investigated sponge species, *Chloroflexi* abundances ranged from 4.39% ± 3.02% (Chondrilla caribensis) to 31.89% ± 5.27% (*Aplysina* spp.) (Fig. 1, right panel, and Table S2A). With respect to the *Chloroflexi* classes, the SAR202 clade was the most abundant, contributing on average to 47.74% ± 22.00% of the phylum total abundance (Fig. 1, top panel, and Table S2B). Members of the classes *Caldilineae* (22.35% ± 17.93%) and *Anaerolineae* (11.64% ± 12.30%) were also abundant in some sponges but not others. Unclassified OTUs at the class level represented 14.50% ± 10.77% of *Chloroflexi* sequences, indicating that there is phylogenetic novelty still to be discovered. Despite some variability (Fig. 1, heatmap), the classes/clades SAR202, *Caldilineae*, and *Anaerolineae* as well as diverse hitherto unclassified OTUs dominated the *Chloroflexi* population in the HMA sponges. The remaining classes/clades amounted to 3.78% of total phylum abundance. At the OTU level, there was a significant effect of geographical location [F (15, 725) = 15.9, *P* = 0.001] and of sponge taxonomy [F(9, 725) = 18.6, *P* = 0.001] on beta diversity (Table S2C). For example, a Mediterranean cluster and several Caribbean clusters become visible as shown by Bray-Curtis cluster analysis. In addition, a host taxonomic signature was revealed for example for the sponge genera *Aplysina* and *Agelas*. However, there were also exceptions (i.e., *Neopetrosia* species from the Caribbean did not cluster together, and Stelletta maori from New Zealand fell into the Mediterranean cluster). Altogether, this analysis showed the high abundance and consistency of the three above-mentioned main clades of *Chloroflexi* in HMA sponges and revealed a geographic and host taxonomic signature for the *Chloroflexi* community.

### Phylogeny of *Chloroflexi* metagenome bins and single amplified genomes.

**FIG 2:**
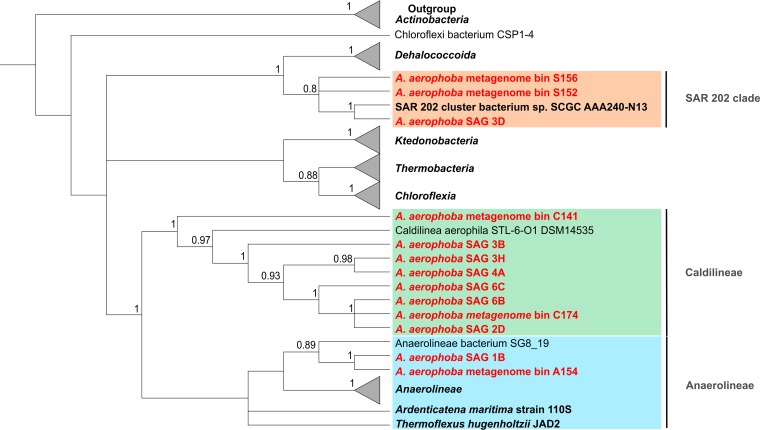
Concatenated protein tree. Maximum likelihood phylogenetic analysis of *Chloroflexi* metagenome bins and SAGs (in red) from 1,914 positions of 60 sequences using ribosomal proteins. The percentage of replicate trees in which the associated taxa clustered together in the bootstrap test (100 replicates) are shown. The initial tree for the heuristic search was obtained automatically by applying neighbor-joining and BioNJ algorithms to a matrix of pairwise distances estimated using a JTT model. Multiple sequences are included in the collapsed branches representing *Chloroflexi* classes/clades (bold). To root the tree, three representative genomes from the phylum *Actinobacteria* were used. Reference genomes with accession numbers can be found in Table S1 in the supplemental material.

**FIG 3:**
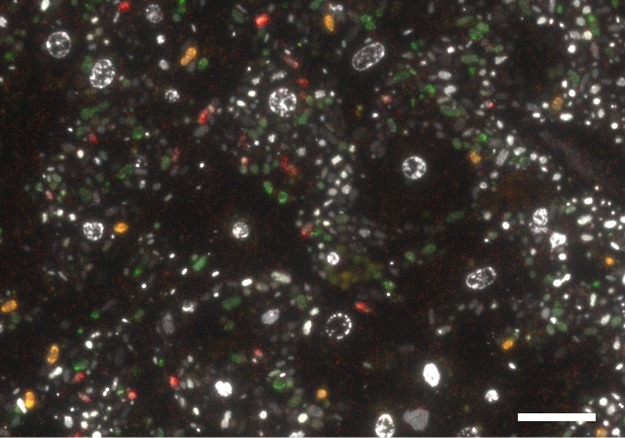
Distribution of *Chloroflexi* clades in *Aplysina aerophoba* mesohyl using fluorescence *in situ* hybridization (FISH). The image shows the overlay of all probes. SAR202 cells are displayed in green, *Caldilineae* cells in orange, and *Anaerolineae* cells in red. The nucleotide stain DAPI (white/gray) served as a reference for the localization of unstained cells. Bar, 10 µm.

In total, 260 single amplified genomes (SAGs) were screened for the presence of the phylogenetic marker. A total of 125 16S rRNA genes were identified in 112 SAGs, of which some were duplicates per well. Sequencing revealed that 39 genes (31.2%) were affiliated with the phylum *Chloroflexi* (33). We randomly chose 13 SAGs for genome sequencing. Phylogenetic analysis of 16S rRNA genes revealed that one SAG (3D), four SAGs (1B, 1G, 1H, and 4H), and eight SAGs (2D, 3B, 3H, 4A, 5H, 6B, 6C, and 6F) belonged to the classes/clades SAR202, *Anaerolineae*, and *Caldilineae*, respectively, forming a well-supported sequence cluster (bootstrap, 100) with other sponge-derived sequences (Fig. S2). The binning of metagenomic sequence data (21) resulted in additional five high-quality bins (Table 1). The only metagenome bin containing a 16S rRNA gene (S156) belonged to SAR202. The SAR202 sequences formed a well-supported cluster (bootstrap value, 98) with other sponge-derived 16S rRNA gene sequences (Fig. S1). To elucidate the clade affiliation of the remaining four metagenome bins lacking 16S rRNA genes of appropriate length, a concatenated genome tree based on nine ribosomal genes was calculated (Fig. 2). One bin (A154) was affiliated with the class *Anaerolineae*, two metagenome bins were associated with the *Caldilineae* (C141 and C174), and two bins were associated with the SAR202 clade (S152 and S156) within the phylum *Chloroflexi*. The phylogenetic affiliation of metagenome bin S156 was congruent with the 16S rRNA gene analysis. SAGs were included in the protein-based phylogenetic analysis when they encoded at least three of the nine ribosomal genes. Due to the lack of more-complete reference genomes from SAR202 microorganisms, the most complete one (ca. 25%, SAR202 cluster bacterium sp. strain SCGC AAA240-N13, IMG Gold Study ID Gs0017605 [22]) was included in this analysis, although only one ribosomal protein could be used for tree construction. Both analyses showed a stable phylogeny of all SAGs and metagenome bins to above-described classes or clades within the phylum *Chloroflexi*. All three classes/clades were visualized in the A. aerophoba sponge mesohyl matrix by fluorescence *in situ* cohybridization (FISH) on ultrathin tissue sections using class/clade-specific probes. The *Chloroflexi* cell signal was abundant as judged by the stained versus unstained bacterial signal, and SAR202 (green) seemed to dominate over either *Anaerolineae* (red) or *Caldilineae* (orange). The cells were metabolically active as judged by the brightness of the FISH probe (Fig. 3). These visual observations are consistent with the relative abundance of the phylum *Chloroflexi* within sponge microbiomes (24% ± 6% within *A. aerophoba* sponge microbiome; Fig. 1 and Table S2A) and the representation of *Chloroflexi* cells among SAGs (31%).

**TABLE 1:**
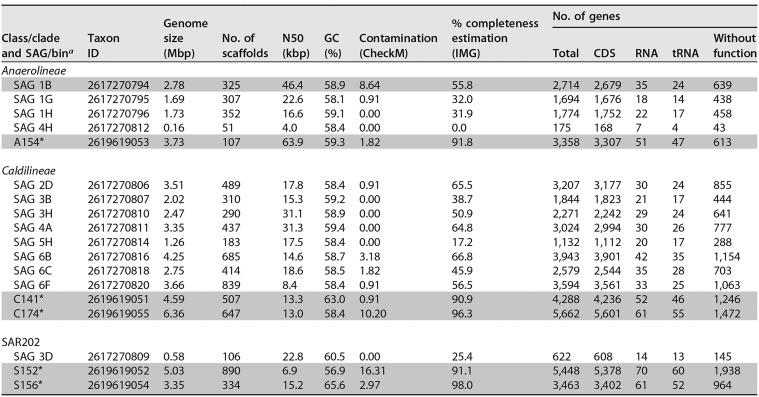
Genomic features overview of single amplified genomes (SAGs) and metagenome bins of *A. aerophoba* associated *Chloroflexi* and closest relative reference genomes analyzed in this study

a IMG Gold Study IDs are Gs0114494 and Gs0099546 (marked with an asterisk). The letters of the bins reflect the phylogenetic identity of the bin (A for *Anaerolineae*, C for *Caldilineae*, and S for SAR202). The gray shaded bins/SAGs were used for further detailed metabolic analysis.

10.1128/mSystems.00150-18.1 FIG S1 Pairwise probe testing for FISH-CLEM visualization. FISH-CLEM visualization using *A*. *aerophoba*-associated microbe separations SAR202-specific probe in blue and *Caldilineae* in green (A), SAR202 (blue) and *Anaerolineae* (red) (B), and *Anaerolineae* (red) and *Caldilineae* (green) (C). Test hybridizations were done at 10%, 20%, and 30% FA concentration with no observed colocalization of probes. cy, cyanobacterial autofluorescence overlapped with the blue signal of the SAR202 probe. Download FIG S1, TIF file, 0.10 MB. Copyright © 2018 Bayer et al. 2018 Bayer et al. This content is distributed under the terms of the Creative Commons Attribution 4.0 International license.

10.1128/mSystems.00150-18.2 FIG S2 16S rRNA gene-based phylogenetic tree of all 13 single amplified genomes (SAGs) of sponge-associated *Chloroflexi* and one metagenome bin (S156) shown in red. The phylogenetic affiliation was reconstructed by the neighbor-joining method from 182 sequences and a maximum of 1,787 positions (with gaps) (1,500 positions without gaps) and the GTR + G + I model in MEGA7.0. Red clusters represent sponge-specific *Chloroflexi* cluster, whereas light gray clusters are built by nonsymbiotic *Chloroflexi* reference sequences. Blue clusters are built from seawater-derived samples. Gray clusters are built from uncultured clone sequences (light gray) or cultured representatives (dark gray). Download FIG S2, TIF file, 0.9 MB. Copyright © 2018 Bayer et al. 2018 Bayer et al. This content is distributed under the terms of the Creative Commons Attribution 4.0 International license.

10.1128/mSystems.00150-18.5 TABLE S1 References used for protein tree based on ribosomal genes. Download Table S1, DOCX file, 0.02 MB. Copyright © 2018 Bayer et al. 2018 Bayer et al. This content is distributed under the terms of the Creative Commons Attribution 4.0 International license.

10.1128/mSystems.00150-18.6 TABLE S2 (A) Mean relative abundances of the phylum *Chloroflexi* in all investigated HMA sponges. (B) Mean relative abundances of *Chloroflexi* classes in all investigated HMA sponges. (C) Effect of sponge geographic region and taxonomic order on *Chloroflexi* communities. Download Table S2, DOCX file, 0.02 MB. Copyright © 2018 Bayer et al. 2018 Bayer et al. This content is distributed under the terms of the Creative Commons Attribution 4.0 International license.

### General description of genomes.

The final genome assembly sizes for the sponge-associated *Chloroflexi* single cells (SAGs) ranged from 0.16 to 4.25 Mbp, representing up to 66.85% of genome completeness derived from IMG-based estimations (Table 1). The guanine-cytosine (GC) content ranged from 58.08 to 59.32%, 58.36 to 62.98%, and 56.93 to 65.59% for *Anaerolineae*, *Caldilineae*, and SAR202, respectively. The numbers of identified genes were highly variable, ranging from 3,358 genes for *Anaerolineae* genome bin A154 to 5,448 for the SAR202 bin S152 and 5,662 genes for the *Caldilineae* bin C174 (Table 1). The five metagenome bins (two for *Caldilineae* [C141 and C174], two for SAR202 [S152 and S156], and one for *Anaerolineae* [A154]) which had >90% coverage were chosen for detailed metabolic analysis and inner-phylum comparison. The letters of the bins were chosen to reflect their phylogenetic identity (A for *Anaerolineae*, C for *Caldilineae*, and S for SAR202). Additionally, the most complete *Anaerolineae* SAG 1B (55.76% genome completeness estimation) was included in the analysis. Due to the lack of complete genomes of marine representatives of the *Anaerolineae*, *Caldilineae*, and SAR202 available at the time of analysis, sponge-derived *Chloroflexi* genomes could not be fully assessed. Taking the pitfalls inherent to metagenome sequencing into account (i.e., fragmented assemblies, unresolved ambiguities), we have opted for a presence/absence approach: a gene or enzyme was considered present when it was identified in both bins of the corresponding clade. For *Anaerolineae*, we consider an enzyme or gene present when identified in bin A154, and the SAG 1B was taken as additional support.

### Central metabolism of sponge-associated *Chloroflexi*.

Metabolic reconstruction suggests that *Chloroflexi* are aerobic and heterotrophic bacteria including glycolysis, tricarboxylic acid (TCA) cycle, pentose phosphate pathway (PPP) (see Text S1 and Fig. S3A to C for details), and the respiratory chain as energy-producing pathways in all three clades.

10.1128/mSystems.00150-18.3 FIG S3 IMG-based pathway reconstruction. Energy gaining/consuming steps and connections to other pathways are displayed. Filled pies mean that corresponding enzymes were identified/annotated. Empty pies mean that enzymes were not identified. Numbers are KEGG identifiers for enzymes. Gray arrows and numbers reflect that the enzyme was not identified. Colors represent the six genomes. (A) Glycolysis followed by pyruvate oxidation to acetyl-CoA and acetate formation. (B) Tricarboxylic acid (TCA) cycle. (C) Pathway reconstruction of sugar conversions in the pentose phosphate pathway (PPP). (D) Pathway reconstruction of autotrophic carbon fixation pathways, including the Wood-Ljungdahl (reductive acetyl-CoA pathway) pathway and the Arnon-Buchanan-pathway (reductive citrate acid cycle). (E) Nitrogen metabolism. (F) Sulfur metabolism. fd, ferredoxin; Q, quinone; HQ, hydroquinone. Download FIG S3, TIF file, 0.6 MB. Copyright © 2018 Bayer et al. 2018 Bayer et al. This content is distributed under the terms of the Creative Commons Attribution 4.0 International license.

10.1128/mSystems.00150-18.10 TEXT S1 Supporting text on metabolic features. Detailed descriptions of central metabolism, sugar transport and metabolism, import and biosynthesis of cofactors and vitamins, amino sugar and nucleotide sugar metabolism, nucleotide metabolism, amino acid biosynthesis and metabolism (including degradation), fatty acid metabolism, peptidoglycan biosynthesis, and degradation of aromatic compounds. Download Text S1, PDF file, 2.0 MB. Copyright © 2018 Bayer et al. 2018 Bayer et al. This content is distributed under the terms of the Creative Commons Attribution 4.0 International license.

With respect to autotrophic carbon fixation, the reductive citrate acid cycle (Arnon-Buchanan cycle), which is largely present, and the Wood-Ljungdahl pathway, which was partially identified (Fig. S2D) might be functional in times when sponges are not pumping and the mesohyl turns anoxic (34). Genes encoding components involved in ammonia import and assimilation are carried on all investigated genomes, but SAR202 and *Caldilineae* have additional genes for glutamate synthesis from glutamine and directly from ammonia. The transport of nitrite (and possibly also nitrate) is encoded on all investigated genomes, while the reduction to ammonia is encoded only by SAR202 (Fig. S3E). The incorporation of sulfur (with, e.g., thiosulfate as donor) into S-containing amino acids might be possible in all clades, whereas the assimilatory reduction of sulfate is restricted to *Anaerolineae* and *Caldilineae* genomes (Fig. S3F).

These processes additionally provide precursors for further metabolic pathways such as biosynthesis of purines and pyrimidines, amino acids, and cofactors, or structural compounds. Machinery for transcription, translation, and purine and pyrimidine metabolism are largely present. Fatty acid (FA) biosynthesis and degradation pathways were detected in all six genomes. All genomes encode a high number of different ABC transporters compared to genomes of free-living bacteria (22, 35) to supplement for nutrition and cell growth-related compounds [including oligopeptides, phosphate, l- and branched-chain amino acids, minerals such as iron(III) and molybdate, metal ions such as zinc, manganese, and iron(II)]. Additionally, all six genomes largely encode enzymes needed for biosynthesis of most amino acids (Text S1). We could not identify any of the typical phosphotransferase systems, as was the case for *Ca.* Poribacteria described previously (19).

We found genomic potential for aromatic degradation in *Chloroflexi* genomes, but pathways remain incomplete (Text S1). Several genes encoding components involved in phenylpropionate and cinnamate degradation, terephthalate degradation, catechol degradation, and xylene degradation were identified in *Chloroflexi* genomes. Also, genes encoding enzymes involved in ring cleavage by Baeyer-Villinger oxidation and beta oxidation as well as ring-hydroxylating dioxygenases and isomerases were identified which could be involved in degradation of aromatic compounds possibly synthesized by the sponge host or other microbes. This finding is interesting in the context that many sponge species contain secondary metabolites that often contain aromatic ring structures that serve as a defense strategy against predators and biofouling (36). Sponge symbionts may be able to degrade such substances, enabling them to a life within sponge hosts. These findings fit with the potential degradation of organic compounds which was suggested for *Chloroflexi* bacteria of the class SAR202 by (meta)genomic studies (22, 23, 35). The highest (20 to 30% relative to the total microbiome) and most consistent presence of *Chloroflexi* within a sponge genus was found in the sponge genera *Plakortis*, *Agelas* (with the exception of Agelas dispar), and *Aplysina* and sister taxon *Aiolochroia.* Interestingly, all of them contain characteristic natural products with aromatic ring structures. It is therefore tempting to speculate that the presence and abundance of *Chloroflexi* and especially SAR202 are shaped, at least to some extent, by the natural products present in the corresponding host sponges.

With respect to cell wall structure, the *Anaerolineae* and *Caldilineae* genomes carry the gene repertoire for peptidoglycan biosynthesis. The noticeable lack of peptidoglycan biosynthesis genes in the SAR202 genomes (Text S1) is consistent with previous analyses of three *Chloroflexi* genomes derived from uranium-contaminated aquifers (28) and hyperoxic zones from the Gulf of Mexico (37). Additionally, consistent with previous observations (38), none of the six genomes contained flagellar or chemotaxis genes.

### Metabolic specialization: extensive carbohydrate uptake and degradation in *Anaerolineae* and *Caldilineae*.

**FIG 4:**
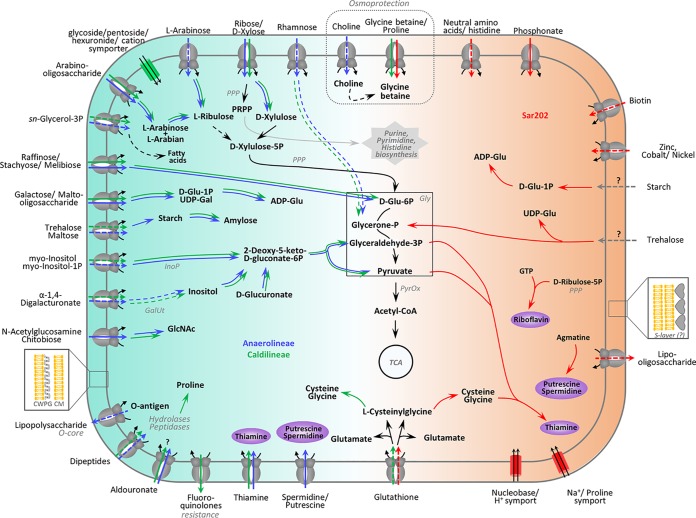
Summarized metabolic features which were found only in *Anaerolineae* and *Caldilineae* (left side, blue and green arrows, respectively) or in SAR202 genomes (right side, red arrows). The central metabolic pathways (glycolysis, TCA cycle, purine, pyrimidine histidine biosynthesis) located in the middle of the figure are general features found in all genomes. Lines are dashed when pathways or transporter could not be annotated completely (single enzymes of the pathway or single genes from the transporter were missing) or could not be annotated in both genomes of one clade. Gray dashed arrows indicate that those transporters were not identified.

The following features are metabolic specialties of *Anaerolineae* and *Caldilineae* and appear to be missing in SAR202, unless otherwise mentioned (Fig. 4). We found a number of ABC transporters for the import of diverse monosaccharides (ribose/xylose, inositol, glycerol-3-phosphate [glycerol-3P], and rhamnose) and oligosaccharides (sorbitol, raffinose/stachyose/melibiose, maltose, *N*-acetylglucosamine, and arabinosaccharide) into *Anaerolineae* and *Caldilineae* cells. Xylose can be processed to xylulose which may enter the pentose phosphate cycle finally leading into glycolysis. Ribose can be converted to 5-phosphoribosyl 1-pyrophosphate (PRPP), which is a precursor for the biosynthesis of the amino acid histidine or it may fuel purine and pyrimidine synthesis (Text S1). Both groups may also be able to import glycerol-3P, which is a phosphoric ester of glycerol (a component of glycerophospholipids) which can be converted to fatty acids. Additionally, we found evidence for arabinose and rhamnose import and degradation; however, annotation was incomplete (Text S1).

Arabinooligosaccharides (such as α-l-arabinofuranosides, α-l-arabinans, arabinoxylans, and arabinogalactans) result from degradation of plant-like cell material entering the sponge by filtration. These substances may be imported by the almost completely annotated AraNPQ and MsmX transporters and be utilized to l-arabian and l-arabinose by the enzyme α-*N*-arabinofuranosidase (EC 3.2.1.55, GH3). The enzyme l-arabinose isomerase (EC 5.3.1.4, AraA) is present in all four genomes of *Anaerolineae* and *Caldilineae* and converts l-arabinose to l-ribulose, which can further be converted by reactions of PPP to glucose-6P suitable for entering glycolysis. Additionally, other oligosaccharides such as stachyose, raffinose, melibiose, and galactose can be imported and used in central metabolism (Fig. 4 and Text S1).

The utilization of *myo*-inositol as a carbon source and possibly as a regulatory agent was hypothesized previously for sponge-associated *Ca.* Poribacteria (19). Similarly, sponge-associated *Anaerolineae* and *Caldilineae* genes encode almost all the components involved in the inositol degradation pathway (Text S1). *myo*-Inositol is likely degraded to glyceraldehyde-3-phosphate and acetyl-CoA, which are further used in the central metabolism. Inositol phosphates are found as part of eukaryotic and archaeal cell wall components (39). Phosphorylated inositol is a precursor for several lipid molecules, including sphingolipids, ceramides, and glycosylphosphatidylinositol anchors (40), as well as many stress-protective solutes of eukaryotes (39), and it might be part of the signal transduction in sponges (41). Therefore, the sponge itself or eukaryotic microorganisms can probably provide inositol as a carbon source or regulatory agent for the microbial symbionts.

Uronic acids are sugar acids that can be found in biopolymers of plants, animals, and bacteria (42, 43) and are known to occur in glycosaminoglycans (GAGs). GAGs in sponges are mainly composed of fucose, glucuronic acid (glucoronate), mannose, galactose, *N*-acetylglucosamine, and sulfate (44–46). Genes encoding enzymes involved in degradation of uronic acids were found in *Anaerolineae* and *Caldilineae* genomes. The possibility of galacturonate and glucuronate catabolism is supported by the conversion of 2-dehydro-3-deoxy-d-gluconate by the enzymes glucoronate isomerase (EC 5.3.1.12), tagaturonate reductase (EC 1.1.1.58), and altronate hydrolase (EC 4.2.1.7). Furthermore, the presence of genes encoding oligogalacturonide lyase (EC 4.2.2.6), 2-deoxy-d-gluconate 3-dehydrogenase (EC 1.1.1.125), and 2-dehydro-3-desoxy-d-glucokinase (EC 2.7.1.45) supports possible 4(4-α-d-gluc-4-enuronosyl)-d-galacturonate degradation activity. The products could then enter the Entner-Doudoroff (ED) pathway via 2-dehydro-3-desoxyphophogluconate aldolase (EC 4.1.2.14). Uronic acid degradation could principally be connected to the inositol degradation pathway via d-galacturonate even though additional genome evidence, such as genes encoding the enzyme inositol oxidase (EC 1.13.99.1), remain wanting (Fig. 4 and Text S1). A number of transporters for *N*-acetylglucosamine, digalacturonate, mannose, and galactose (Fig. 4 and Text S1) were identified in *Anaerolineae* and *Caldilineae* genomes. Digalacturonate can be utilized by the uronic acid degradation pathway (Text S1), and *N*-acetylglucosamine can be used directly in amino sugar and nucleotide sugar synthesis. The presence of uronic acid degradation pathways provides strong support that *Anaerolineae* and *Caldilineae*, similar to the previously described *Ca.* Poribacteria, degrade glycosaminoglycan chains of proteoglycans, which are important components of the sponge host matrix (19). In that line, *Anaerolineae* and *Caldilineae* genomes were enriched in arylsulfatases A (Fig. S4A), which are thought to be involved in metabolization of sulfated polysaccharides from the sponge extracellular matrix (19, 21) and in the heterotrophic ability of symbionts to use sponge components for nutritional purposes.

10.1128/mSystems.00150-18.4 FIG S4 Total numbers of arylsulfatases (and other sulfatases) (A), carbohydrate active enzymes (CAZymes) (B), and eukaryotic-like proteins (ELPs) (C) in the six investigated sponge-associated *Chloroflexi* genomes belonging to classes *Anaerolineae* (SAG1B and A154), *Caldilineae* (C141 and C174), and SAR202 group (S152 and S156). Colors represent the six genomes. AA, auxiliary activities; CBM, carbohydrate-binding modules; CE, carbohydrate esterases; GH, glycoside hydrolases; GT, glycosyltransferases; PL, polysaccharide lyases. Download FIG S4, TIF file, 0.1 MB. Copyright © 2018 Bayer et al. 2018 Bayer et al. This content is distributed under the terms of the Creative Commons Attribution 4.0 International license.

### Expanded carbohydrate-active enzyme (CAZymes) repertoire in *Caldilineae* and *Anaerolineae*.

In order to search for CAZymes, we screened the *Chloroflexi* genome data against dbCAN (47) and classified the enzymes according to the CAZy database (48). Most *Chloroflexi* hits were against glycosyl hydrolases (GH), glycosyltransferases (GT), and carbohydrate-binding modules (CBM). Consistent with the above-described metabolic specializations, these enzyme classes were present in larger amounts in *Caldilineae* and *Anaerolineae* than in SAR202 (Fig. S4B and Table S3). Altogether, 40 GH families were identified in all *Chloroflexi* genomes (Table S3). Glycosyl hydrolase family 109 was the most abundant family of GHs and was identified in all six genomes. GH109 family proteins are predicted as α-*N*-acetylgalactosaminidases (EC 3.2.1.49) with putative substrates such as glycolipids, glycopeptides, and glycoproteins, all of which are common constituents of sponge mesohyl as well as dissolved organic matter from seawater. The GH74 family is the second most abundant and is also present in all six genomes. These appear to be xyloglucan-hydrolyzing enzymes, that act on β-1,4 linkages and might help degrade various oligo- and polysaccharides. The previously reported glycosylhydrolases GH33 and GH32 (19, 20) were the third most abundant, but they were restricted to *Caldilineae* bin C174. This enzyme family is annotated as sialidase (EC 3.2.1.18), capable of hydrolyzing glycosidic linkages of terminal sialic acid residues, which are present in sponge mesohyl (49). Altogether, 17 glycosyltransferases were identified on *Chloroflexi* genomes with families GT2, GT4, and GT83 being the most abundant. Among the 11 CBM families identified on *Chloroflexi* genomes, CBM50 was the most abundant, but it was restricted to *Caldilineae* and *Anaerolineae*. CMB50 modules, also known as LysM domains, attach to various GH enzymes which are involved in the cleavage of chitin or peptidoglycan. The numbers of carbohydrate-active enzymes on *Chloroflexi* symbiont genomes reflect their extensive potential to degrade complex carbohydrates as reported previously for *Ca.* Poribacteria and the sponge-associated unidentified lineage SAUL (19, 20).

10.1128/mSystems.00150-18.7 TABLE S3 Absolute abundance of carbohydrate active enzymes (CAZymes) detected in the six analyzed genomes. Download Table S3, DOCX file, 0.04 MB. Copyright © 2018 Bayer et al. 2018 Bayer et al. This content is distributed under the terms of the Creative Commons Attribution 4.0 International license.

### Metabolic specialization: cofactor biosynthesis in SAR202 genomes.

**FIG 5:**
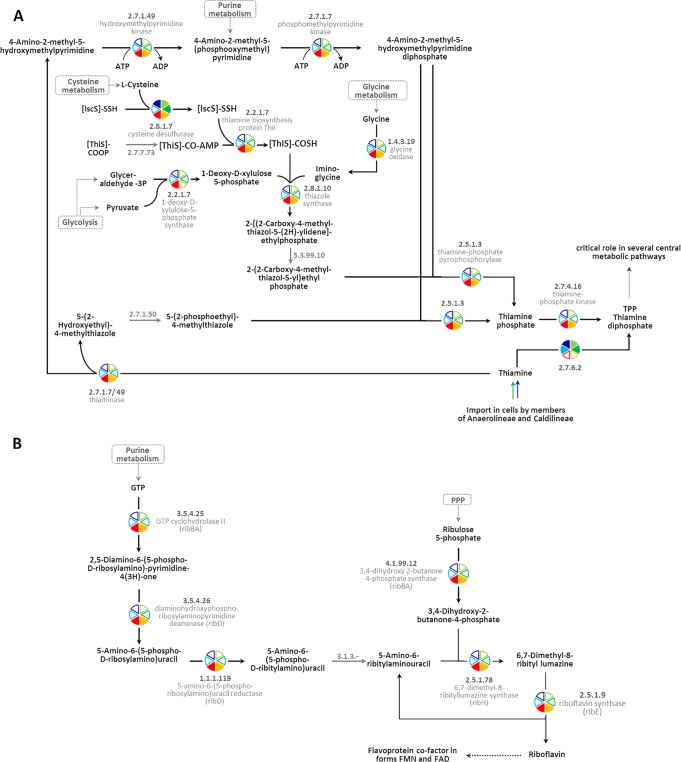
Pathway for synthesis of thiamine (A) and riboflavin (B) in both SAR202 genomes. (A) Members of classes *Anaerolineae* and *Caldilineae* carry genes encoding components involved in the import of thiamine (see also Fig. 4). (B) The conversion of riboflavin into the biologically active forms (flavin mononucleotide [FMN] and flavin adenine dinucleotide [FAD]) was encoded by genes in the genomes of all three classes (filled pies compared to empty pies). The colors represent the genomes. The numbers are KEGG identifiers. PPP, pentose phosphate pathway. Gray arrows and numbers indicate unidentified enzymes.

There is mounting evidence that vitamins and cofactors produced by diverse symbiont lineages could be beneficial to the sponge host (50–53). Parallel transcriptional activity profiling of the symbionts and the sponge showed that the symbionts had the capacity for vitamin B biosynthesis, whereas the host transcripts displayed the capacity for vitamin catabolism (54). It is thus tempting to speculate that the sponges’ nutrition is augmented by symbiont-derived vitamins and cofactors. In the present study, at least two biosynthetic pathways for cofactor biosynthesis were identified on SAR202 genomes, which were absent in *Anaerolineae* and *Caldilineae* (Fig. 5). Thiamine is an essential cofactor which is involved in central metabolism. The biosynthesis of the biologically active form thiamine diphosphate (TPP) from l-cysteine, glycine, pyruvate, and glyceraldehyde-3P is encoded on the SAR202 genomes (Fig. 4 and 5A). Although the pathway is incomplete, the data strongly suggest that the synthesis of TPP is restricted to SAR202 bacteria. Instead, *Anaerolineae* and *Caldilineae* appear to import thiamine via an ABC transporter (TbpA, ThiPQ) and convert it to TPP by using thiamine pyrophosphokinase (EC 2.7.6.2). Additionally, thiamine synthesis might be a symbiosis-related feature, since free-living SAR202 members lack the synthesis ability (22, 37).

Second, riboflavin (vitamin B2) is required by enzymes and proteins to perform certain physiological functions. Specifically, the active forms, flavin mononucleotide (FMN) and flavin adenine dinucleotide (FAD), serve as cofactors for a variety of flavoprotein enzyme reactions. Most genomes carry genes encoding the enzymes FMN adenylyltransferase (EC 2.7.7.2) and FAD riboflavin kinase (EC 2.7.1.26) which activate riboflavin into FMN. However, only the SAR202 genomes contain genes encoding the riboflavin biosynthesis enzymes which rely on GTP and ribulose-5P (Fig. 4 and 5B). Both substrates can be provided by pathways of the central metabolism (purine metabolism and PPP). Only one free-living SAR202 bacterium (unclassified *Chloroflexi* bin 43) (37) has genes encoding components involved in riboflavin synthesis.

### Potential for degradation of recalcitrant DOM in SAR202.

The uptake of the amino acid l-Asp was shown before in subtropical Atlantic waters (35), and the possible participation of deep sea SAR202 bacteria in degradation of recalcitrant or refractory DOM was recently postulated by Landry et al. (22). Even though the exact composition of DOM in the world’s oceans remains to be elucidated, refractory DOM is an important component of the global carbon budget in terms of sheer mass. Landry et al. (22) argue that SAR202 genomes have an expanded repertoire of oxidative enzymes that may help in the oxidation of recalcitrant compounds. Interestingly, some of the described enzymes were also found to be enriched in SAR202 symbionts of sponges (Table S4). Among them are genes encoding proteins from the CaiB/BaiF family as well as related family III transferases. While the enrichment of CaiB was previously interpreted as carnitine being a carbon and nitrogen source for sponge symbionts (21), an alternative explanation may be that it serves to funnel substrates into degradation pathways without consumption of energy by shuffling CoA, thus generating free electrons (Text S1). Even though the precise function of CaiB/BaiF family proteins cannot be elucidated at this time, the enrichment in SAR202 genomes is noteworthy. Further, a total of 53 flavin-dependent, class C oxidoreductases of the luciferase family (flavin mononucleotide monooxygenases [FMNOs], COG2141) which include alkanesulfonate monooxygenase SsuD and methylene tetrahydromethanopterin reductase, were present and enriched in SAR202 genomes compared to the genomes of *Anaerolineae* and *Caldilineae* bacteria (Table S4). These enzymes are proposed to participate in the oxidation of (long-chain) aldehydes to carboxylic acids and/or cleavage of carbon-sulfur bonds in a variety of sulfonated alkanes (55). The SAR202 genomes contained 22 genes encoding short-chain alcohol dehydrogenases (COG0300) which might be involved in canalization of ketone body derivate release. Some of these genes from bin S152 showed homologies to cyclopentanol and 3-α (or 20-β)-hydroxysteroid dehydrogenases which convert alicyclic-bound alcohol groups to ketones (22, 56). The combination of the enzymes described above could allow sponge-associated *Chloroflexi* to convert recalcitrant alicyclic ring structures to more labile carboxylic acid, as proposed recently for SAR202 bacteria from deep sea (22).

10.1128/mSystems.00150-18.8 TABLE S4 Absolute abundance of enzymes possibly involved in recalcitrant DOM degradation. Download Table S4, DOCX file, 0.02 MB. Copyright © 2018 Bayer et al. 2018 Bayer et al. This content is distributed under the terms of the Creative Commons Attribution 4.0 International license.

Additionally, a number of genes encoding oxidative enzymes were identified on the *Chloroflexi* genomes but were not enriched in SAR202 (Table S4). These enzymes include a 2-oxoglutarate:ferrodoxin oxidoreductase (EC 1.2.7.11) which oxidizes acetyl-CoA, carbon monoxide dehydrogenase (EC 1.2.99.2) which might allow the bacteria to oxidize CO as described for some members of the Ktedonobacteria (57), CO- or xanthine dehydrogenases (COG1529) which are possibly involved in oxidation of a broad range of complex substrates (22), choline dehydrogenase (EC 1.1.99.1) being possibly involved in the oxidation of alcohols to aldehydes, sarcosine oxidase (EC 1.5.3.1), the serine hydroxymethyltransferase (2.1.2.1) with predicted function in choline degradation, formaldehyde dehydrogenase (EC 1.2.1.46), and subunits of formate dehydrogenase (EC 1.2.1.2/43) which oxidize formaldehyde and formate and might be involved in demethylation of various compounds. The overall presence and frequent enrichment of enzymes with oxidative capacity in SAR202 would be consistent with gene functions in degradation of recalcitrant DOM. However, owing to the sponges’ existence in shallow-water sun-lit benthic environments, it remains unclear whether the sponge symbionts encounter recalcitrant DOM derived from seawater sources. Interestingly, Colatriano et al. recently proposed the degradation of terrestrial DOM (tDOM) by members of the SAR202 clade (23). Shallow-water sponges and associated symbionts could be faced by tDOM via freshwater inflow in ocean waters they inhabit. Alternatively, and similar to other high-diversity microbiota, for example, of ant, ruminant, and human guts, the resident microbes were likely to specialize in certain substrates, thus promoting maximum nutrient exploitation and also securing their individual niche in the holobiont ecosystem.

### Symbiosis-related features.

Eukaryotic-like proteins (ELPs) seem to be a general genomic feature of sponge symbionts (20, 38, 50, 51, 58–60). Ankyrin (ANK), tetratricopeptide (TPR), and leucine-rich (LRR) repeat proteins are postulated to be involved in mediating host-microbe interactions (61, 62). Ankyrin and ankyrin repeat-containing proteins were detected in all six genomes of sponge symbionts (Fig. S4C and Table S5A) in higher numbers than in free-living bacteria (22, 37). It was recently proposed that the expression of sponge symbiont-derived ankyrin protein prevents phagocytosis by amoeba (63), and it is tempting to speculate that they protect the symbionts from digestion by the sponge archaeocytes *in vivo*. TPRs, possibly functioning as a module for protein-protein interaction involved in a variety of cellular functions, including those that participate in bacterial pathogenesis (64) were found in all six genomes. However, LRR genes were identified only in *Caldilineae* and SAR202 genomes (Fig. S4C and Table S5A). Many LRR proteins are involved in protein-ligand interactions; these interactions include plant immune response and the mammalian innate immune response (for a review, see reference 65), such as the detection of pathogen-associated molecular patterns by recognition receptors (66). Our findings are in good agreement with general patterns previously found in the metagenomes of sponge symbionts (38, 51), in enriched (mini)metagenomes of cyanobacterial sponge symbionts (59) and single amplified genomes from members of SAUL (20) and *Ca.* Poribacteria (58).

10.1128/mSystems.00150-18.9 TABLE S5 Absolute number and relative values of eukaryotic-like proteins (ELPs) (A) and of secondary metabolite gene clusters (B) identified in the analyzed genomes. Download Table S5, DOCX file, 0.01 MB. Copyright © 2018 Bayer et al. 2018 Bayer et al. This content is distributed under the terms of the Creative Commons Attribution 4.0 International license.

Another example of sponge-symbiont enriched features are the clustered, regularly interspaced, short, palindromic repeats (CRISPRs) and their associated proteins (Cas) that have recently been reported from the genomes of sponge symbionts (20, 38, 50, 51, 59). Here, the investigated *Caldilineae* and SAR202 genomes contained CRISPR-Cas systems, while the two most complete *Anaerolineae* genomes did not (Table 2). The presence of CRISPRs can be explained by the extensive filter-feeding activity of sponge hosts that result in high exposure of sponge symbionts to phages and other sources of free DNA from ambient seawater.

**TABLE 2:** Genomic characteristics of the six genomes investigated in detail^*a*^

Class/clade and SAG/bin^*b*^	Taxon ID	CRISPR		ANK	Secondary metabolite gene cluster		
		CRISPRfinder total (no. of repeats per spacer)	IMG total		Type 1 PKS	Terpene	Other
*Anaerolineae*							
SAG 1B	2617270794	-	-	1	-	-	-
A154*	2619619053	-	-	2	-	-	-
							
*Caldilineae*							
C141*	2619619051	7 (27, 24, 13, 7, 3, 30, 4)	9	9	2	-	-
C174*	2619619055	5 (21, 24, 8, 32, 27)	8	1	-	1	-
							
SAR202							
S152*	2619619052	5 (7, 34, 26, 7, 4)	9	5	3	4	1
S156*	2619619054	1 (13)	3	2	1	2	1

a The absolute numbers of CRISPR arrays defined by CRISPRfinder and IMG, the number of ankyrins and ankyrin repeat-containing proteins (ANK), as well as the number of secondary metabolite (antiSMASH) gene clusters per genome are shown. The IMG Gold Study IDs are Gs0114494 and Gs0099546. Secondary metabolite clusters were found using antiSMASH 3.0; the values are total numbers of genes per genome.

b Asterisks on bins indicate extracted metagenome bins from IMG Gold Study ID Gs0099546. The letters of the bins reflect the phylogenetic identity of the bin (A for *Anaerolineae*, C for *Caldilineae*, and S for SAR202).

The synthesis of secondary metabolites is an important defense mechanism of sessile organisms such as sponges to protect themselves against predators or biofouling (36). Many of these compounds are in fact produced by the sponge microbiome (10, 36). In particular, genes for polyketide synthases (PKS), nonribosomal peptide synthetases (NRPS), and halogenases are regularly enriched in sponge symbionts, often with new structures and putatively novel activities (51, 67–72). Here, we assessed the genomic repertoire of sponge-associated *Chloroflexi* for secondary metabolism using antiSMASH (73). In both SAR202 genomes and in *Caldilineae* C141, we found up to three polyketide synthase (PKS) gene clusters, all of which showed homologies to the previously reported type I PKS gene cluster from other sponge symbionts. Additional gene clusters for the production of terpenes and other yet to be identified substances were identified in the two SAR202 genomes and in *Caldilineae* C174 (Table 2 and Table S5B). Both *Anaerolineae* genomes did not contain any gene clusters for the biosynthesis of secondary metabolites. While the exact functions of these gene clusters putatively involved in defense remain unknown, it appears that at least SAR202 bacteria and *Caldilineae* have the genomic repertoire for chemical defense within the sponge holobiont.

### Ultrastructural identification of sponge-specific *Chloroflexi*.

**FIG 6:**
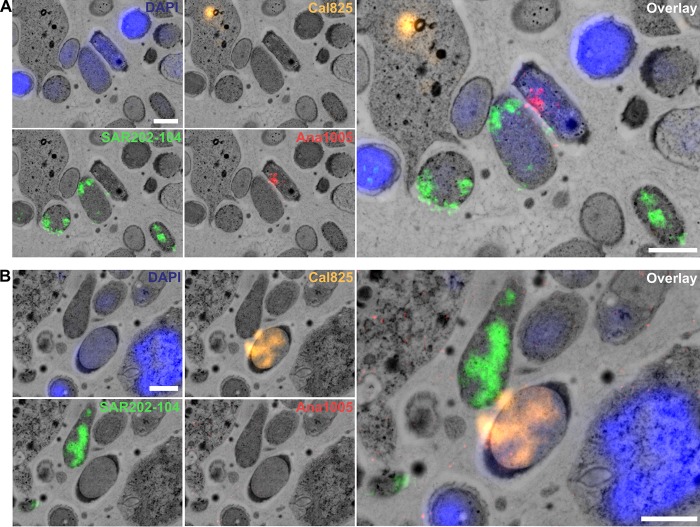
Visualization of sponge-associated *Chloroflexi* in *Aplysina aerophoba* mesohyl using FISH-CLEM. (A and B) SAR202 cells are displayed in green, *Anaerolineae* in red (A), and *Caldilineae* in orange (B). The nucleotide stain DAPI (blue) served as reference for the localization of unstained cells. In both panels, the picture on the right is the overlay of all probes and DAPI. Bars, 1 µm.

The correlation of probe-specific fluorescence with scanning electron microscopy (SEM) images allowed taxon-specific identification of *Chloroflexi* cells at ultrastructural resolution. Overall, distributions of all three *Chloroflexi* clades in *Aplysina aerophoba* indicate that SAR202 cells were more abundant than the other two *Chloroflexi* classes (Fig. 6). This is consistent with the relative abundances of *Chloroflexi* classes in HMA sponges extracted from EMP data (Fig. 1). Cells belonging to the SAR202 clade (green signals in Fig. 6A and B) are generally rod shaped (0.8 µm by 1 to 2 µm) with a regular distribution of cell cytosol content. The *Anaerolineae*-specific probe targeted rod-shaped cells (0.8 by 2.0 µm) (red signal in Fig. 6A). The characteristic feature of *Caldilineae*-positive cells (ca. 1  by 2 µm) was the presence of electron-dense capsules or mucus-like structures located at the cell poles (orange signal in Fig. 6B). All cells that were stained positive with a corresponding FISH probe showed a consistent morphology, which was taken as a measure of probe specificity.

### Conclusions.

Owing to the lack of cultivation and difficult experimental access for the majority of *Chloroflexi* clades, advancing knowledge has been limited to few lineages (22, 28, 74). The present study provides a new experimental opportunity, as HMA sponges were identified as true *Chloroflexi* hot spots, both in terms of biomass and biodiversity. Metagenomic and single-cell genomic analyses revealed metabolic specialization in that *Anaerolineae* and *Caldilineae* have an expanded gene repertoire for carbohydrate degradation. SAR202 genomes lack transporter and degradation pathways for carbohydrates, and we therefore speculate that they may gain the energy needed by degradation of amino and fatty acids. Similarly, while *Anaerolineae*/*Caldilineae* take up cofactors, SAR202 has the genomic repertoire for their synthesis. A combination of FISH-CLEM allowed us, for the first time, to visualize *Chloroflexi* in the host context and to identify characteristic cellular morphotypes. The results of this study suggest that *Chloroflexi* symbionts have the genomic potential for DOM degradation from seawater, both labile and recalcitrant. These findings are in line with previous reports that have shown extensive carbohydrate degradation potential in other HMA sponge symbionts (10, 19, 20). Thus, we hypothesize that collectively, sponge microbes not only provide nutrients to the HMA sponge host but also contribute to DOM cycling and primary productivity in reef ecosystems via a pathway termed the “sponge loop.” Considering the abundance and dominance of sponges in many benthic environments, we predict that the role of sponge symbionts in biogeochemical cycles is larger than previously thought.

## MATERIALS AND METHODS

### Relative abundance of *Chloroflexi* in high-microbial-abundance sponges.

To investigate the abundance of the bacterial phylum *Chloroflexi* on a global scale, microbiome data from HMA sponges, classified and predicted (cluster 1), were obtained from Moitinho-Silva et al. (31). This data set is a rarefied operational taxonomic unit (OTU) abundance matrix (23,455) from the mothur processed data of the Sponge Microbiome Project (31). The abundance of *Chloroflexi* OTUs was grouped according to the class level based on SILVA taxonomy (75). Relative abundances were calculated and displayed using the R packages ggplot2 version 3.0.0 (76) and ggpubr version 0.1.8 (https://CRAN.R-project.org/package=ggpubr). and complete-linkage hierarchical clustering was performed. For this purpose, Bray-Curtis dissimilarities were calculated on relative abundance values of *Chloroflexi* classes/clades within the phylum using the R package vegan version 2.4-5 (https://cran.r-project.org/package=vegan). To test the effect of geography and sponge phylogenetic on the beta diversity of *Chloroflexi* communities, Bray-Curtis dissimilarities were calculated on *Chloroflexi* abundances. Here, the rarified OTU abundance matrix that contained only *Chloroflexi* OTUs from classified and predicted HMA species was used. Samples with less than 100 sequences were excluded from the analysis. Type II permutation MANOVA using distance matrices was performed with RVAideMemoire package version 0.9-69-3 (https://CRAN.R-project.org/package=RVAideMemoire), using 999 permutations and an alpha level of 5%. For this test, each sample was assigned to the geographic region according to their collection sites. Sponge taxonomic order following NCBI taxonomy was used as a proxy of sponge phylogeny. Graphs and tests were performed in R environment version 3.4.3 (https://www.R-project.org/).

### Sponge sampling and cell separation and handling.

*Aplysina aerophoba* was collected from Piran, Slovenia (45.5099 N; 13.5600 E) in May 2013 and transported to the laboratory in Würzburg, Germany, in ambient seawater. Sponge-associated prokaryotes (SAPs) were enriched from fresh sponge tissues within 1 week of collection, for mesohyl and pinacoderm separately, by differential centrifugation as described previously (32). DNA was extracted from frozen SAP aliquots either from pinacoderm or mesohyl tissue (three replicates each) using the FastDNA SPIN kit for Soil (MP Biomedicals, Illkich, France) by the method of Slaby et al. (21). Briefly, different cell lysis protocols were applied for each triplicate to obtain differential sequencing coverage: (i) bead beating, following the manufacturer's protocol, (ii) freeze-thaw cycling (three cycles of 20 min at −80°C and 20 min at 42°C), (iii) proteinase K digestion for 1 h at 37°C (TE buffer with 0.5% SDS and proteinase K at a final concentration of 100 ng/ml). The quantity and quality of the extracted DNA were assessed by Nanodrop, Qubit high-sensitivity assay, and agarose gel electrophoresis. The DNA from two extraction rounds was pooled for each extraction approach separately, and the six sets of metagenomic DNA were sequenced on an Illumina HiSeq2000 platform (150-bp paired-end reads), quality filtered, and assembled at the DOE Joint Genome Institute (Walnut Creek, CA, USA) within the JGI sequencing and data processing pipeline (77). Differential coverage binning was performed with CONCOCT v. 0.2.1 (78) at default settings utilizing the coverage values from the six metagenomic data sets differing in tissue type and/or cell lysis method. A fasta file for each bin was created with the in-house python script mkBinFasta.py (https://github.com/bslaby/scripts/). Assembly statistics were obtained from QUAST v. 3.1 (79).

Single cells from cell preparations which were freshly prepared from a sponge from Rovinj/Croatia were sorted and their DNA was amplified by the method of Kamke et al. (19) and stored in 96-well plates at −80°C. Single amplified genomes (SAGs) were PCR screened using the universal primers 27f and 1492r to detect *Chloroflexi* 16S rRNA genes (67). SAGs that tested positive for the presence of a single *Chloroflexi* 16S rRNA gene were sequenced at GATC GmbH (Konstanz, Germany) on an Illumina MiSeq personal sequencer (300 bp; paired end). Sequences were trimmed with Trimmomatic-0.32 (minlen 150, avgqual 25, slidingwindow 4:25) (80) and filtered against eukaryotic, archaeal, and *Delftia* reads (known betaproteobacterial contaminant of the single-cell amplification kit) using blastn (nt database). The SAGs were assembled with SPAdes 3.5.0 (--sc, --careful, keep contigs >1000 bp) (81) decontaminated using the IMG/MER (Integrated microbial genomes & environmental samples) web tools following the single-cell data decontamination protocol provided at the JGI webpage (https://img.jgi.doe.gov/w/doc/SingleCellDataDecontamination.pdf). Only contigs showing clearly different GC/kmer frequency profiles from those of the bulk and that were not identified as *Chloroflexi* derived were filtered out. The SAGs were named according to the columns and rows of the 96-well plate they were identified.

### Phylogenetic tree construction.

16S rRNA genes from one metagenome bin (S165) and all 13 single amplified genomes (SAGs) were manually quality checked and aligned with closely related sponge- and non-sponge-derived environmental reference sequences obtained from the Silva database (SSU release 132) using the SINA aligner (82). The program MEGA 7.0.4 (83) was used to align the amino acid sequences of 60 genomes, in total 1,914 positions from nine ribosomal proteins (L2, L4, L14, L15, L22, L24, S3, S17, and S19). The determination of best tree construction model (JTT model), and final tree construction (neighbor-joining method) was conducted in MEGA. As references for the protein tree, sequences from publicly available genomes, basically from cultured *Chloroflexi* were included (see Table S1 in the supplemental material). Due to low genome completeness, some of the proteins used were missing in the relevant genomes. For the 16S rRNA gene-based tree, the neighbor-joining method (GTR+G+I model) was also applied using 1,787 positions from 182 sequences. The trees were visualized using iTOL (interactive tree of life; https://itol.embl.de/).

### Fluorescence *in situ* hybridization and FISH-CLEM.

FISH probes were designed based on the 16S rRNA gene alignment for sponge-specific clades within the classes *Anaerolineae* and *Caldilineae* using the probe design tool implemented in ARB (84). Candidate probes were tested *in silico* for their specific hybridization conditions using different target and nontarget reference sequences using mathFISH (http://mathfish.cee.wisc.edu/). The probes with the best performance were tested for hybridization specificity on fixed (4% paraformaldehyde) *A. aerophoba* microbial cell preparations by the method of Fieseler et al. (32) using formamide (FA) concentration gradients. Finally, we used for the *Caldilineae* probe Cal825 (5′-[Cy3]-ACACCGCCCACACCTCGT-[Cy3]-3′; *E. coli* binding positions, 825 to 843) and for the *Anaerolineae* probe Ana1005 (5′-[Alexa Fluor 647]-TCCGCTTTCGCTTCCGTA-[Alexa Fluor 647]-3′; *E. coli* binding positions, 1005 to 1023). Additionally, probe SAR202-104 (5′-[Alexa Fluor 488]-GTTACTCAGCCGTCTGCC-[Alexa Fluor 488]-3′; *E. coli* binding positions, 104 to 122) was used to identify members of the SAR202 group in sponges (85). All probes were double labeled at 5′ and 3′ ends (Sigma-Aldrich, Steinheim, Germany). To test the efficiencies of the newly designed sponge-specific *Chloroflexi* probes and the previously published SAR202-104R probe for the sponge microbiome, FISH conditions were optimized using microbial cell preparations from *A. aerophoba*. The three probes did not colocalize using 10%, 20%, and 30% FA, demonstrating specific binding of the probes to the *Chloroflexi* classes/clades in standard FISH experiments (Fig. S1).

For ultrastructural visualization of sponge *Chloroflexi*, we applied a recently established FISH-CLEM (fluorescence *in situ* hybridization-correlative light and electron microscopy) protocol (86). Briefly, freshly sampled *A. aerophoba* sponges were transported to the University of Wuerzburg where small mesohyl discs (2-mm diameter, 200-µm thickness) were subjected to high-pressure freezing (HPF) and freeze substitution. Samples were embedded in LR white, and 100-nm ultrathin sections were cut using a Histo Jumbo Diamond knife (Diatome AG, Biel, Switzerland) on a Leica EM UC7 ultramicrotome (Leica Microsystems, Wetzlar, Germany). The sections were placed on poly-l-lysine-coated slides and subjected to fluorescence *in situ* hybridization with the *Chloroflexi* clade-specific probes at 10% FA concentration (900 mM NaCl, 20 mM Tris-HCl [pH 7.4], 0.01% sodium dodecyl sulfate, 20% dextran sulfate). All three class- or clade-specific probes were cohybridized, and fluorescence signals were detected using an Axio Observer.Z1 microscope equipped with AxioCam 506 and Zen 2 version 2.0.0.0 software (Carl Zeiss Microscopy GmbH, Göttingen, Germany). On the same sections that were used for fluorescence microscopy, scanning electron microscopy (SEM) was carried out using a field emission scanning electron microscope JSM-7500F (JEOL, Japan) with LABE detector (for back scattered electron imaging at extremely low acceleration voltages) directly on the microscope slides. FISH and SEM images of same regions were computer correlated based on sponge heterochromatin pattern by the method of Jahn et al. (86).

### Functional genomic analysis.

Genomic data from SAG sequences and the extracted metagenome bins were loaded and analyzed in IMG (https://img.jgi.doe.gov/) using the KEGG Orthology (KO) terms assigned to our data sets, and metabolic pathways (KEGG) were analyzed. To identify CRISPR-related genes, CRISPRfinder (http://crispr.i2bc.paris-saclay.fr/Server/) was used. For the search of specific metabolite gene clusters, antiSMASH was used (73). The genomic potential of investigated microbial symbionts to degrade and transform complex carbohydrates was assessed by screening the IMG-predicted open reading frames (ORFs) of the genome data against the dbCAN (47) and classified according to the carbohydrate-active enzymes (CAZymes) database (48).

### Data availability.

Data sets for SAGs and the metagenomic bins are available at the NCBI Sequence Read Archive under the BioProject accession numbers or identifiers (IDs) PRJNA506133 and PRJNA366444 to PRJNA366449, respectively. Complete assembled and annotated data are available from IMG (https://img.jgi.doe.gov/) under the Gold Study IDs Gs0114494 and Gs0099546 (for more details, see Table 1).
