# Supplementary figures and images for: Marine Sponges as Chloroflexi Hot Spots: Genomic Insights and High-Resolution Visualization of an Abundant and Diverse Symbiotic Clade
Source: mSystems. 2018 Dec 26;3(6):e00150-18. doi: 10.1128/mSystems.00150-18 (PMC6306507; doi:10.1128/mSystems.00150-18)

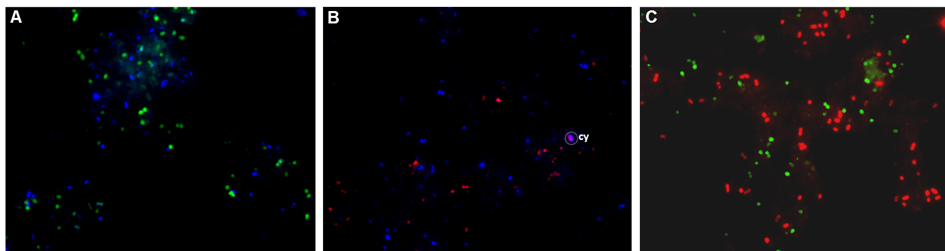

Supplement: FIG S1 [file sys006182305sf1.tif]

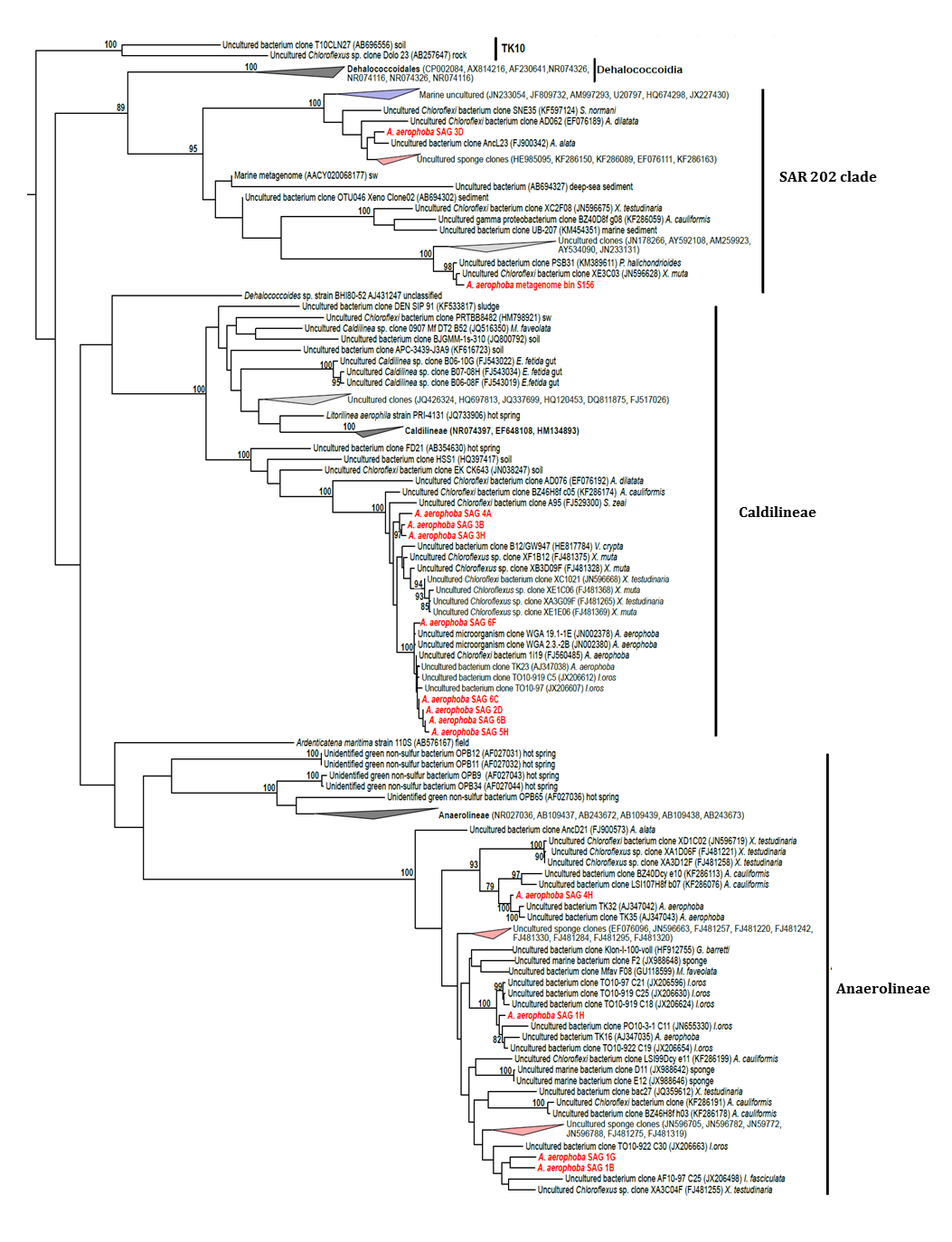

Supplement: FIG S2 [file sys006182305sf2.tif]

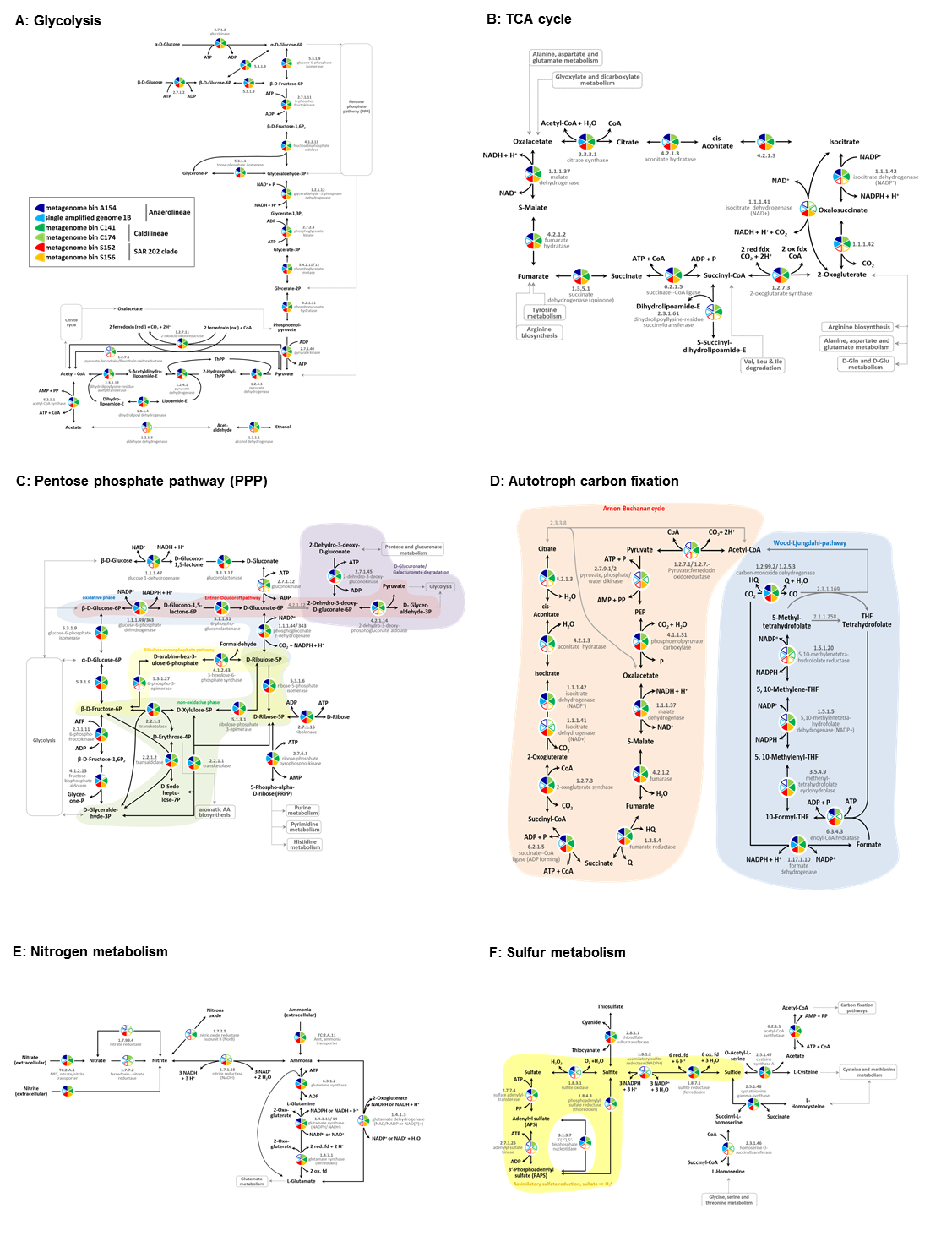

Supplement: FIG S3 [file sys006182305sf3.tif]

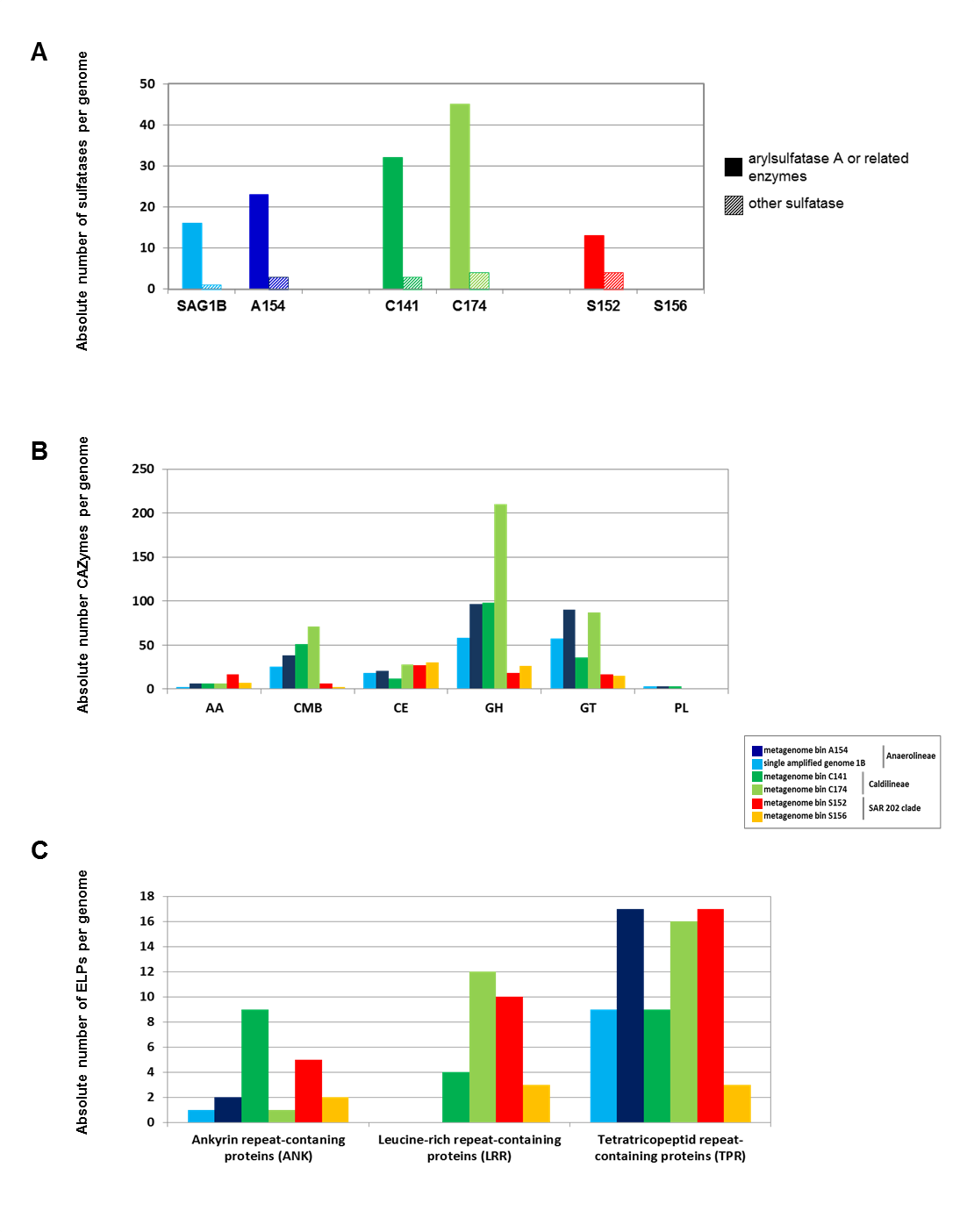

Supplement: FIG S4 [file sys006182305sf4.tif]
