# Supplementary material for: Marine Sponges as Chloroflexi Hot Spots: Genomic Insights and High-Resolution Visualization of an Abundant and Diverse Symbiotic Clade
Source: mSystems. 2018 Dec 26;3(6):e00150-18. doi: 10.1128/mSystems.00150-18 (PMC6306507; doi:10.1128/mSystems.00150-18)
Supplement: TABLE S1 [file sys006182305st1.docx]

Table S1: References genomes used for protein tree based on ribosomal genes

| **Class/ clade** | **Accession number** | **Name** |
| --- | --- | --- |
| Actinobacteria (outgroup) | NC_022115 | *Rhodococcus erythropolis* CCM2595 |
|  | AZAN01000000 | *Actinospica robiniae* DSM 44927 |
|  | JONS01000000 | *Actinomyces israelii* DSM 43320 |
| Dehalococcoida | CP000027 | *Dehalococcoides mccartyi* 195 |
|  | CP002084 | *Dehalogenimonas lykanthroporepellens* BL-DC-9 |
|  | CP001827 | *Dehalococcoides* sp. VS |
|  | CP006730 | *Dehalococcoides mccartyi* GY50 |
|  | CP001337 | *Dehalococcoides mccartyi* BAV1 |
|  | CP001924 | *Dehalococcoides mccartyi* GT |
|  | CP004079 | *Dehalococcoides mccartyi* DCMB5 |
| Ktedonobacteria | ADVG01000000 | *Ktedonobacter racemifer* DSM 44963 |
|  | JNIM00000000 | *Thermogemmatispora carboxidivorans* strain PM5 |
|  | Gs0033850 | Chloroflexi bacterium T81 |
| Thermobacteria | CP001825 | *Thermobaculum terrenum* YNP1 ATCC BAA-798 |
|  | CP001823 | *Sphaerobacter thermophilus* 4ac11 DSM 20745 |
|  | CAGS00000000 | *Nitrolancea hollandica* LB |
|  | CP001275 | *Thermomicrobium roseum* DSM 5159 |
|  | JQMP00000000 | *Thermorudis peleae* strain KI4 |
| Chloroflexi | LGKP00000000 | *Herpetosiphon geysericola* DSM 7119 |
|  | CP000875 | *Herpetosiphon aurantiacus* DSM 785 |
|  | PRJNA13622 | *Candidatus* Chlorothrix halophila |
|  | PRJNA195643 | *Roseiflexus castenholzii* DSM 13941 |
|  | CP000909 | *Chloroflexus aurantiacus* J-10-fl |
|  | CP001364 | *Chloroflexus* sp. Y-400-fl |
|  | AZXV01000000 | *Chloroflexus* sp. Y-396-1 |
|  | LJCR01000001 | *Kouleothrix aurantiaca* strain JCM 19913 |
|  | PRJNA16190 | *Roseiflexus* sp. RS-1 |
|  | NZ_ADVR00000000 | *Oscillochloris trichoides* DG6 |
|  | PRJNA195643 | Chloroflexi bacterium JGI 0001029-B04 |
|  | CP001337 | *Chloroflexus aggregans* DSM 9485 |
| Anaerolineae | LJNN00000000 | Anaerolineae bacterium SG8_19 |
|  | LGCM00000000 | *Levilinea saccharolytica* strain KIBI-1 |
|  | PRJNA194443 | Chloroflexi oral taxon 439 Chl1-2 |
|  | LGCK00000000 | *Leptolinea tardivitalis* strain YMTK-2 |
|  | BBXY00000000 | *Longilinea arvoryzae* strain KOME-1 |
|  | LGHJ00000000 | *Bellilinea caldifistulae* strain GOMI-1 |
|  | LGKO00000000 | *Thermanaerothrix daxensis* DSM 23592 |
|  | LGCL00000000 | *Ornatilinea apprima* P3M-1 |
|  | AP012029 | *Anaerolinea thermophila* UNI-1 |
|  | BBYH00000000 | *Flexilinea flocculi* strain TC1 |
|  | BBXW00000000 | *Anaerolinea thermolimosa* strain IMO-1 |
| **Class/ clade** | **Accession number** | **Name** |
| Caldilineae | AP012337 | *Caldilinea aerophila* STL-6-O1 DSM 14535 |
| Sar202 group | Gs0017605 | SAR202 cluster bacterium sp. SCGC AAA240-N13 |
| not clearly classified | PRJNA195829 | *Thermoflexus hugenholtzii* JAD2 |
| not clearly classified | BBZA00000000 | *Ardenticatena maritima* strain 110S |
|  | LDXM00000000 | Chloroflexi bacterium CSP1-4 |

Table S1, continued
