# Supplementary material for: Marine Sponges as Chloroflexi Hot Spots: Genomic Insights and High-Resolution Visualization of an Abundant and Diverse Symbiotic Clade
Source: mSystems. 2018 Dec 26;3(6):e00150-18. doi: 10.1128/mSystems.00150-18 (PMC6306507; doi:10.1128/mSystems.00150-18)
Supplement: TABLE S2 [file sys006182305st2.docx]

Table S2A: Sponge species used for EMP data set analysis, including indication of order ID, order and sample location as well as mean relative abundances (%) of the phylum *Chloroflexi* n HMA sponges (± standard deviations)

| **Sponge species** | **Order** | **Sample location** | **Mean** | **± Sd** |
| --- | --- | --- | --- | --- |
| *Agelas cervicornis* | Agelasida | Caribbean Sea | 10.64 | 8.05 |
| *Agelas conifera* | Agelasida | Caribbean Sea | 11.37 | 3.69 |
| *Agelas dispar* | Agelasida | Caribbean Sea | 12.43 | 4.42 |
| *Agelas oroides* | Agelasida | Mediterranean Sea | 19.81 | 7.80 |
| *Agelas* sp. | Agelasida | Caribbean Sea | 23.29 | 5.38 |
| *Aiolochroia crassa* | Verongiida | Caribbean Sea | 25.02 | 3.72 |
| *Aplysina aerophoba* | Verongiida | Mediterranean Sea | 23.76 | 5.73 |
| *Aplysina archeri* | Verongiida | Caribbean Sea | 20.07 | 3.83 |
| *Aplysina cauliformis* | Verongiida | Caribbean Sea | 17.06 | 4.69 |
| *Aplysina cauliformis* 'thin' | Verongiida | Caribbean Sea | 19.43 | 3.57 |
| *Aplysina cavernicola* | Verongiida | Mediterranean Sea | 21.78 | 4.86 |
| *Aplysina fistularis* | Verongiida | Caribbean Sea | 18.01 | 4.68 |
| *Aplysina fulva* | Verongiida | Caribbean Sea | 20.52 | 4.28 |
| *Aplysina* sp. | Verongiida | Caribbean Sea | 31.89 | 5.27 |
| *Biemna* sp. | Biemnida | Somali Coastal Current | 7.26 | 5.61 |
| *Cacospongia mollior* | Dictyoceratida | Mediterranean Sea | 16.18 | 1.69 |
| *Cacospongia scalaris* | Dictyoceratida | Mediterranean Sea | 21.18 | 1.41 |
| *Chondrilla australiensis* | Chondrillida | WC Australian Shelf | 6.25 | 1.36 |
| *Chondrilla caribensis* | Chondrillida | Caribbean Sea | 4.39 | 3.02 |
| *Chondrosia reniformis* | Chondrillida | Mediterranean Sea | 21.40 | 3.52 |
| *Cinachyrella alloclada* | Tetractinellida | Papua New Guinea | 11.47 | 5.49 |
| *Corticium candelabrum* | Plakinidae | Mediterranean Sea | 18.38 | 7.09 |
| *Corticium* sp. | Plakinidae | Mediterranean Sea | 27.23 | 2.64 |
| *Ectyoplasia ferox* | Axinellida | Caribbean Sea | 7.44 | 4.78 |
| *Erylus formosus* | Tetractinellida | Caribbean Sea | 20.35 | 5.21 |
| *Erylus* sp. | Tetractinellida | Caribbean Sea | 20.59 | 2.60 |
| *Geodia barretti* | Tetractinellida | North Sea | 19.61 | 8.25 |
| *Geodia* sp. | Tetractinellida | SE U.S. Continental Shelf | 18.27 | 13.03 |
| *Hippospongia* sp. | Dictyoceratida | WC Australian Shelf | 10.56 | 1.10 |
| *Hyrtios altum* | Dictyoceratida | Guam | 14.45 | 5.51 |
| *Hyrtios erectus* | Dictyoceratida | Red Sea | 20.66 | 3.70 |
| *Hyrtios proteus* | Dictyoceratida | Caribbean Sea | 26.37 | 5.44 |
| *Ircinia felix* | Dictyoceratida | Caribbean Sea | 12.26 | 8.59 |
| *Ircinia oros* | Dictyoceratida | Mediterranean Sea | 15.53 | 4.69 |
| *Ircinia* sp. | Dictyoceratida | NE Australian Shelf/ GBR | 8.57 | 3.51 |
| *Ircinia strobilina* | Dictyoceratida | Caribbean Sea | 29.46 | 13.65 |
| *Ircinia variabilis* | Dictyoceratida | Mediterranean Sea | 7.27 | 4.24 |
| **Sponge** | **Order** | **Sample location** | **Mean** | **± Sd** |
| *Luffariella* sp. | Dictyoceratida | Red Sea | 16.26 | 0.67 |
| *Neopetrosia proxima* | Haplosclerida | Caribbean Sea | 12.46 | 3.40 |
| *Neopetrosia* sp. | Haplosclerida | Caribbean Sea | 31.25 | 5.14 |
| *Neopetrosia subtriangularis* | Haplosclerida | Caribbean Sea | 21.87 | 12.99 |
| *Pachastrella* sp. | Tetractinellida | Caribbean Sea | 25.18 | 11.37 |
| *Petrosia ficiformis* | Haplosclerida | Mediterranean Sea | 18.60 | 5.01 |
| *Plakortis angulospiculatus* | Plakinidae | Caribbean Sea | 28.23 | 6.97 |
| *Plakortis halichondrioides* | Plakinidae | Caribbean Sea | 31.76 | 5.64 |
| *Plakortis simplex* | Plakinidae | South China Sea | 24.69 | 1.18 |
| *Plakortis* sp. | Plakinidae | Caribbean Sea | 30.04 | 10.77 |
| *Pseudoceratina sp.* | Verongiida | Guam | 18.35 | 5.44 |
| *Pseudocorticium jarrei* | Plakinidae | Mediterranean Sea | 22.65 | 2.19 |
| *Rhabdastrella globostellata* | Tetractinellida | Guam | 21.38 | 2.36 |
| *Rhaphoxya* sp. *2976* | Bubarida | Guam | 23.75 | 0.20 |
| *Rhopaloeides odorabile* | Dictyoceratida | NE Australian Shelf/ GBR | 18.01 | 7.52 |
| *Sarcotragus fasciculatus* | Dictyoceratida | Mediterranean Sea | 5.03 | 2.89 |
| *Sarcotragus* sp. | Dictyoceratida | Mediterranean Sea | 16.17 | 1.90 |
| *Sarcotragus spinosulus* | Dictyoceratida | Iberian Coastal | 14.72 | 3.56 |
| *Smenospongia aurea* | Dictyoceratida | Caribbean Sea | 15.96 | 8.20 |
| *Spongia agaricina* | Dictyoceratida | Mediterranean Sea | 19.22 | 3.29 |
| *Stelletta maori* | Tetractinellida | New Zealand Shelf | 10.41 | 9.69 |
| *Theonella swinhoei* | Tetractinellida | Red Sea | 18.92 | 2.84 |
| *Verongula rigida* | Verongiida | Caribbean Sea | 10.19 | 3.62 |
| *Xestospongia muta* | Haplosclerida | Caribbean Sea | 11.88 | 8.69 |
| *Xestospongia proxima* | Haplosclerida | Caribbean Sea | 25.72 | 4.06 |
| *Xestospongia testudinaria* | Haplosclerida | NE Australian Shelf/ GBR | 19.91 | 6.50 |

Table S2A, continued: NE: northeast, GBR: Great Barrier Reef, SE: Southeast, WC: West-Central.

Table S2B: Mean relative abundances (%) of *Chloroflexi* classes in HMA sponges (± standard deviations)

| **Class** | **Mean** | **± Sd** |
| --- | --- | --- |
| Anaerolineae | 11.6351102877306 | 12.2978991417359 |
| Ardenticatenia | 0.0433338227855173 | 0.176955300245546 |
| Caldilineae | 22.348929014365 | 17.9292359676175 |
| Chloroflexi_unclassified | 14.4950608850249 | 10.7679233772146 |
| Dehalococcoidia | 0.353881381350873 | 0.53022859614923 |
| Gitt-GS-136 | 1.21794698471647e-05 | 9.66715449486229e-05 |
| JG30-KF-CM66 | 0.0598689528547137 | 0.149307846331414 |
| KD4-96 | 0.00902476907275887 | 0.0570076507850635 |
| Ktedonobacteria | 0.0021825619439416 | 0.0166600198821925 |
| S085 | 0.835707552020923 | 0.80746794206041 |
| SAR202 clade | 47.7382798643836 | 22.0033838237772 |
| SHA-26 | 0.051934537800861 | 0.25092129488177 |
| Thermomicrobia | 0.000136823044413646 | 0.000641964374740606 |
| TK10 | 2.42653736815199 | 2.26380116902711 |

Table S2C: The effect of sponge geographic region and taxonomic order on *Chloroflexi* communities based on OTU abundances.

|  | **Sum Sq** | **Mean Sq** | **Df** | **F** | **p-value** |
| --- | --- | --- | --- | --- | --- |
| **Sample location** | 53.68424807 | 3.578949871 | 15 | 15.91651875 | 0.001 |
| **Taxonomic order** | 37.6494118 | 4.183267978 | 9 | 18.60407818 | 0.001 |
| **Residuals** | 163.0217447 | 0.224857579 | 725 | - | - |
| **Total** | 265.2698908 | - | 749 | - | - |

Type II permutation MANOVA analysis was performed with Bray–Curtis dissimilarities between sponge species obtained from OTUs abundances within the phylum. Sum Sq: sum of squares, Mean Sq: Mean Squares, Df: degrees of freedom
