# Supplementary material for: Marine Sponges as Chloroflexi Hot Spots: Genomic Insights and High-Resolution Visualization of an Abundant and Diverse Symbiotic Clade
Source: mSystems. 2018 Dec 26;3(6):e00150-18. doi: 10.1128/mSystems.00150-18 (PMC6306507; doi:10.1128/mSystems.00150-18)
Supplement: TEXT S1 [file sys006182305s1.pdf]

Marine sponges as Chloroflexi hot-spots: Genomic insights and high resolution visualization of an abundant and diverse symbiotic clade

Kristina Bayer, Martin T. Jahn, Beate M. Slaby, Lucas M. Silva, and Ute Hentschel

Supporting text – metabolic features

Detailed description of

- Central metabolism
- Sugar transport and metabolism
- Import and biosynthesis of co-factors and vitamins
- Amino sugar and nucleotide sugar metabolism
- Nucleotide metabolism
- Amino acid biosynthesis and metabolism (incl. degradation)
- Fatty acid metabolism
- Peptidoglycan biosynthesis
- Degradation of aromatic compounds

In all pathways displayed the filled pies compared to empty pies represent if the corresponding enzyme was identified or not. The colors represent the genomes. The numbers are KEGG identifiers. Grey arrows and not named enzymes indicate that enzyme was not identified.

Central metabolism

Metabolic reconstruction suggests that *Chloroflexi* are aerobic and heterotrophic bacteria (see supporting text and supplementary Fig.s for details). Genes involved in glycolysis and the tricarboxylic acid cycle (TCA) were almost completely identified in all metagenome bins (Fig. S2A, B). The pentose phosphate pathway (PPP), including the oxidative and non-oxidative phase is largely present. Also, the Entner-Doudoroff-pathway was identified, but lacks the gene encoding for enzyme phosphogluconate dehydratase (EC: 4.2.1.12) in all clades. Furthermore, the enzyme 2-dehydro-3-deoxyphosphogluconate aldolase (EC: 4.2.1.14) is missing in SAR202 (Fig. S2C). Interestingly, only the genomes of Anaerolineae and Caldilineae encode for enzymes involved in the ribulose monophosphate pathway (conversion of β-D-fructose-6P to D-ribulose-5P), which was originally found in methylotrophic bacteria but is now recognized as a widespread prokaryotic pathway involved in formaldehyde fixation and detoxification [1].

With respect to autotrophic carbon fixation, the reductive citrate acid cycle (Arnon-Buchanan cycle), is largely present, with the exception of ATP-citrate lyase (EC: 3.2.2.8), that is missing in all six genomes. A second pathway of autotrophic carbon fixation, the Wood-Ljungdahl-pathway was partially identified. While the genes encoding for carbon monoxide dehydrogenase (EC: 1.2.99.2) and formate dehydrogenase (EC: 1.2.1.43) are noticeably present in all six genomes, the rest of the Wood-Ljungdahl-pathway remains incomplete (see Fig. S2D). Ammonia import and assimilation is encoded on all investigated genomes, but SAR202 and Caldilineae have additional genes for glutamate synthesis from glutamine and directly from ammonia. The transport of nitrite (and possibly also nitrate) is encoded on all investigated genomes while the reduction to ammonia is encoded only by SAR202 (Fig. S2E). The incorporation of sulfur (with e.g. thiosulfate as donor) into S-containing amino acids might be possible in all clades whereas the assimilatory reduction of sulfate is restricted to Anaerolineae and Caldilineae genomes (Fig. S2F).

Genes encoding for enzymes of the respiratory chain, including succinate dehydrogenase, cytochrome c oxidase, NADH dehydrogenase and an f-type ATPase, are largely represented on all genomes. These energy gaining processes additionally provide precursors for further metabolic pathways such as biosynthesis of purines and pyrimidines, amino acids and co-factors, or structural compounds. Machinery for transcription and translation, purine and pyridimidine metabolism are largely present. Fatty acid (FA) biosynthesis and degradation pathways were detected in all six genomes. Genes involved in FA beta-oxidation were found almost completely (supporting text), but also the three key enzymes involved in the propionyl-CoA pathway for odd-length and methylated fatty acid degradation were found among the genomes. This includes propionyl-CoA carboxylase (EC:

6.1.4.3) which was annotated in all six genomes, methylmalonyl-CoA epimerase (EC 5.1.99.1), and methylmalonyl-CoA mutase (EC: 5.4.99.2) both of which were found in all genomes except in S152. All genomes encode a number of different ABC transporters to supplement for nutrition and cell growth related compounds (incl. oligopeptides, phosphate, L- and branched chain amino acids, minerals as iron (III) and molybdate, metal ions as zinc, manganese and iron (II)). Additionally, all six genomes largely encode enzymes needed for biosynthesis of most amino acids (see supporting text). We could not identify any of the typical phosphotransferase systems, as it was the case for *Ca.* Poribacteria described previously [2].

We found genomic potential for aromatic degradation in *Chloroflexi* genomes, but pathways remain incomplete (supporting text). Several genes encoding for phenylpropionate and cinnamate degradation, terephthalate degradation, catechol degradation, and xylene degradation were identified on *Chloroflexi* genomes. Also, genes encoding for enzymes involved in ring-cleavage by Baeyer-Villinger oxidation and beta oxidation as well as ring-hydroxylating dioxygenases and isomerases were identified which could be involved in degradation of aromatic compounds. This finding is interesting in the context that many sponge species contain secondary metabolites that serve as a defense strategy against predators and biofouling [3] and symbionts may be able of degradation of such substances as basis for life within sponge hosts . On the sponge genus level, highest (20-30% relative to the total microbiome) and most consistent presence of *Chloroflexi* within a sponge genus were found in the sponge genera Plakortis, Agelas (with the exception of *A. dispar*), *Aplysina* and sister taxon *Aiolochroia.* Interestingly, all of which contain characteristic natural products with aromatic ring structures that serve as chemotaxonomic markers (plakortolides, oroidins, bromo tyrosine alkaloids, respectively). It is therefore tempting to speculate that *Chloroflexi* and SAR202 presences and abundances are shaped, at least to some extent, by the natural products chemistry of their corresponding host sponges.

With respect to cell wall structure, the Anaerolineae and Caldilineae genomes encode the gene repertoire for peptidoglycan biosynthesis. The noticeable lack of peptidoglycan biosynthesis genes in the SAR202 genomes (supporting text) is consistent with previous analyses of three *Chloroflexi* genomes derived from uranium-contaminated aquifers [4]. Synthesis pathways encoding for lipopolysaccharides or biosynthesis pathways for other glycan-based membranes could also not be annotated. The synthesis of an S-layer was proposed for SAR202 bacteria [5] as well as a member of GIF09 clade of *Chloroflexi* [4] but genes involved in sialic acid formation (N-Acetylneuraminic acid - Neu5Ac) in the amino and nucleotide sugar metabolism pathway are incomplete (supporting text). Nevertheless, the SAR202 bin S152 encodes a type 2-ABC transporter (NodJI) to export lipo-oligosaccharides. These compounds were shown to play a role in nodulation process in rhizobium bacteria [6],

but their potential role for sponge-associated bacteria remains unclear. Additionally, consistent with previous observations [7] none of the six genomes encoded flagellar and chemotaxis genes.

Sugar transport and metabolism

The RbsBCA operon, encoding for an ABC transporter for Ribose and Xylose, was completely annotated in both Caldilineae genomes C141, C174, A154 and partially in SAG 1B. Ribose can enter the PPP upon conversion to ribose-5P by a ribokinase (EC: 2.7.1.15) and further to 5-phosphoribosyl diphosphate (PRPP) by a ribose-phosphate pyrophosphokinase (EC: 2.7.6.1) and can ultimately be used in purine and pyrimidine metabolism and histidine biosynthesis. Furthermore, ribose can be converted to D-gylceraldehyde-3P or D-glucose-6P (using reactions of the PPP), both of which can enter glycolysis. Ribose usage is encoded in all genomes (see below). Xylose can be converted to D-xyluolose-5P using the enzymes xylose isomerase (EC: 5.3.1.5) and xylulosekinase (EC: 2.7.1.17), which are fully present on Anaerolineae and Caldilineae genomes. D-Xylolose-5P can be converted by reactions of PPP, and again fuelling glycolysis.

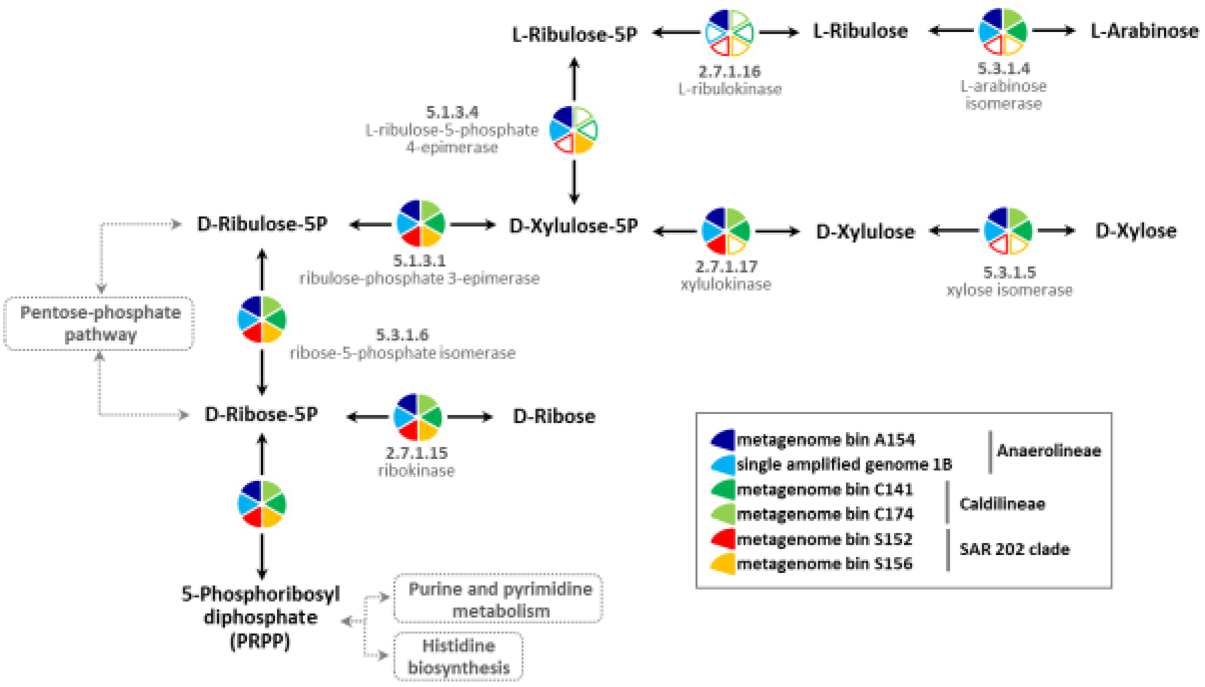

From the four genes coding for the rhamnose ABC transporter (RhaSPQT), three where identified in A154. L-Rhamnose can be converted to L-rhamnolose by the enzyme L-rhamnonate isomerase (EC: 5.3.1.14) and later to L-rhamnolose-1P by the L-rhamnonate

dehydratase (EC: 4.2.1.90). Both enzymes are annotated exclusively in Anaerolineae and Caldilineae genomes. L-Rhamnolose-1P can be split into lactate-aldehyde and glycerone-P, the later could fuel into glycolysis. However, the enzyme carrying that last step is missing in all genomes.

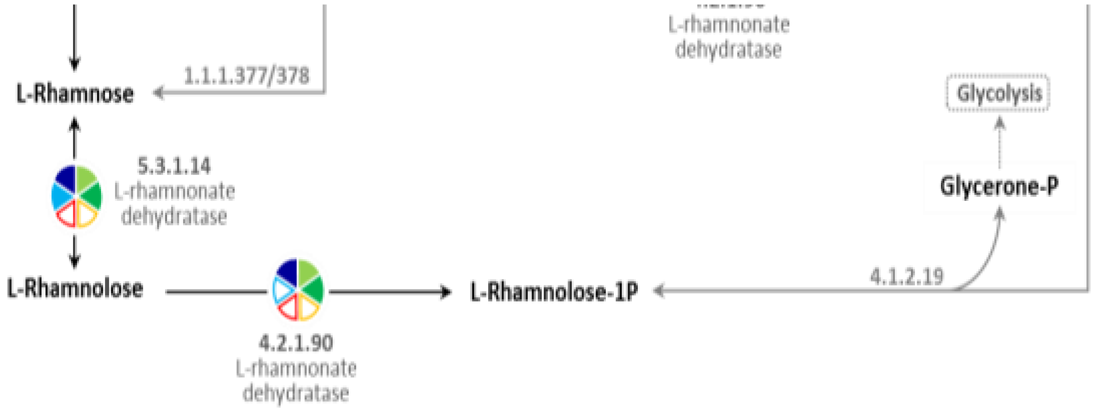

The *sn*-glycerol 3-phosphate (UgpBAEC) transporter was partially annotated in both Caldilineae and Anaerolineae genomes. The addition of two molecules acyl-CoA leads to the formation of fatty acids (glycerolipid metabolism). Although genomic potential to carry the necessary reactions was found completely only in bin C174, this could present a possible fatty acid biosynthesis pathway.

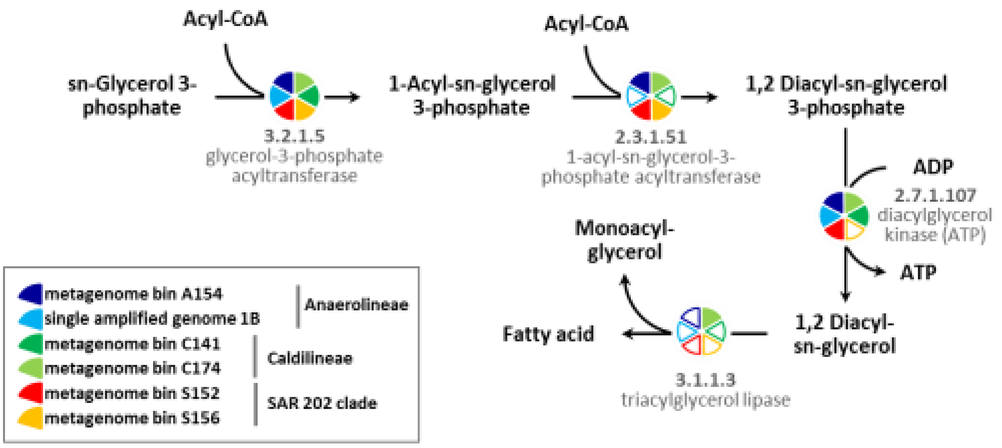

D-Fructose can be utilized to α-D-glucose by the enzyme xylose isomerase (EC: 5.3.1.5) which is encoded in both Anaerolineae and Caldilineae genomes, and to β-D-fructose-6P by

a fructokinase (EC: 2.7.1.4) which is encoded in all genomes except S156. The later one can be converted via D-mannose-6P (by mannose-6P isomerase, EC: 5.3.1.8) and D-mannose-1P (by a phosphomannomutase, EC: 5.4.2.8) to GDP-D-mannose which is utilized for N-glucan biosynthesis. GDP-D-mannose is also a precursor for the synthesis of other GDP-monodeoxyhexoses such as GDP-L-fucose and GDP-D-rhamnose which are used in lipopolysaccharide biosynthesis, but the enzymatic repertoire is limited to Anaerolineae genomes (for the enzyme GDP-L-fucose synthase, EC: 1.1.1.271) or Anaerolineae and Caldilineae (for the enzyme GDP-4-dehydro-6-deoxy D-mannose-reductase, EC: 1.1.1.281). Sorbitol and mannitol is possibly transported in cells by an ABC transporter (SmoEFGK) that was completely annotated in A154. D-sorbitol can be utilized using the enzyme L-iditol 2-dehydrogenase (EC: 1.1.1.14), which is encoded in all six genomes.

The ABC transporter MsmEFGK for the import of raffinose, stachyose and/ or melibiose was completely annotated in Anaerolineae and Caldilineae, but was absent in SAR202 genomes. The utilization of stachyose (to raffinose and galactose), raffinose (to sucrose), manninotriose (to melibiose and galactose), and melibiose to glucose and galactose is carried out by the enzyme α-galactosidase (EC: 3.2.1.22, GH31) and restricted to Anaerolineae and Caldilineae genomes. The β-fructofuranosidase (EC. 3.2.1.26, GH31) can convert raffinose to melibiose and stachyose to manninotriose and sucrose in D-glucose and D-fructose. The enzyme was annotated in all genomes except S156. Glucose can enter glycolysis - galactose utilization is described below.

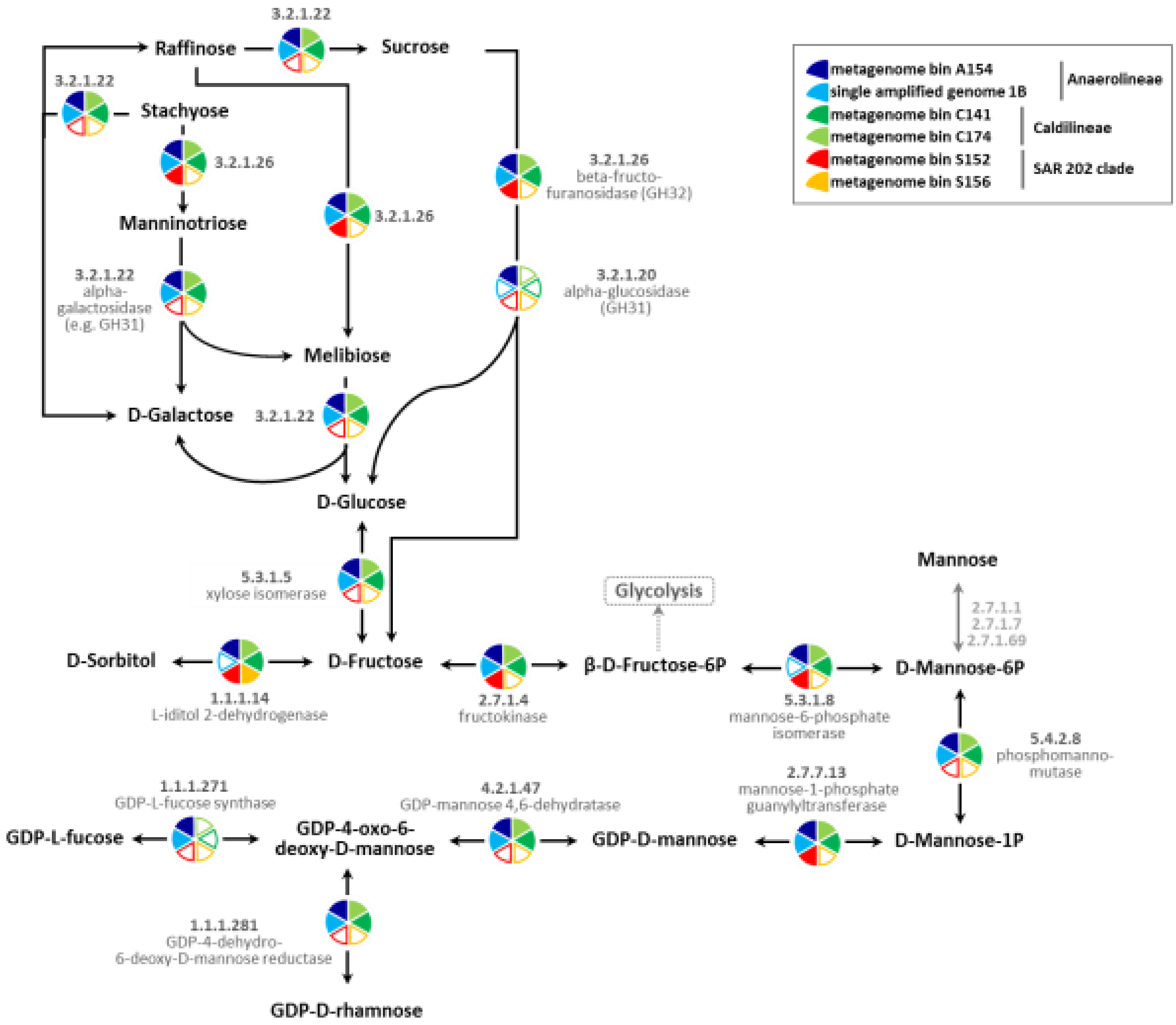

Galactose can be utilized to galactose-1P by the enzyme galactosidase (EC: 2.7.1.6) and to α-glucose-1P and UDP-glucose by the enzyme UDP glucose-hexose-1-phosphate uridylyltransferase (EC: 2.7.7.12). This degradation pathway (Leloir pathway) is encoded mainly in Anaerolineae and Caldilineae genomes. Additionally, the enzyme α-galactosidase (EC: 3.2.1.22) can break up α-D-galactosides, including galactose oligosaccharides, galactomannans and galactolipids to D-galactose and the remaining sugar alcohol. The utilization of inositol and sorbitol was described before, glycerol can be converted to sn-glycerol 3P (see above) by the enzyme glycerol kinase (EC: 2.7.1.3) which was annotated in all genomes except S156. Anaerolineae and Caldilineae genomes also encode for a galactose-ABC transporter (GanOPQ, MsmX) although the pathway was not complete. Alpha-glucose-1P can be converted to UDP-glucose by the enzyme UTP-glucose-1-phosphateuridylyltransferase (EC: 2.7.7.9) or to ADP-glucose by the enzyme glucose-1-phosphate adenylyltransferase (EC: 2.7.7.27), both can flow into amino sugar and nucleotide sugar metabolism as well as UDP-galactose.

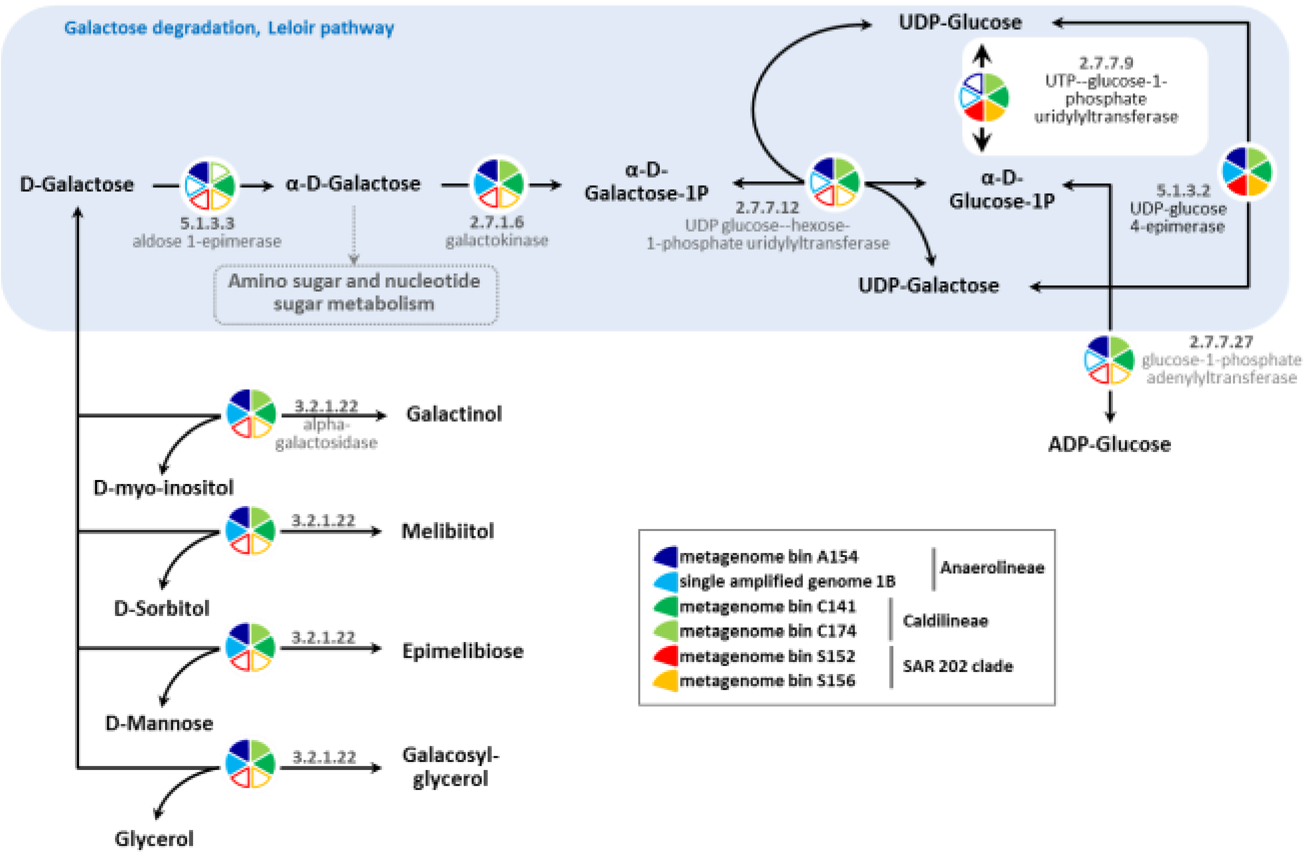

The enzyme beta-glucosidase (EC: 3.2.1.21, GH3), which is encoded in both Anaerolineae genomes and in C141 can convert glucosides in α-D-glucose and cellobiose and 1,4-β-D-glucan in β-D-glucose. An ABC transporter for α-glucosides (AglEFGK) was partially annotated, but only in SAG 1B. Alpha-glucosides need to be converted to glucose by the enzyme α-glucosidase (EC: 3.2.1.20, GH3) which is only encoded in genomes A154. The import and conversion of α-glucosides might be an Anaerolineae-specific feature.

The MalEFGK transporter (for maltose and/or maltodextrin aka amylose) was annotated in A154 only. Maltose can be converted α-D-glucose by the enzymes 4-α-glucanotransferase (EC: 2.4.1.25 - encoded in both Anaerolineae genomes) and α-glucosidase (EC 3.2.1.20 - only present in A154).

An incomplete transporter for trehalose/ maltose (ThuEFGK) was found in parts in Anaerolineae and Caldilineae genomes. The conversion of trehalose to trehalose-6P by the enzyme trehalose-6P phosphatase (EC: 3.1.3.12) and further to UDP-glucose and D-glucose-6P by the threhalose-6P synthase (EC: 2.4.1.15) was only encoded by both SAR202 genomes. Both resulting compound can be used in amino and nucleotide sugar metabolism; the later one can also enter glycolysis. Anaerolineae cells have the genetic potential to convert trehalose into starch using the Anaerolineae specific glycogen debranching enzyme

(EC: 3.2.1.196, GH13, CMB48). Starch might be converted further into amylose by the 1,4-α-glucan debranching enzyme (EC: 2.4.1.18, GH13, CMB48), which is encoded in both Anaerolineae and Caldilineae genomes.

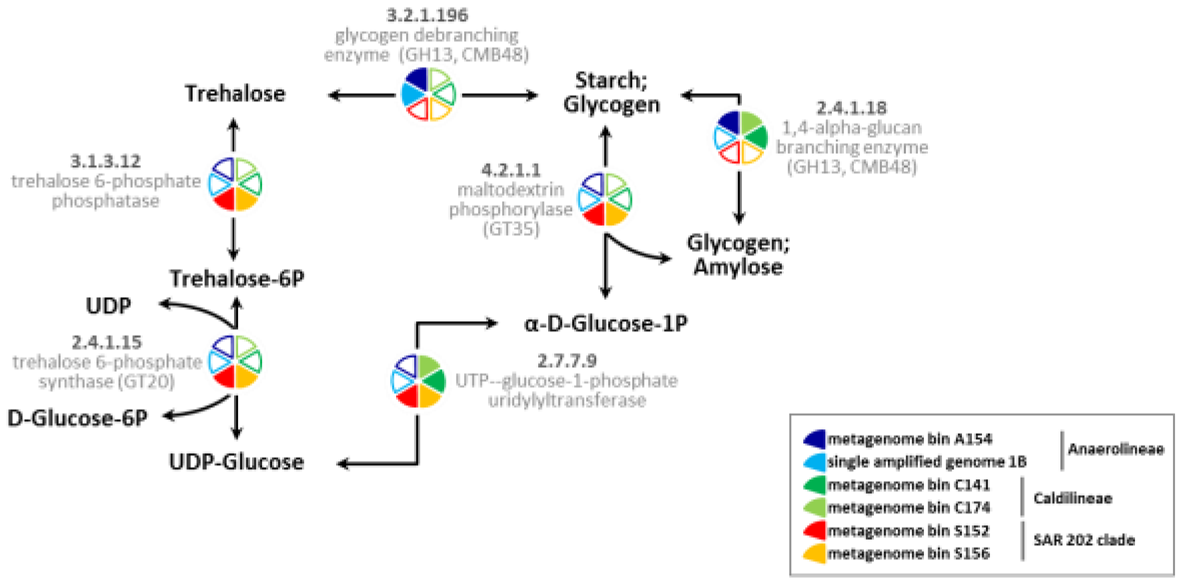

Almost all genomes encode for the genomic potential to oxidize β-D-glucose to D-gluconate by glucose 1-dehydrogenase (EC: 1.1.1.47) and gluconolactonase (EC: 3.1.1.17). While the first enzyme mentioned is missing in S152, the second was not annotated in S156. D-gluconate is further converted to D-gluconate-6P via gluconokinase (EC 2.7.1.12), which is only present in both Caldilineae genomes. The later one is then incorporated to the pentose phosphate pathway.

A largely complete ABC-transporter for aldouronate (LplABC) is annotated in both Caldilineae and Anaerolineae genomes but in the genomic context with peptide/ nickel transporter proteins. The required enzymes for the utilization of aldouronate (GH10: xylanases, GH43: β-xylosidase and GH67: α-glucuronidase) as described for *Paenibacillus* sp. Strain JDR-2 [8] are not annotated in the genomes. Interestingly, these glycosyl hydrolase classes were also not found in the dbCAN-based analysis of the genomes (suppl. table CAZy enzymes). However, aldouronates might result from hemicellulose degradation processes and could be accumulated by bacteria [8].

Enzymes involved in degradation of uronic acids were found in Anaerolineae and Caldilineae genomes. The possibility of galacturonate and glucuronate catabolism is supported by the conversion of 2-dehydro-3-deoxy-D-gluconate by enzymes glucoronate isomerase (EC:

5.3.1.12), tagaturonate reductase (EC: 1.1.1.58) and altronate hydrolase (EC: 4.2.1.7). Furthermore, the presence of genes encoding for oligogalacturonide lyase (EC: 4.2.2.6), 2-deoxy-D-gluconate 3-dehydrogenase (EC: 1.1.1.125) and 2-dehydro-3-desoxy-D-glucokinase (EC: 2.7.1.45) supports possible 4(4-α-D-gluc-4-enuronosyl)-D-galacturonate degradation activity. The products could then enter the ED pathway via 2-dehydro-3-desoxyphophogluconate aldolase (EC: 4.1.2.14). Uronic acid degradation could principally be connected to the inositol degradation pathway via D-galacturonate even though additional genome evidence, such as genes encoding for the enzyme inositol oxidase, (EC. 1.13.99.1) remain wanting.

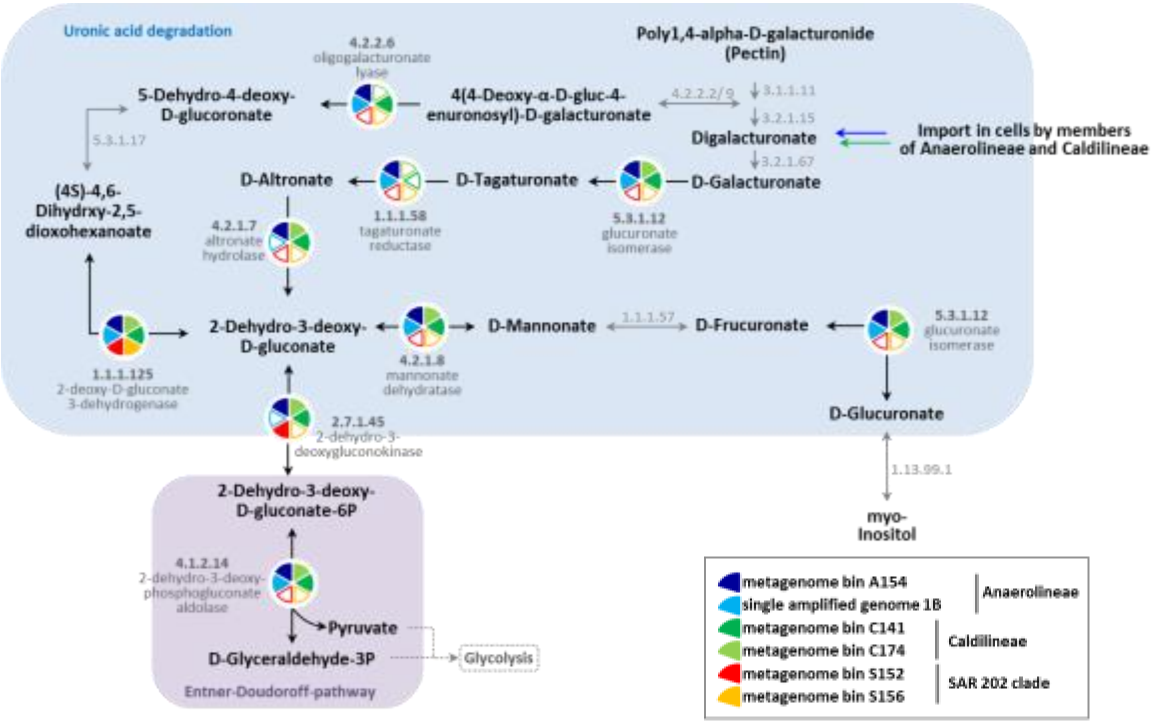

N-acetylglucosamine (GlcNAc)/ chitobiose could be imported by the NgcEFG encoded transporter which was annotated only in metagenome bin A154. GlcNAc can be used directly in amino sugar biosynthesis. This transporter also might transport chitobiose which is then converted to GlcNAc by the enzyme β-N-acetylhexosaminidase (EC: 3.2.1.52). However, the enzyme is encoded in both Anaerolineae and Caldilineae genomes but missing in SAR202 genomes.

The utilization of myo-inositol as carbon source and possibly as a regulatory agent was hypothesized previously for sponge-associated *Ca.* Poribacteria [2]. Similarly, sponge-associated Anaerolineae and Caldilineae encode the nearly complete inositol degradation pathway (Fig. 6). Myo-inositol is likely degraded to glyceraldehyde-3-phosphate and acetyl-CoA, which are further used in the central metabolism. Inositol phosphates are found as part

of eukaryotic and archaeal cell wall components [9]. Phosphorylated inositol is a precursor for several lipid molecules including sphingolipids, ceramides and glycosylphosphatidylinositol anchors [10], as well as many stress-protective solutes of eukaryotes [9] and might be part of the signal transduction in sponges [11]. Therefore the sponge itself or eukaryotic microorganisms can probably provide inositol as a carbon source or regulatory agent for the microbial symbionts.

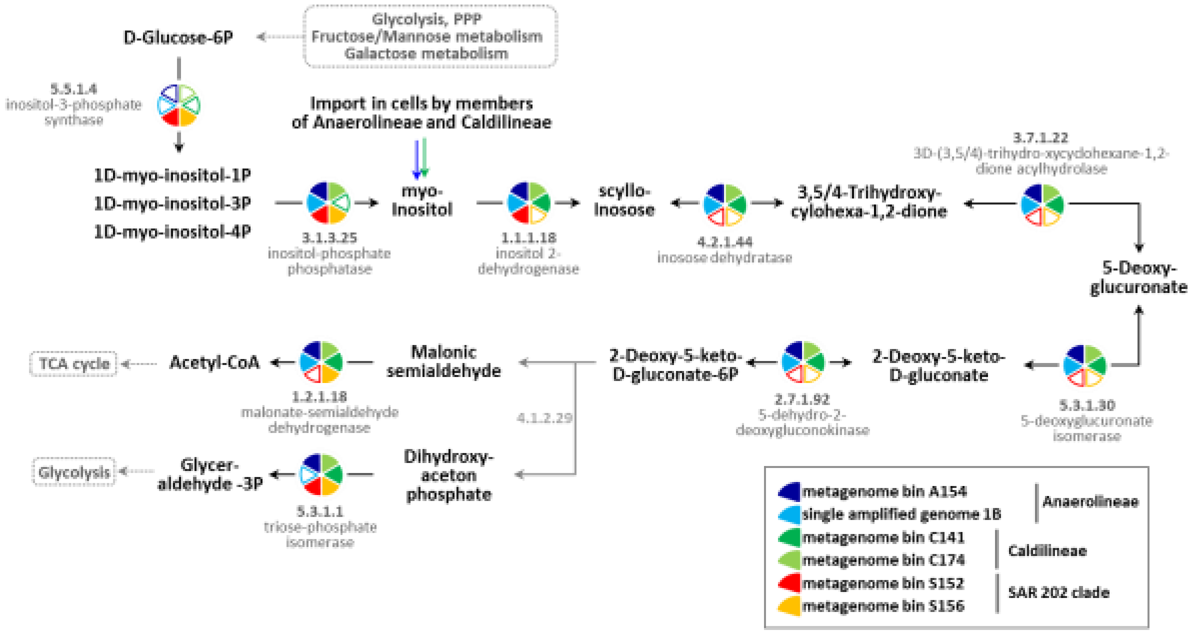

Import and biosynthesis of co-factors and vitamins

The biological role of polyamids as spermine, spermidine or putrescine ranges from basic ones as optimal cell growth, proliferation and biofilm formation to more specific ones as preventing phagolysis, bacteriocin production, toxin activity and protection from oxidative and acid stress (Shah and Swiatlo 2008; Michael 2016). Despite of above mentioned basic biological roles there is no conserved function of any polyamid in bacteria (Michael 2016). The ABC transporter for putrescine and spermindine (PotDCBA) was only present in Anaerolineae genomes. Interestingly, the biosynthesis of spermidine is most complete annotated in the genomes from SAR202 and could be synthesized from Arginine. In Anaerolineae and Caldilineae genomes the conversion from S-Adenosyl-L-methionine to S-Adenosylmethioninamine was not annotated at all and the final conversion of the later one and putescine to spermidine is annotated only in one Caldilineae genome (C141).

Alternatively, the conversion of agmatine to N-carbomoylprutescine (using the enzyme agmatine deaminase, EC: 3.5.3.12) and further to putrescine by the enzyme N-carbomoylprutescine amidase (EC: 3.5.1.53), might take place in Anaerolineae and Caldilineae, while Anaerolineae genomes encode additionally for a possible the import.

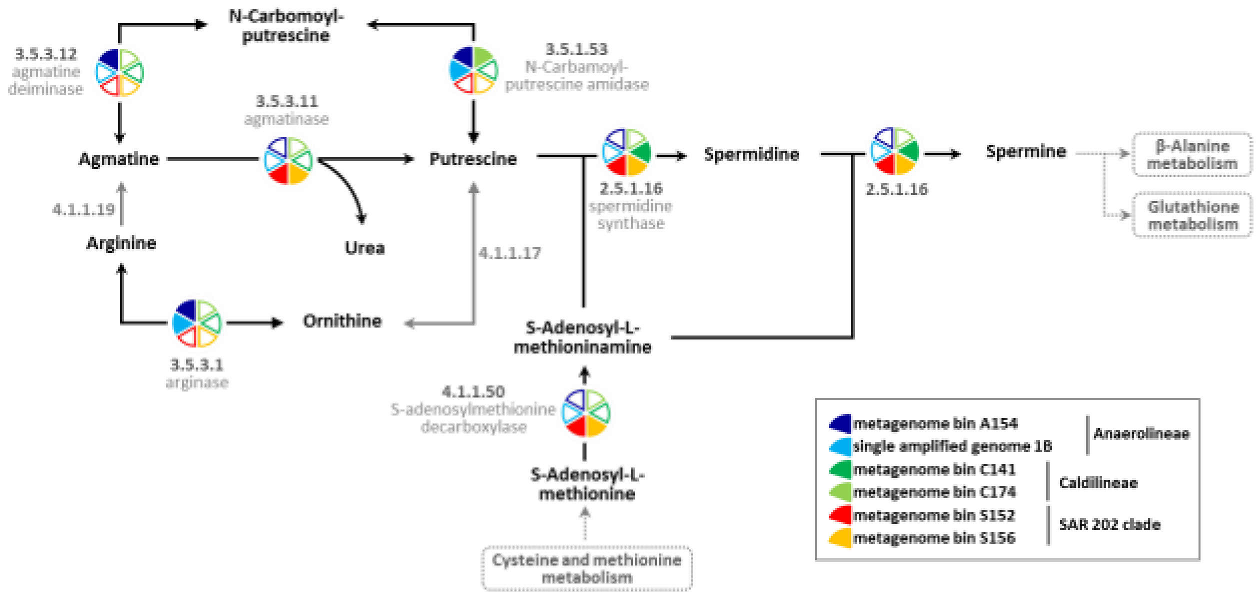

Lipoic acid is cofactor for at least five enzyme systems involved in glycolysis, TCA cycle, amino acid utilization and others. Lipoylated enzymes have lipoic acid attached to them covalently. The lipoyl group transfers acyl groups in 2-oxoacid dehydrogenase complexes, and methylamine group in the glycine cleavage complex or glycine dehydrogenase. The salvage pathway is mainly encoded by Anaerolineae and Caldilineae genomes, whereas LipB (EC: 2.3.1.181) is encoded only in SAR202 genomes.

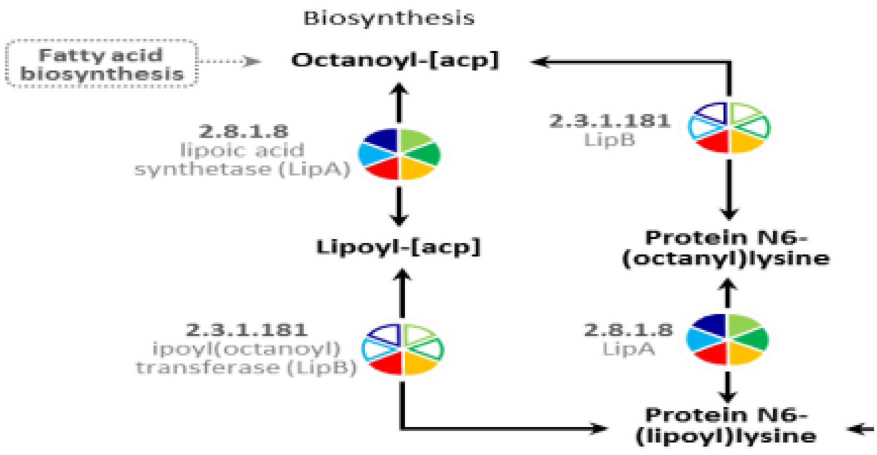

Nicotinic acid (anionic form: nicotinate) is also known as niacin or vitamin B3. Nicotinamide is the amide derivative of nicotinic acid. Nicotinate and nicotinamide are essential for organisms as the precursors for generation of coenzymes, NAD^+ and $NADP^+$, which are essential for redox reactions and carry electrons from one reaction to another. They therefore exist in oxidized ($NAD(P)^+$) and reduced (NAD(P)H) forms. These coenzymes are crucial for many metabolic pathways including glycolysis, TCA cycle, pentose phosphate cycle, fatty acid biosynthesis and metabolism pathways and many others.

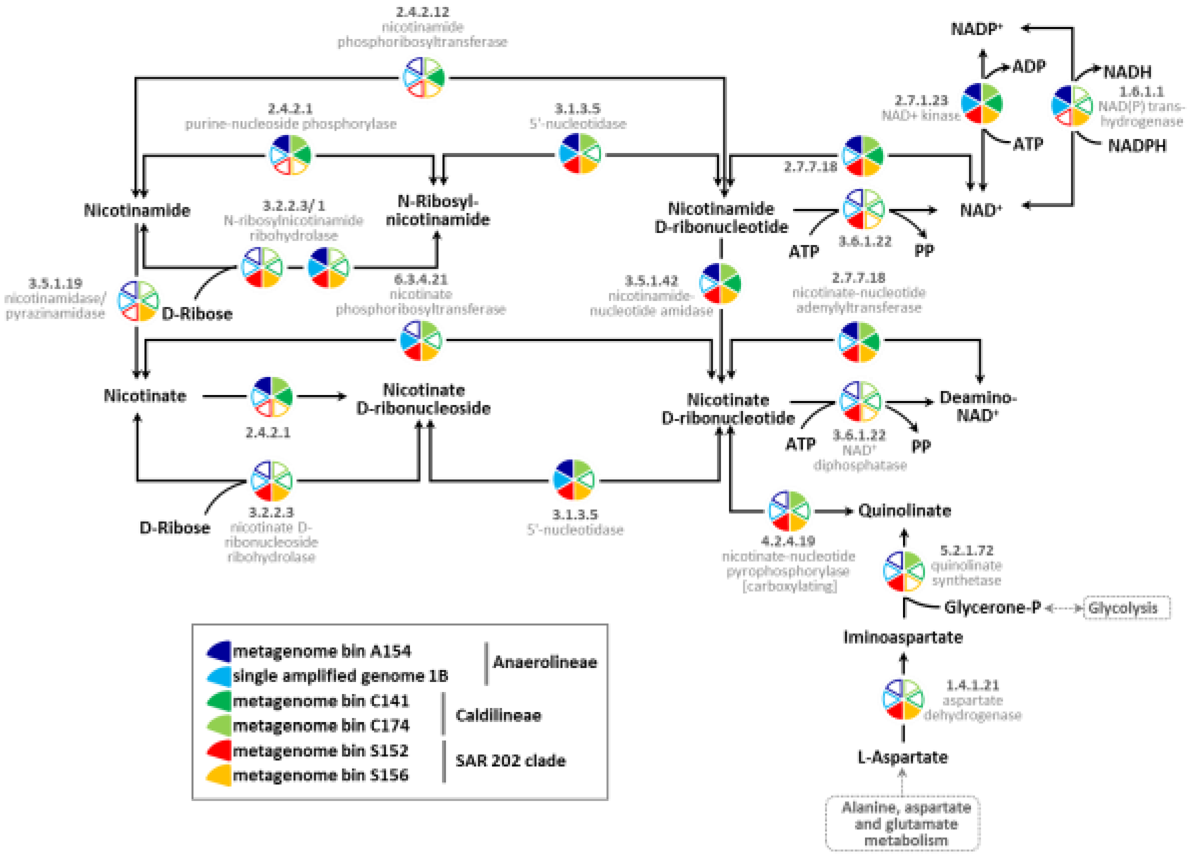

Pantothenic acid (Pantothenate) is used in the synthesis of coenzyme A (CoA). Coenzyme A may act as an acyl group carrier to form acetyl-CoA and other related compounds. CoA is important in energy metabolism for pyruvate to enter the tricarboxylic acid cycle (TCA cycle) as acetyl-CoA, and for α-ketoglutarate to be transformed to succinyl-CoA in the cycle. CoA is also important in the biosynthesis of many important compounds such as fatty acids, cholesterol, and acetylcholine. CoA is incidentally also required in the formation of ACP, which is involved in fatty acid synthesis in addition to CoA. Pantothenic acid in the form of CoA is also required for acylation and acetylation, which, for example, are involved in signal transduction and enzyme activation and deactivation, respectively. Since pantothenic acid

participates in a wide array of key biological roles, it is essential to all forms of life. While most conversions in the biosynthesis of CoA are encoded in all genomes, some enzymes (as the ketopantonate hydroxymethyltransferase, EC: 2.1.2.11 and the pantothenate synthase, EC: 6.3.2.1) seem to be restricted to SAR202 group members. The same is true for the enzyme phosphopanthetheinyl-transferase (EC: 2.7.8.-) which is a member of a superfamily of essential enzymes required for the synthesis of a wide range of compounds including fatty acid, polyketide, and nonribosomal peptide metabolites. These enzymes activate carrier proteins in specific biosynthetic pathways by the transfer of a phosphopantetheinyl moiety to an invariant serine residue (see scheme below).

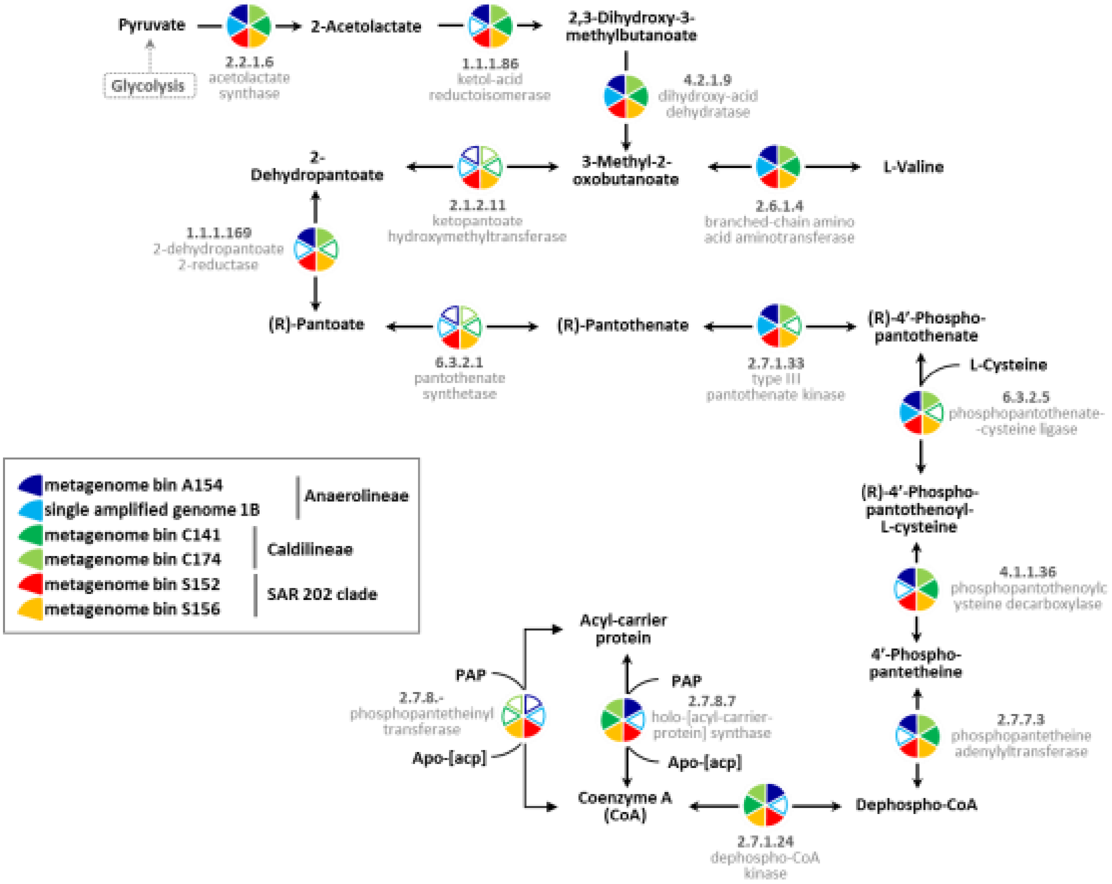

All cells, both prokaryotic and eukaryotic, require reduced folate (vitamin B9) cofactors for the biosynthesis of a diverse range of cellular components. Tetrahydrofolate serves as a donor of one-carbon units in a variety of biosynthetic processes, including the formation of methionine, purines and thymine. Furthermore, tetrahydrofolate can also act as an acceptor of one-carbon units (see below) in degradative reactions. While the cellular requirement for folates is universal, methods for obtaining them differ between prokaryotes and eukaryotes. Whereas mammals possess an active transport system, utilising membrane-associated folate transport proteins, in most microorganisms folates must be synthesised de novo

through the folate biosynthetic pathway. Largely, the synthesis up to DHF (an intermediate in the synthesis to folate) is encoded in all three Chloroflexi classes/ groups. The synthesis of 7,8-Dihydroneopterin 3'-phosphate from GTP is encoded only in Anaerolineae and SAR202 genomes, and the final conversion to folate by the enzyme dihydrofolate reductase (EC: 1.5.1.3) is missing in all genomes.

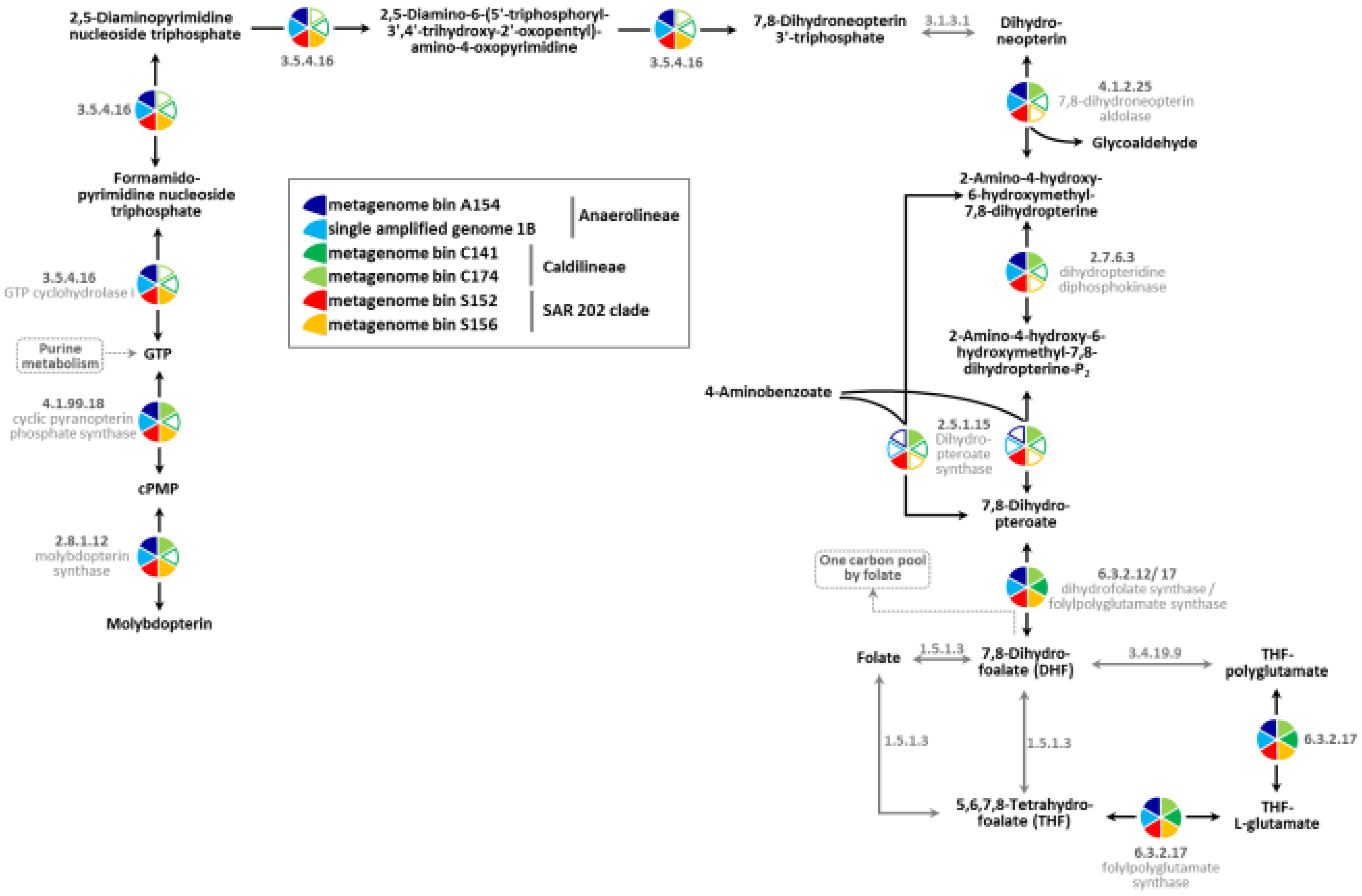

There is a group of biochemical reactions which are involved in amino acid metabolism and also play roles in nucleotide metabolism, that have a special set of enzymes and coenzymes. These reactions are referred as one-carbon metabolism since the transfer of one-carbon groups which are too volatile and need to be attached to something while being processed is in common. The compounds 5-formyl-THF and 5,10-methenyl-THF are of special interest since these are used in purine ring formation. The important formation of 5,10-methenyl-THF from THF is carried out by different enzyme and all analyzed Chloroflexi genomes encode for this function. The conversion of THF to 5-formyl-THF or 5-formimino-THF by the enzyme glutamate formiminotransferase (EC: 2.1.2.5) is encoded only in Cladilineae genomes. The enzyme formiminotetrahydrofolate cyclodeaminase (EC: 4.3.1.4) is annotated only in

Caldilineae genomes. The formation of 5,10-methenyl-THF by an dehydrogenase (EC: 3.5.4.9) and further to 5,10-methylene-THF by a cyclohydrolase (EC: 1.5.1.5) is encoded only in Caldilineae and SAR202 genomes. The enzyme phosphoribosylglycerinamide formyltransferase (EC: 2.1.2.2) which converts THF to 10-formyl-THF and 5,10-methenyl-THF is restricted also to Caldilineae and SAR202 genomes.

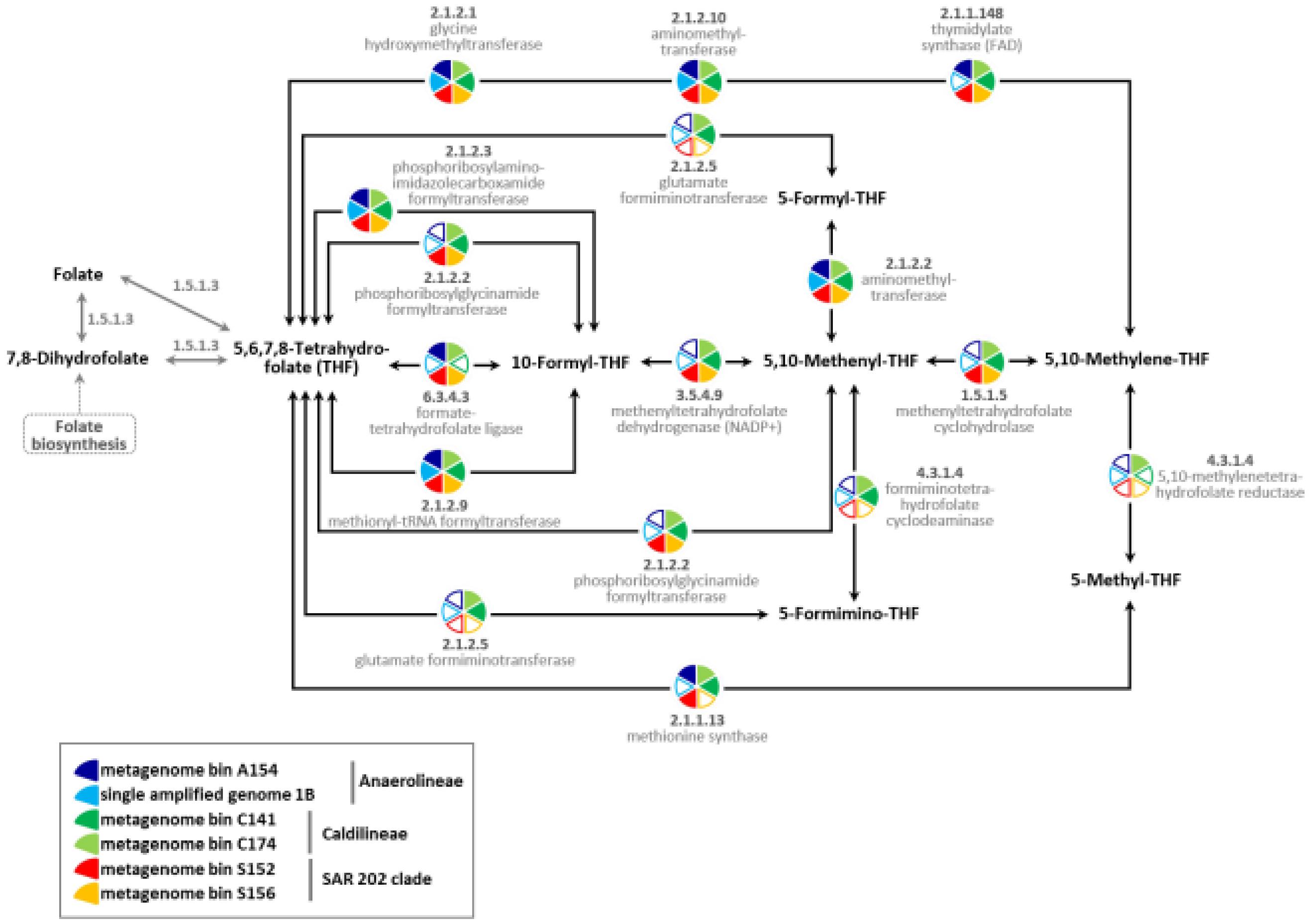

Sponge *Chloroflexi* genomes encode several genes in tetrapyrrole formation (porphyrin synthesis) pathways. The synthesis of protoheme from L-glutamate is largely encoded whereas it is not complete in all genomes. However, the further conversion to heme A, which is involved in the formation of cytrochrome c oxidase, is annotated only in Caldilineae and SAR202 genomes. The synthesis of a vitamin B12 precursor Cob(II)yrinate a,c diamide coming from the siroheme pathway is largely encoded but mainly in S152. The synthesis of vitamin B12 from riboflavin seems to be restricted to SAR202 genomes. The L-threonine path leading into vit B12 synthesis is encoded only partially in single genomes.

Ubiquinone (UQ), also called coenzyme Q, and plastoquinone (PQ) are electron carriers in oxidative phosphorylation and photosynthesis, respectively. The quinoid nucleus of ubiquinone is derived from the shikimate pathway; 4-hydroxybenzoate is directly formed from chorismate in bacteria. The following biosynthesis of terpenoid moiety involves reactions of prenylation, decarboxylation, and three hydroxylations alternating with three methylations. Menaquinone (vitamin K2) is an obligatory component of the electron-transfer pathway in bacteria. Interestingly, the main biosynthesis pathway from chorismate to 2-succinylbenzoyl-CoA is mainly encoded by Anaerolineae and Caldilineae genomes but is missing in SAR202 genomes.

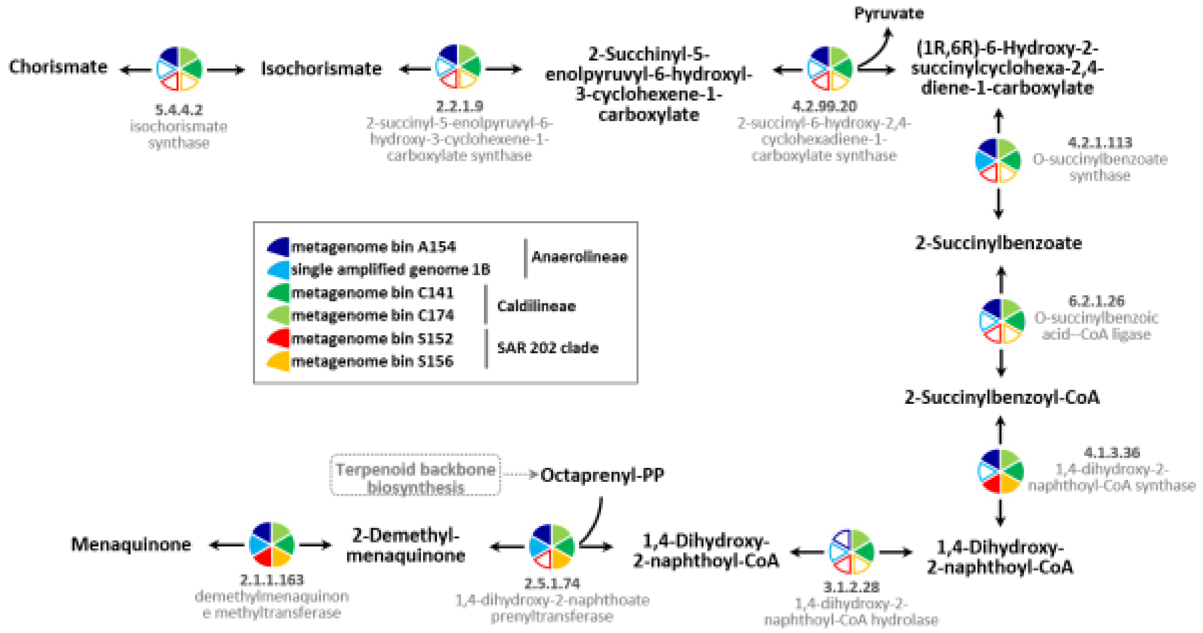

Biotin (coenzyme R, vitamin H or vitamin B7) is the essential cofactor of biotin-dependent carboxylases, such as pyruvate carboxylase and acetyl-CoA carboxylase. Bacteria can synthesize biotin from pimelate thioester through different pathways: However, none of these known biotin biosynthesis pathways is encoded (or annotated) in the sponge *Chloroflexi* genomes. But, BioY from the biotin ABC transporter (BioYNM) is encoded by both SAR202 genomes and therefore biotin might be imported instead of being synthesized at least by SAR202 *Chloroflexi*. Pyridoxine (vitamin B6) and vitamin A (retinol) biosynthesis genes are not encoded in any of the genomes.

Amino sugar and nucleotide sugar metabolism

All genomes encode for enzyme providing sugars for synthesis of amino acids and nuceotides. Also here we could see some phylogenetic specialization in genome analysis. As described before the conversion of chitobiose into N-acetylglucosamine (GlcNAc), and further conversion in N-acetylglucosamine-6P (GlcN-6P) is restricted to Anaerolineae and Caldinlineae genomes. The same was found for following conversions: UDP-N-acetylglucosamine (UDP-GlcNAc) in UDP-N-acetylmuramic acid (UDP-MurNAc) or UDP-N-acetyl-D-glucosaminuronate (UDP-GlcAcA), UDP-glucose in UDP-D-glucuronate (UDP-GluA) or UDP-6-sulfoquinovose (UDP-SQ), galactose in UDP-galactose, and mannose-6P in GTP-rhamnose. Interestingly, the missing synthesis in Sar202 genomes might be compansate by a nucleobase/ H^+ symporter annotaed in both genomes in multiple copies.

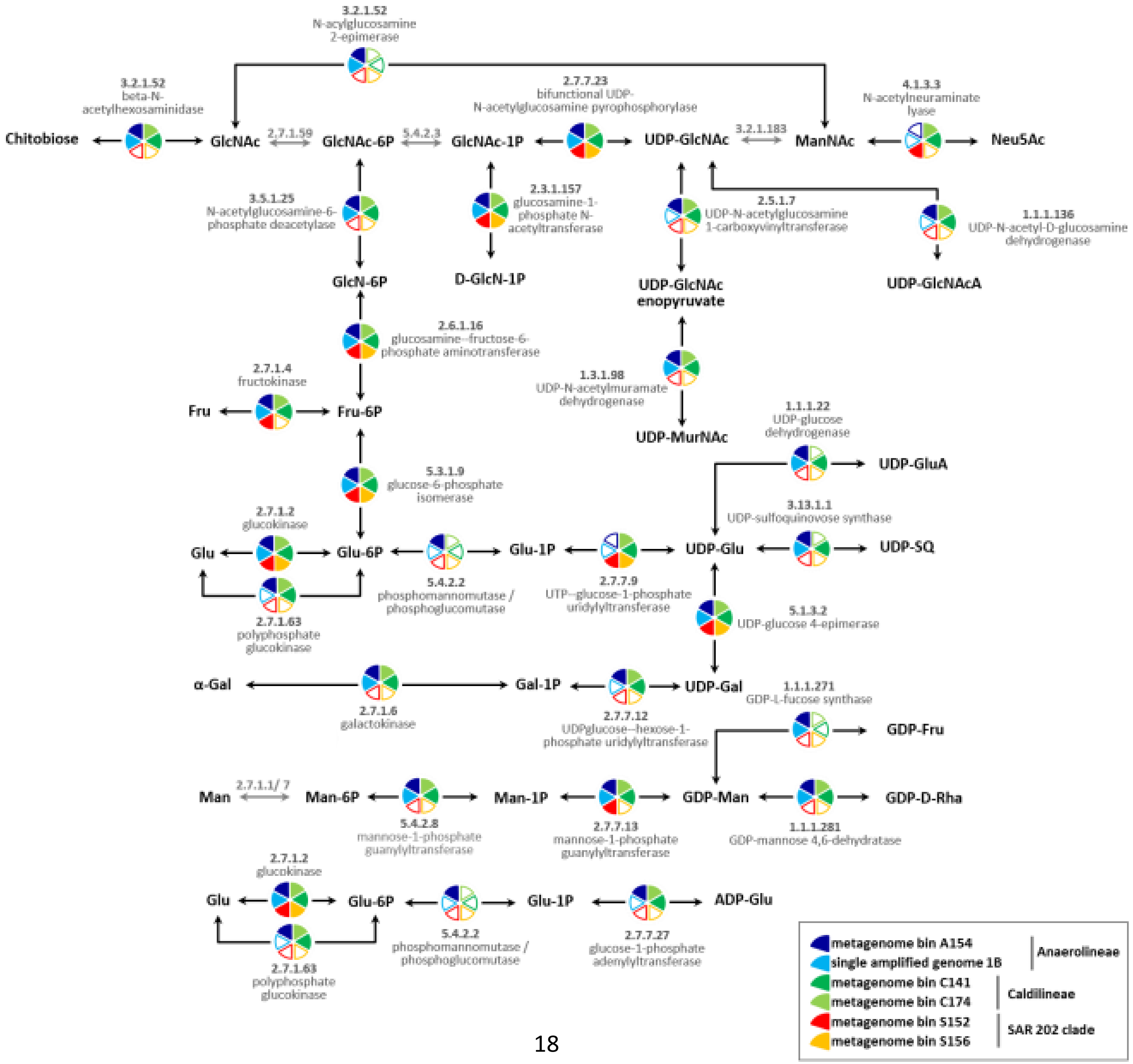

Nucleotide metabolism

The synthesis from Ribose-5P to PRPP is encoded in all six genomes, whereas the further enzymatic conversion to GAR, FGAR, FGAM, AIR, CAIR, SIACAR to AICAR was present only in Caldilineae and SAR202 genomes. But, AICAR results as by-product in histidine biosynthesis and Anaerolineae might compensate missing pathway described above by using this. AICAR is then converted to iosinine-monophosphate (IMP) as precursor for other purine derivates via FAICAR which was encoded in all genomes. The synthesis of adenosine and guanine (including all deoxy derivatives) all genomes have in common. The synthesis of UMP from L-glutamine was annotated completely in all six genomes. The further formation of CTP, and UTP as well as the synthesis of TTP from thymidine is basically encoded in all genomes whereas there might be enzymatically specialization especially in Caldilineae and SAR202 genomes. The final formation of DNA by the DNA-directed DNA polymerase (EC: 2.7.7.7) and RNA by the DNA-directed RNA polymerase (EC: 2.7.7.6) is encoded in all genomes.

Amino acid biosynthesis and metabolism

The analysis of the six genomes of sponge associated *Chloroflexi* reflect that they all have the genetic potential to synthesize most amino acids by themselves. Serine, glycine, threonine, aspartate, cysteine, leucine isoleucine and valine can be synthesized from pyruvate (see below). The enzymes involved are encoded in almost all genomes except SAG1B, presumably due to its incompleteness. L-Alanine can be synthesised directly from pyruvate by all *Chloroflexi* whereas the further conversion to D-alanine, which occurs in polypeptides in some bacterial cell walls and in some peptide antibiotics, seems restricted to Anaerolineae and Caldilineae.

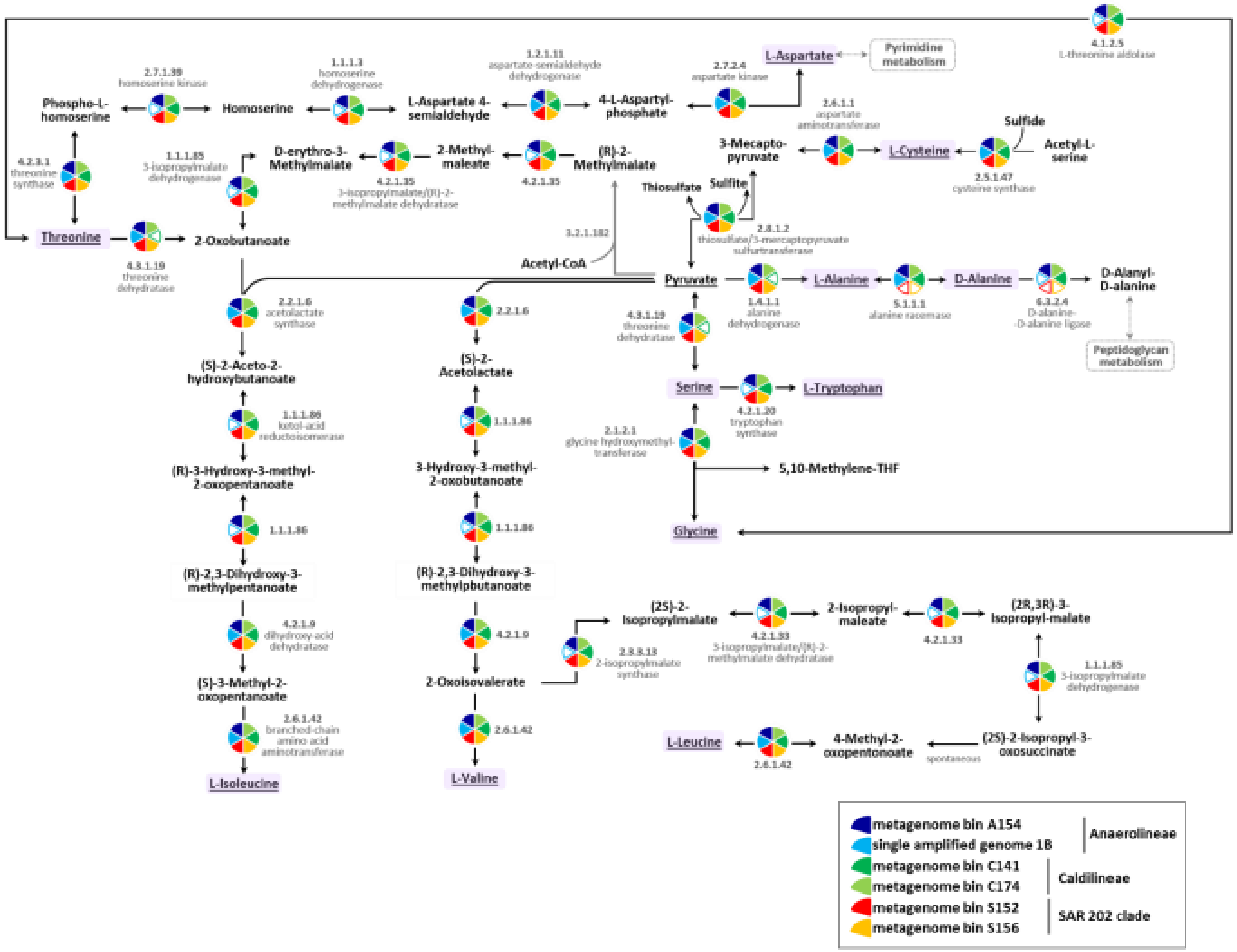

All sponge Chloroflexi cells are able to synthesize aspartate alternatively from products of the TCA cycle (fumarate, 2-oxogluterate). Glutamine can be synthesized using the enzyme glutamine synthetase (EC: 6.3.1.2) which is encoded by all six genomes whereas glutamate synthesis from NH_3 and 2-oxogluterate by the enzyme glutamate dehydrogenase (EC: 1.4.1.3) was annotated only in Caldilineae and one SAR202 genome. Alternatively, glutamate can be synthesized from glutamine via carbamoylphosphate synthase (EC: 6.3.5.5). Proline is synthesized from L-glutamate by all three clades, whereas the enzyme for the synthesis from peptides (proline phosphate synthase, EC: 3.4.11.5) is encoded only in Caldilineae genomes. All genomes encode for the enzyme alanine dehydrogenase (EC: 4.3.1.12) which is able to convert proline into ornithine, while synthesis from arginine by the enzyme arginase (EC: 3.5.3.1) is encoded only in Anaerolineae genomes.

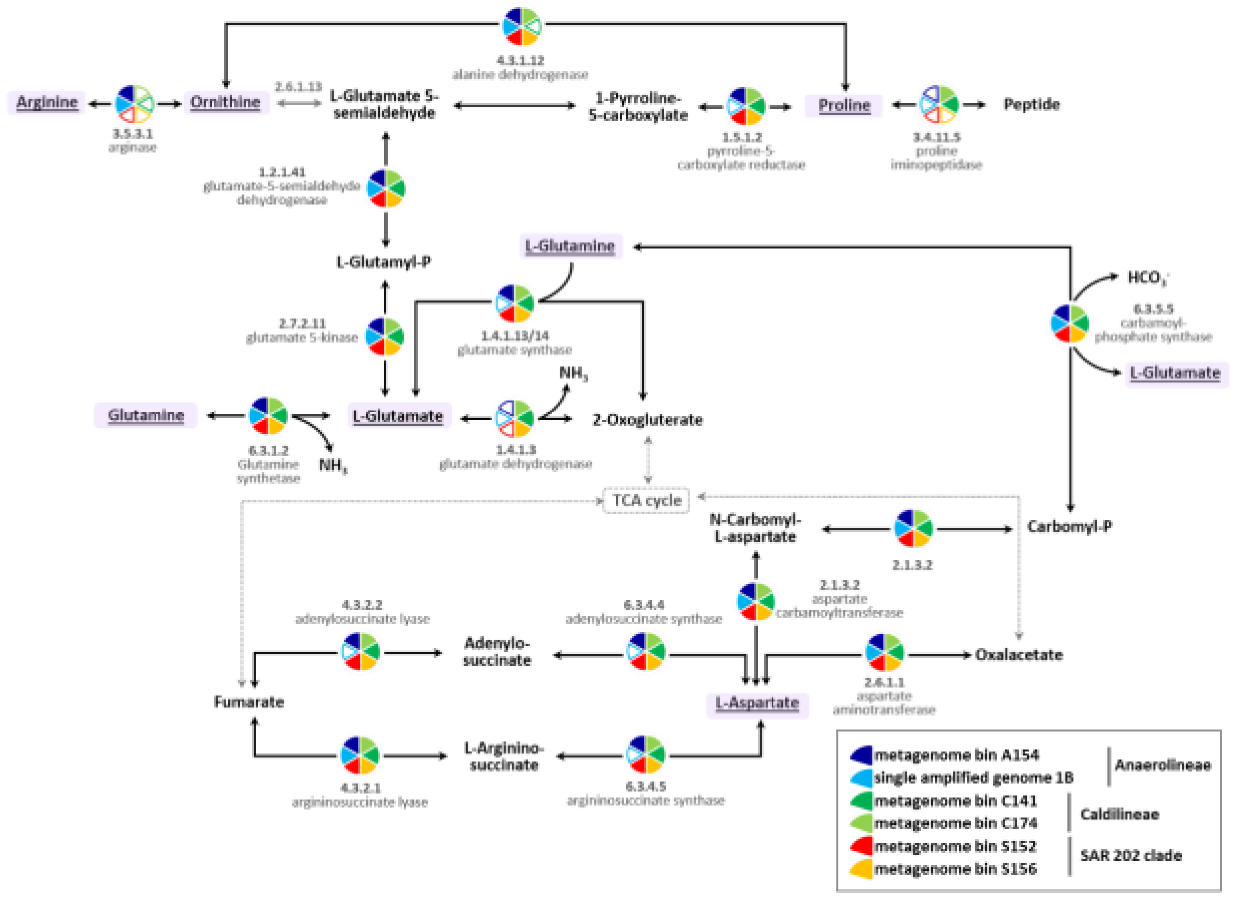

Alternatively, ornithine, which is incorporated into arginine synthesis, can be synthesized from glutamate (cyclic ornithine production, see below). The enzymes involved are annotated largely in all genomes. The lower part of ornithine synthesis (via the Arg-enzymes) is restricted to Anaerolineae and Caldilineae genomes, whereas the alternative way via N-acetylornithine seems to be a common feature. The last conversion of arginine in ornithine (arginase, EC: 3.5.1.1), making the pathway cyclic, is encoded only in Anaerolineae genomes.

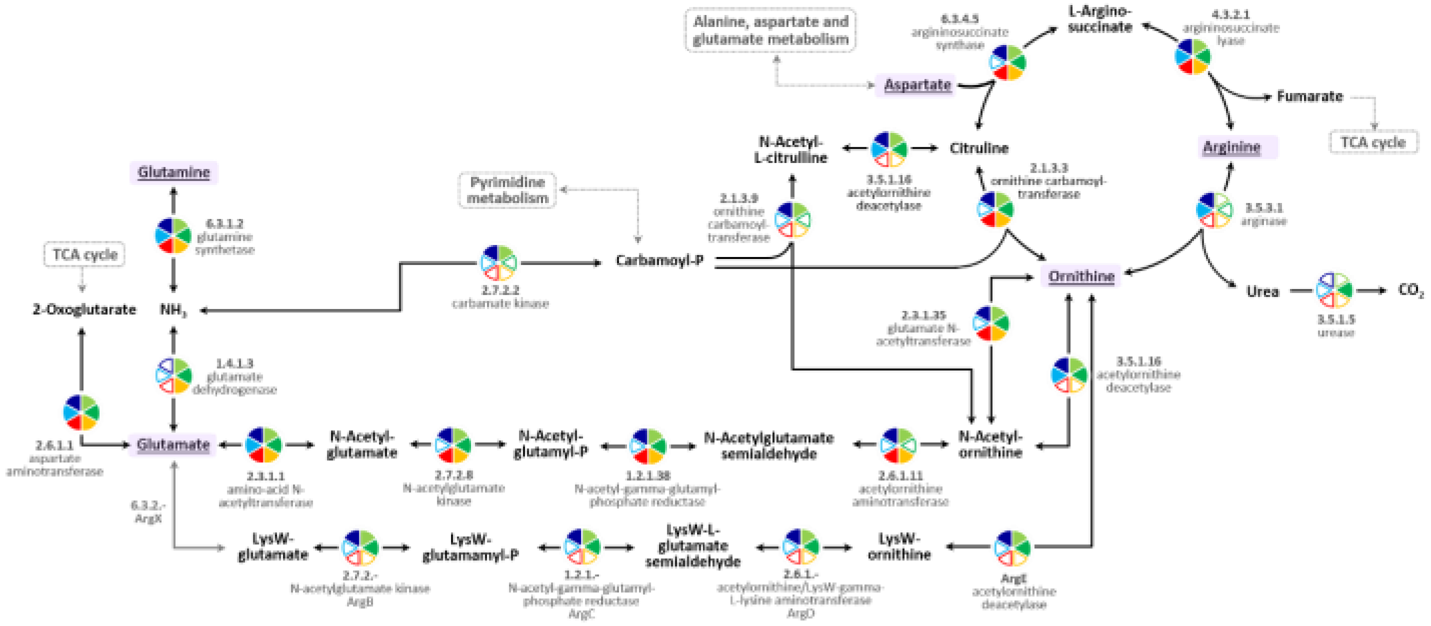

Some amino acids can be synthesized from aspartate (see below). Methionine biosynthesis from arspartate vio homoserine is encoded mainly by all six genomes, with some exception, e.g. the conversion of L-homoserine into O-succinyl-L-homoserine by the enzyme homoserine O-succinyl-transferase (EC: 2.3.1.46) is annotaded only in Anaerolineae genomes. Genomic contect relveal a phylogenetic specialization for lysine biosynthesis. While Anaerolineae and Caldilineae genomes encode most of the enzymes for the synthesis from homoisocitrate (lower part in Fig. below), SAR202 genomes encode for the synthesis from L-aspartate (middle part).

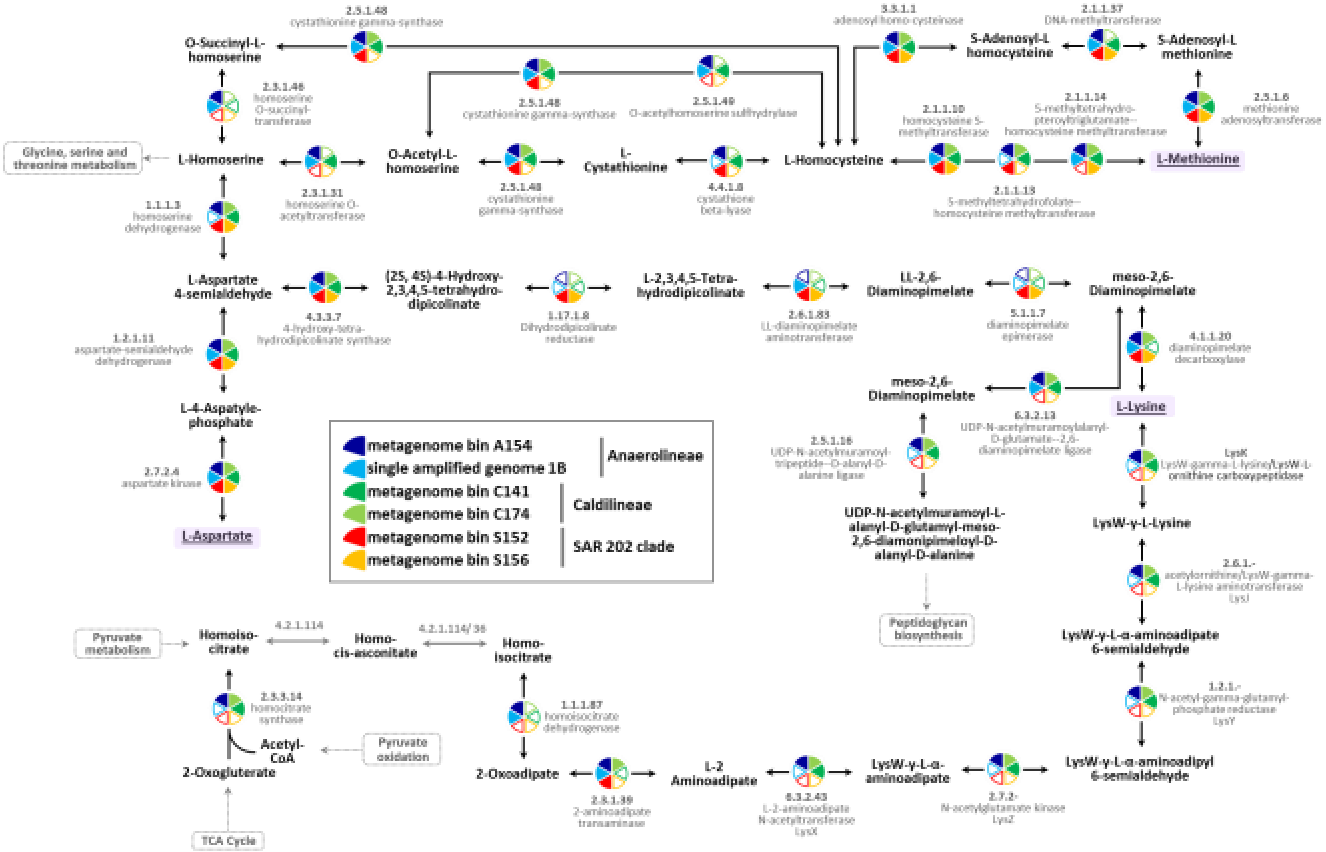

Most enzymes needed for histidine biosynthesis from PRPP (5-phosphoribosyl 1-pyrophosphate) which is provided by PPP, are encoded in nearly all genomes. The only exception is the enzyme histidinol phosphatase (EC: 3.1.3.15) which was annotated only in A154 and C141.

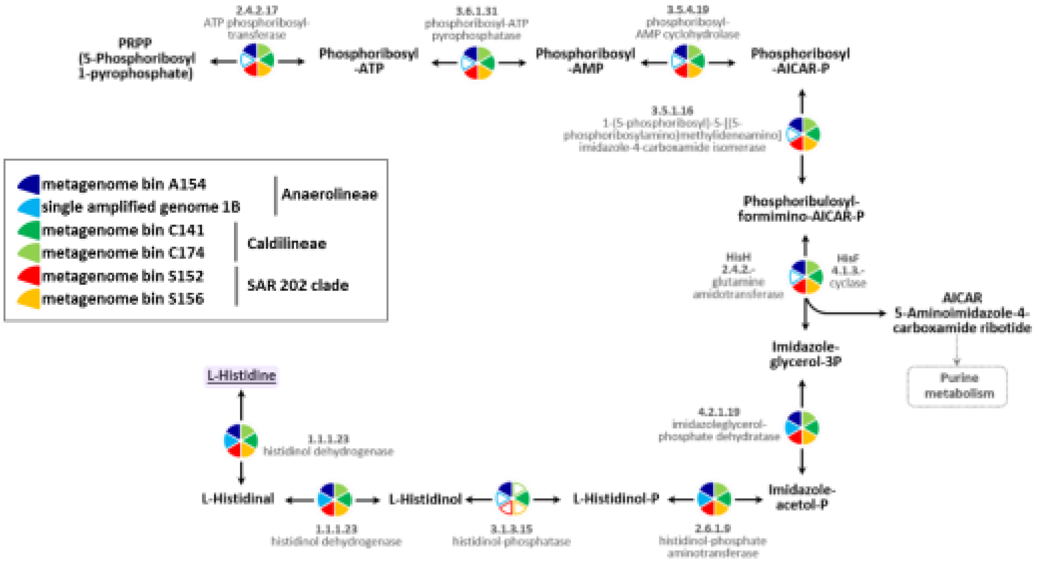

Phenylalanine and tyrosine can be synthesized from L-trypophan or from compounds taken from central metabolism (PEP from glycolysis and erythrose-4P from pentose phosphate pathway via shikimate pathway). Both ways were annotated in almost all genomes with one exception. The enzyme prehenate dehydrogenase (EC: 1.3.1.12) was encoded only in Anaerolineae and Caldilineae genomes.

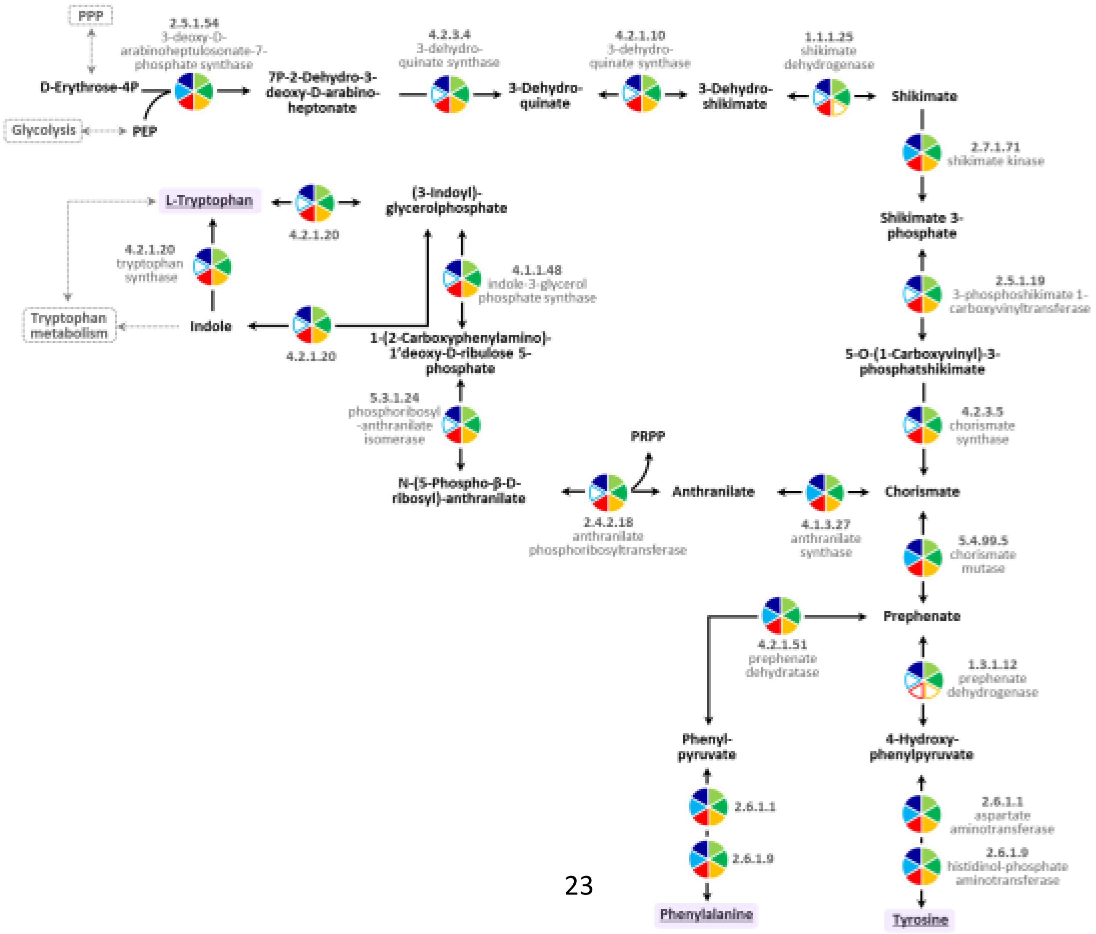

Glutathione (GSH) is a tripeptide, non-essential amino acid that could play a role as antioxidant by preventing cell damages of important cellular components caused by reactive oxygen species such as free radicals, peroxides, lipid peroxides, and heavy metals. The ABC transporter for the import of GSH is partially encoded in the SAR202 metagenome S152 and the Caldilineae metagenome C174, but absent in the other genomes. If it's not used as antioxidant it way is converted to glutamate and L-cysteinylglycine by the enzyme glutathione hydrolase (EC: 3.4.19.13). L-Cysteinylglycine can be converted to L-cysteine and glycine by the enzyme PepA [leucyl aminopeptidase, (EC: 3.4.11.1)] which is encoded in both Caldilineae genomes and in both SAR202 genomes, but not by the two Anaerolineae genomes. Using the enzyme gamm-glutamyl-transpeptidase (EC: 2.3.2.2) L-cysteinylglycine can be converted into L-amino acids.

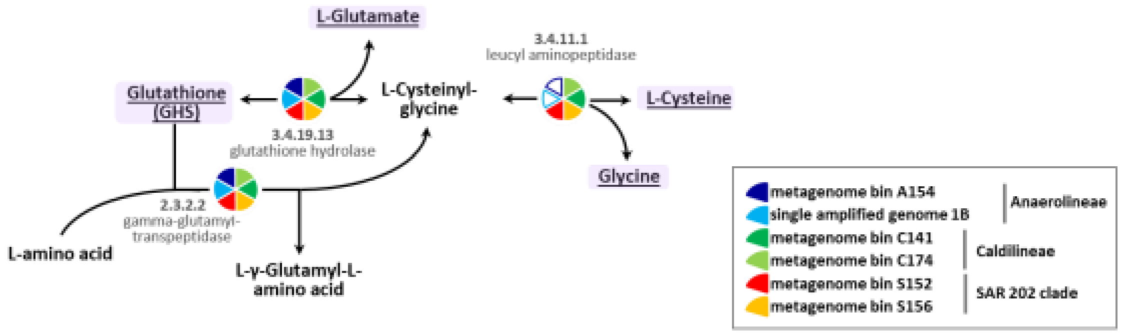

The first step of amino acid (aa) catabolism is always the removal of the amino group by amino acid oxidases, aa dehydrogenases, aa transaminases (inc. aminotransferases) and/or by a deamination reaction using dehydrogenases. All 20 aa are degenerated to an α-keto-acid intermediate (pyruvate, acetyl-CoA, acetoacetyl-CoA, α-ketogluterate, succinyl-CoA, fumarate, and oxalacetate), which could enter the TCA cycle.

Interestingly, some of these enzymes were found only in one (or two) sponge associated Chloroflexi groups so that each one seems to encode their enzyme set. A glutamine-phosphoenolpyruvate transaminase (EC: 2.6.1.64) was encoded in both Caldilineae bins, a 4-aminobutyrate aminotransferase (EC: 2.6.1.19) was found in both Anaerolineae bins, a number of dTDP-4-amino-4,6-dideoxygalactose transaminase (2.6.1.59) were annotated in both Anaerolineae and Caldilineae bins, and a glutamine--fructose-6P transaminase (EC: 2.6.1.16) in both Sar202 bins. Some transaminases were found only in one or two genomes of different groups. An alanine-glyoxylate transaminase/ serine-glyoxylate transaminase/ serine-pyruvate transaminase (EC: 2.6.1.44, 2.6.1.45, 2.6.1.5) were annotated in S152 and C174, an arginine--pyruvate transaminase (EC: 2.6.1.84) in bin A154 and a beta-alanine--pyruvate transaminase (EC: 2.6.1.18) in C141.

A non-group specific glycine/ D-amino acid oxidase (COG 0665) was found in A154, C174 and S152. Both Caldilineae genome bins encode for a D-amino acid dehydrogenase (EC: 1.4.99.-). The utilization of these compounds is highly interesting since several biological molecules (e.g. peptidoglycan, certain antibiotics) have D-amino acids. A high number of additional aminotransferases (substrate-specific and non-specific) were annotated in the genomes of all Chloroflexi groups. However, these findings suggest that all three sponge-specific Chloroflexi groups are able to use different amino acids as food (carbon and nitrogen) and energy source. In that line it needs to be mentioned that all three phylogenetic groups may also import diverse amino acids. ABC transporter for branched chain aa (LivKHMGF) was identified, as well as one for L-amino acids (AapJQMP) were annotated almost completely in all six genomes. Both SAR202 genomes partially encode for a third transporter for the import of neutral amino acids (NatBCDAE).

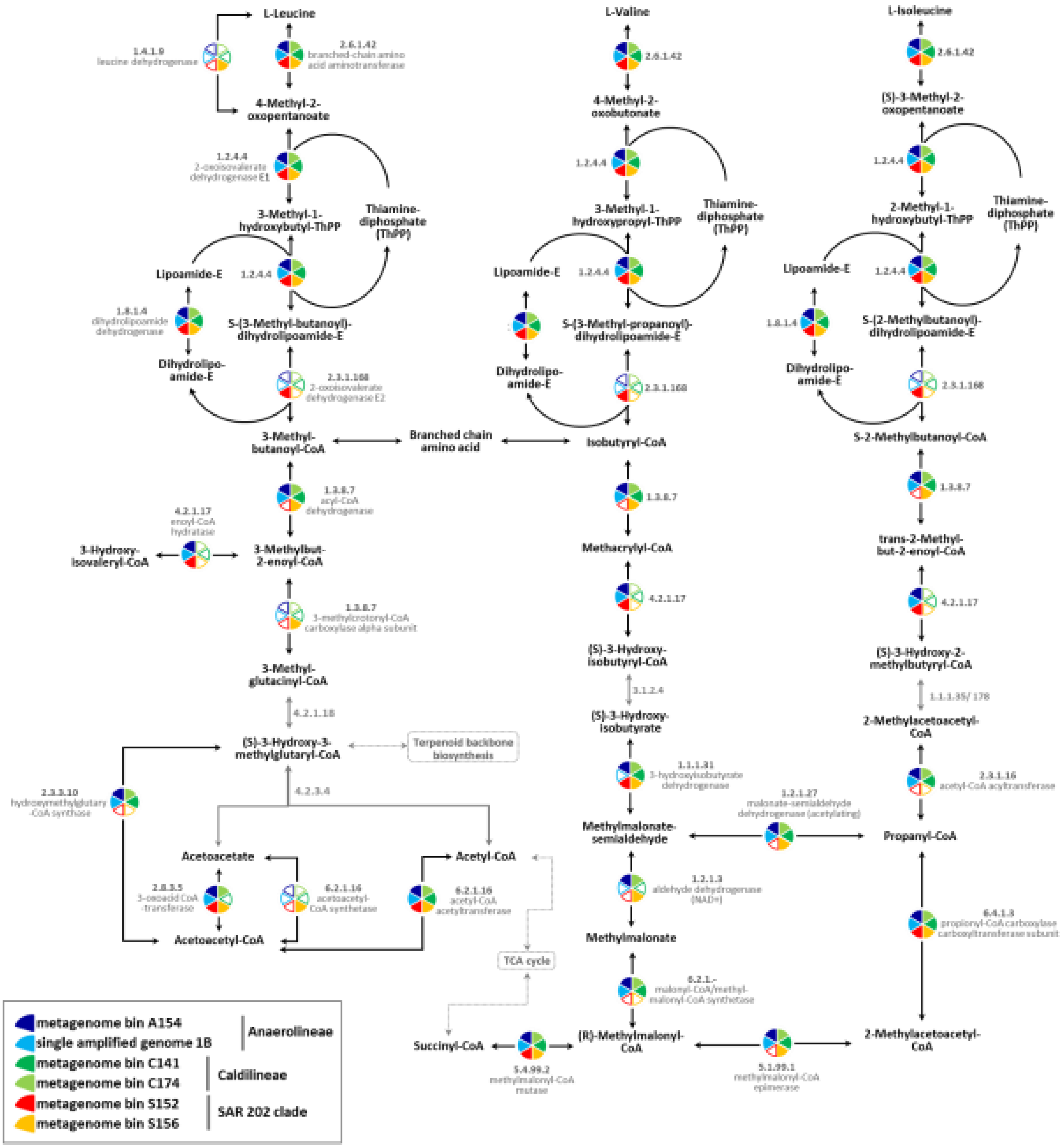

Fatty acid metabolism

All six genomes encode for the enzyme acetyl-CoA carboxylase (EC: 6.4.1.2) which converts acetyl-CoA to malonyl-CoA using ATP and hydrogen carbonate (HCO_3^-) and displays the initial reaction of fatty acid biosynthesis. The enzymes [acetyl-carrier-protein] S-malonyltransferase (EC: 2.3.1.39, fabD), 3-oxoacyl-[acyl-carrier-protein] synthase II (EC: 2.3.1.41, FabF), 3-oxoacyl-[acyl-carrier-protein reductase (EC: 2.3.1.39, FabG), enoyl-[acyl-carrier-protein] reductase [NADH] (EC: 1.3.1.9, FabI) and the long-chain acyl-CoA

synthetase (EC: 6.3.2.1) were annotated in all genomes. 3-oxoacyl-[acyl-carrier-protein] synthase-3 (EC: 2.3.1.180, FabH) and enoyl-[acyl-carrier protein] reductase III (EC: 1.3.1.104, FabL) are encoded only in Anaerolineae and Caldilineae and S152, respectively.

We could find several enzymes involved in fatty acid degradation, including long-chain acyl-CoA synthetase (EC: 6.3.2.1), acetyl-CoA C-acetyltransferase (EC: 2.3.1.9) which were annotated in all genomes. Genes encoding for 3-hydroxyacyl-CoA dehydrogenase (EC: 1.1.1.35) were annotated in all genomes except S156, for acyl-CoA dehydrogenase (EC: 1.3.8.7) in all genomes except S152, for glutaryl-CoA dehydrogenase (EC: 1.3.8.6) in all genomes except C141, genes encoding for the enzyme acetyl-CoA acyltransferase (EC: 2.3.1.16) were absent in both SAR202 genomes. The enzyme enoyl-CoA hydratase (EC: 4.2.1.17) was annotated only in both Anaerolineae genomes and in S152.

Fatty acids can be converted to aldehyde by the enzyme aldehyde dehydrogenase (NAD+) (EC: 1.2.1.3) and further to alcohol by the enzyme alcohol dehydrogenase (EC: 1.1.1.1) while the first enzyme involved was annotated only in A154, C174, and S156, the later one was found in all genomes except S152.

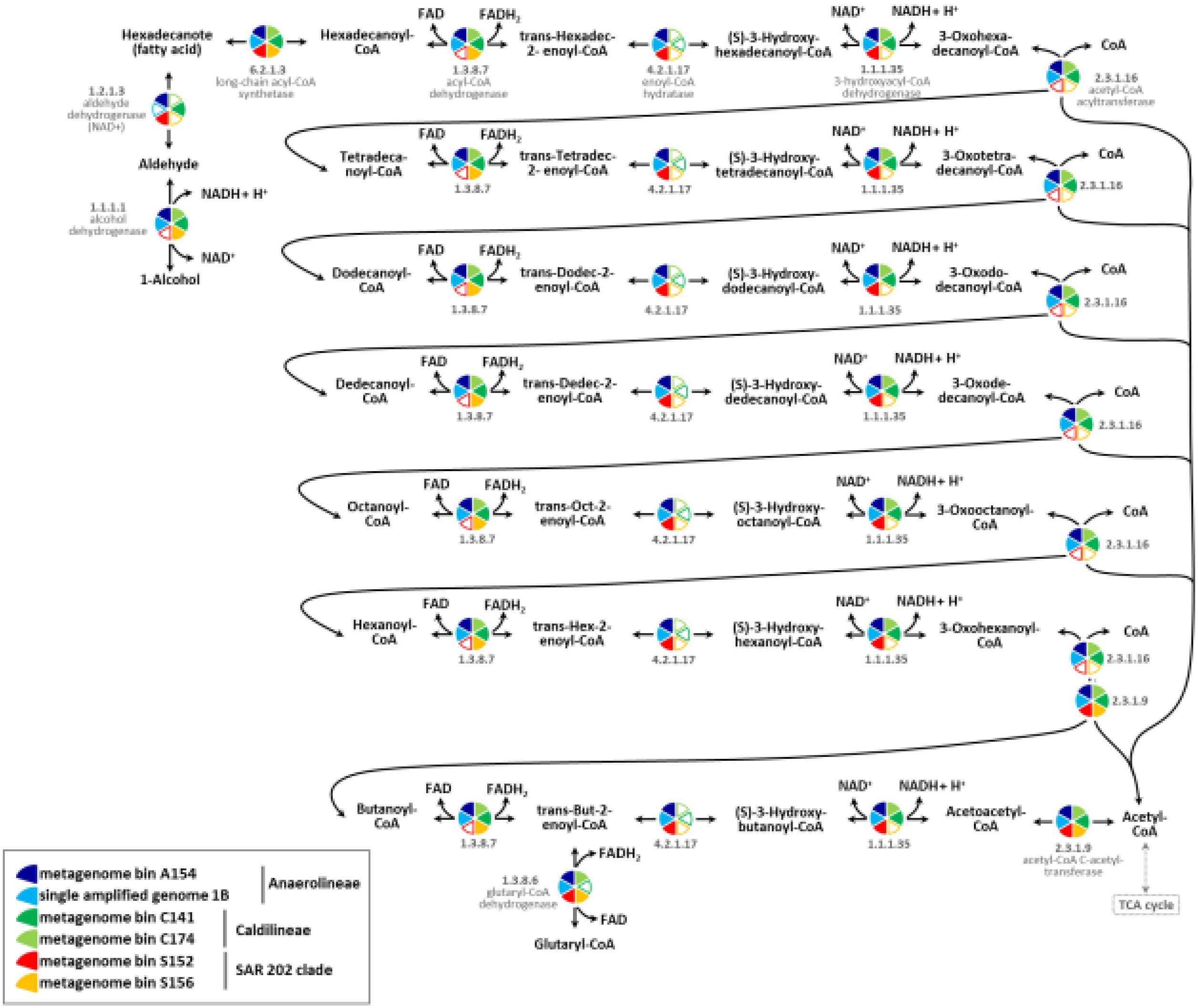

Peptidoglycan biosynthesis

Amino sugar biosynthesis provides UDP-N-acetyl glucosamine as a substrate for peptidoglycan synthesis. Interestingly, the enzymes involved are annotated in Anaerolineae and Caldilineae but not in SAR202 genomes. Therefore SAR202 might have unusual genes for peptidoglycan synthesis that they could not be annotated or they might use a different, yet undiscovered pathway.

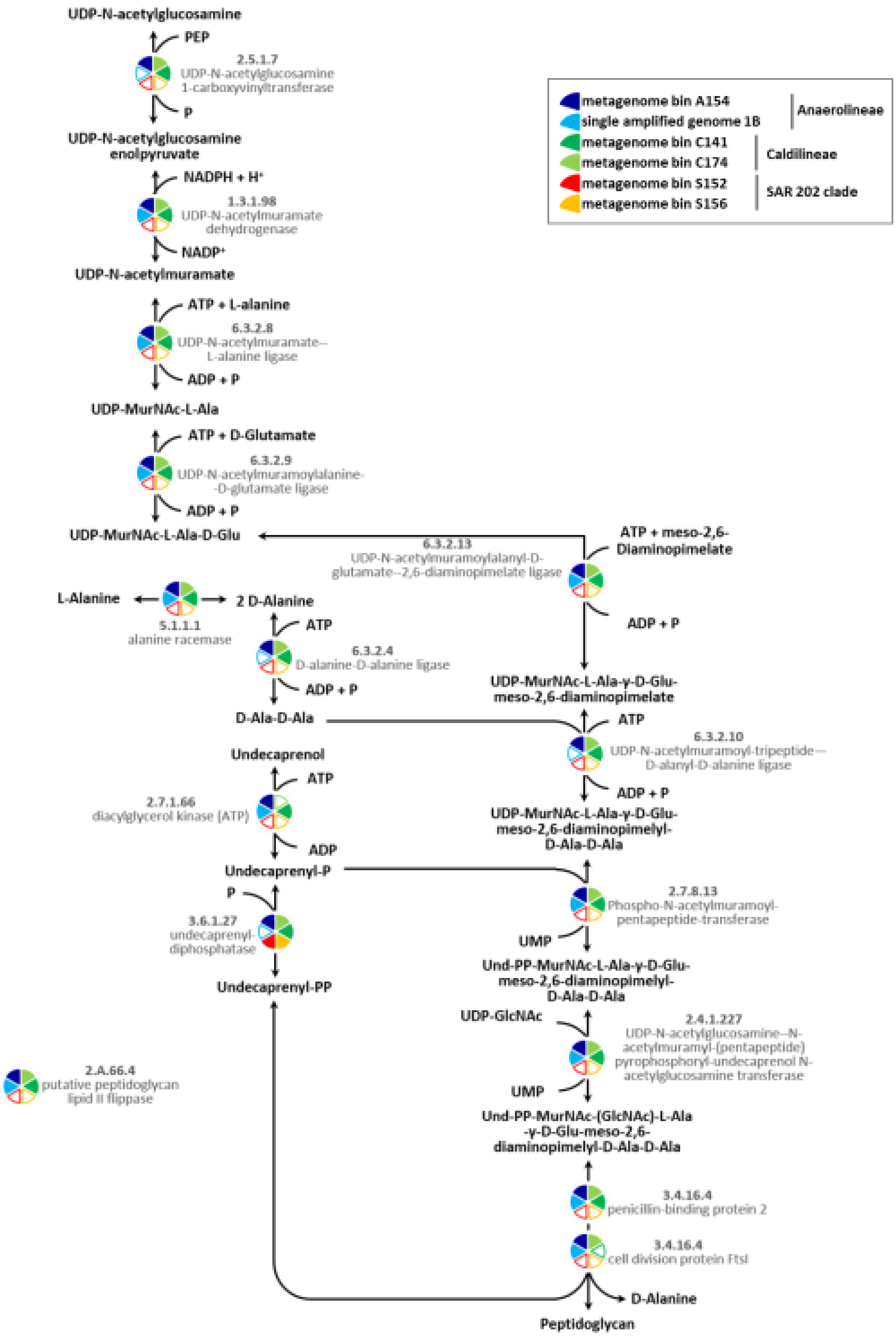

Degradation of aromatic compounds

Several enzymes involved in cleavage of aromatic rings were annotated in some genomes, however none of the degradation pathways could be found completely.

The enzyme muconate cycloisomerase (EC: 5.5.1.1) involved was annotated in both Anaerolineae genomes (one copy each) and in both Caldilineae genomes (three copies each). Additionally, the 3-oxoadipate forming enzyme 2-hydroxy-6-oxonona-2,4-dienedioate hydrolase (EC: 3.1.1.24) was found but only in bin S156. The enzymes catechol 2,3-dioxygenase (EC: 1.13.11.2 – bins C174 and S152), 4-oxalocrotonate tautomerase (EC: 5.3.2.6 – bin S152) and 2-keto-4-pentenoate hydratase (EC: 4.2.1.80 – both Sar202 bins) are involved in meta-cleavage of catechol.

Some genes involved in degradation of terephthalate were annotated in Sar202 genomes including genes coding for the enzymes 3-carboxy-cis,cis-muconate cycloisomerase (EC: 5.5.1.2 – bin S152), 4-carboxymuconolactone decarboxylase (EC: 4.1.1.44 in both Sar202 bins) and 3-oxoadipate enol-lactonase (EC: 3.1.1.24 – bin S156).

The possible degradation of compounds like *trans*-cinnamate or phenylproponate might take place since the enzymes 3-phenylpropionate/trans-cinnamate dioxygenase (EC: 1.14.12.19 – in all genomes except S152), 2,3-dihydroxyphenylpropionate 1,2-dioxygenase (EC: 1.13.11.16 - bin S156) and 2-hydroxy-6-oxonona-2,4-dienedioate hydrolase (EC: 3.7.1.14 – bin S156) could be annotated.

The enzymes cyclohexanone monooxygenase (EC: 1.14.13.22 – SAG1A and bin S152), gluconolactonase (EC: 3.1.1.17 – all genomes except S156) and NADP-dependent aldehyde dehydrogenase (EC:1.2.1.4 – bins A154 and C174) are involved in ring cleavage of cyclohexanol and the formation of adipyl-CoA via Baeyer-Villinger oxidation.

There were some genes involved in anaerobic degradation of toluene into succinyl-CoA and benzoyl-CoA. The enzymes benzylsuccinate CoA-transferase BbsF subunit (EC:2.8.3.15 – boths Sar202 bins), E-phenylitaconyl-CoA hydratase (EC:4.2.1.-, bin S156) and 2-[hydroxy(phenyl)methyl]-succinyl-CoA dehydrogenase BbsD subunit (EC:1.1.1.35 – bin C174) involved were found in some genomes. Further there is a ring cleavage via beta-oxidation in pimeloyl-CoA: the enzymes 2-hydroxycyclohexanecarboxyl-CoA dehydrogenase (EC:1.1.1.-, -both Sar202 bins) and 2-ketocyclohexanecarboxyl-CoA hydrolase (EC:3.1.2.-, bin S152) were annotated.

Literature

1. Orita, I. *et al.* The ribulose monophosphate pathway substitutes for the missing pentose phosphate pathway in the archaeon Thermococcus kodakaraensis. *J. Bacteriol.* **188,** 4698–4704 (2006).
2. Kamke, J. *et al.* Single-cell genomics reveals complex carbohydrate degradation patterns in poribacterial symbionts of marine sponges. *ISME J.* **7,** 2287–2300 (2013).
3. Pawlik, J. R. The Chemical Ecology of Sponges on Caribbean Reefs: Natural Products Shape Natural Systems. *Bioscience* **61,** 888–898 (2011).
4. Hug, L. A. *et al.* Community genomic analyses constrain the distribution of metabolic traits across the Chloroflexi phylum and indicate roles in sediment carbon cycling. *Microbiome* **1,** 1–17 (2013).
5. Landry, Z., Swa, B. K., Herndl, G. J., Stepanauskas, R. & Giovannoni, S. J. SAR202 genomes from the dark ocean predict pathways for the oxidation of recalcitrant dissolved organic matter. *MBio* **8,** 1–19 (2017).
6. Spaink, H. P., Wijfjes, A. H. M. & Lugtenberg, B. J. J. Rhizobium nodI and NodJ proteins play a role in the efficiency of secretion of lipochitin oligosaccharides. *J. Bacteriol.* **177,** 6276–6281 (1995).
7. Horn, H. *et al.* An Enrichment of CRISPR and other defense-related features in marine sponge-associated microbial metagenomes. *Front. Microbiol.* **7,** (2016).
8. Nong, G., Rice, J. D., Chow, V. & Preston, J. F. Aldouronate utilization in Paenibacillus sp. strain JDR-2: Physiological and enzymatic evidence for coupling of extracellular depolymerization and intracellular metabolism. *Appl. Environ. Microbiol.* **75,** 4410–4418 (2009).
9. Michell, R. H. Inositol and its derivatives: Their evolution and functions. *Adv. Enzyme Regul.* **51,** 84–90 (2011).
10. Reynolds, T. B. Strategies for acquiring the phospholipid metabolite inositol in pathogenic bacteria, fungi and protozoa: making it and taking it. *Microbiology* **155,** 1386–1396 (2009).
11. Müller, W. E. *et al.* Role of the aggregation factor in the regulation of phosphoinositide metabolism in sponges. Possible consequences on calcium efflux and on mitogenesis. *J. Biol. Chem.* **262,** 9850–9858 (1987).
