# Supplementary material for: Marine Sponges as Chloroflexi Hot Spots: Genomic Insights and High-Resolution Visualization of an Abundant and Diverse Symbiotic Clade
Source: mSystems. 2018 Dec 26;3(6):e00150-18. doi: 10.1128/mSystems.00150-18 (PMC6306507; doi:10.1128/mSystems.00150-18)
Supplement: TABLE S3 [file sys006182305st3.docx]

Table S3: Absolute abundance of Carbohydrate active enzymes detected in genomes

|  | **Anaerolineae** | |  | **Caldilineae** | |  | **SAR202** | |
| --- | --- | --- | --- | --- | --- | --- | --- | --- |
|  | **SAG 1B** | **A154** |  | **C141** | **C174** |  | **S152** | **S156** |
| **AA2** |  |  |  | 2 |  |  |  | 3 |
| **AA3** | 2 | 5 |  | 1 | 3 |  | 12 | 3 |
| **AA4** |  |  |  | 1 |  |  | 2 | 1 |
| **AA7** |  | 1 |  | 1 | 3 |  | 1 |  |
| **sum AA** | **2** | **6** |  | **5** | **6** |  | **15** | **7** |
| **CBM13** |  |  |  |  |  |  |  | 1 |
| **CBM20** |  | 1 |  |  |  |  |  |  |
| **CBM32** |  |  |  |  | 4 |  |  |  |
| **CBM34** |  | 1 |  |  |  |  |  |  |
| **CBM37** | 2 | 1 |  |  | 13 |  | 1 | 1 |
| **CBM44** | 6 | 1 |  |  |  |  | 2 |  |
| **CBM48** | 1 | 3 |  | 1 |  |  |  |  |
| **CBM50** | 10 | 30 |  | 47 | 42 |  |  |  |
| **CMB51** |  |  |  |  | 1 |  |  |  |
| **CBM66** | 4 | 1 |  | 1 | 4 |  | 1 |  |
| **CBM67** |  |  |  |  | 2 |  |  |  |
| **sum CBM** | **23** | **38** |  | **49** | **66** |  | **4** | **2** |
| **CE1** | 2 | 7 |  | 2 | 3 |  | 6 | 9 |
| **CE3** |  |  |  |  |  |  | 1 |  |
| **CE4** |  | 1 |  | 4 | 5 |  | 5 | 1 |
| **CE6** | 1 |  |  |  |  |  |  |  |
| **CE7** | 3 | 3 |  | 1 | 6 |  | 5 | 5 |
| **CE9** | 1 | 1 |  | 1 | 1 |  |  |  |
| **CE10** | 6 | 5 |  | 5 | 3 |  | 5 | 7 |
| **CE12** | 2 |  |  |  |  |  |  |  |
| **CE14** | 3 | 4 |  | 4 | 4 |  | 5 | 2 |
| **CE15** |  |  |  | 1 | 6 |  |  |  |
| **sum CE** | **18** | **21** |  | **18** | **28** |  | **27** | **24** |
| **GH2** | 1 | 1 |  | 5 | 2 |  |  |  |
| **GH3** | 2 | 3 |  | 2 | 1 |  |  |  |
| **GH4** | 2 | 4 |  | 6 | 4 |  |  |  |
| **GH5** | 2 | 1 |  | 1 | 2 |  |  |  |
| **GH12** |  |  |  | 1 |  |  |  |  |
| **GH13** | 2 | 4 |  | 3 | 2 |  |  |  |
| **GH15** |  |  |  |  |  |  | 1 | 1 |
| **GH16** |  |  |  | 1 | 1 |  |  |  |
| **GH18** | 3 | 1 |  |  | 1 |  |  |  |
| **GH20** | 2 | 1 |  |  |  |  |  |  |
| **GH23** | 2 | 5 |  |  | 3 |  |  | 1 |
|  | **Anaerolineae** | |  | **Caldilineae** | |  | **SAR202** | |
|  | **SAG 1B** | **A154** |  | **C141** | **C174** |  | **S152** | **S156** |
| **GH29** | 5 | 7 |  | 4 | 3 |  |  |  |
| **GH31** |  |  |  | 1 |  |  |  |  |
| **GH32** | 5 | 1 |  | 3 | 12 |  | 1 |  |
| **GH33** |  | 1 |  | 6 | 14 |  |  |  |
| **GH36** |  |  |  |  | 4 |  |  |  |
| **GH38** | 1 | 1 |  |  |  |  |  |  |
| **GH39** | 6 | 7 |  | 6 | 8 |  |  |  |
| **GH42** | 1 |  |  |  | 2 |  |  |  |
| **GH50** |  |  |  | 1 |  |  |  |  |
| **GH51** | 1 | 2 |  | 1 |  |  |  |  |
| **GH63** |  |  |  | 1 | 1 |  |  |  |
| **GH74** | 3 | 12 |  | 3 | 14 |  | 5 | 10 |
| **GH76** |  | 1 |  |  |  |  |  |  |
| **GH77** | 1 | 1 |  |  |  |  |  |  |
| **GH78** |  |  |  |  | 2 |  |  |  |
| **GH88** |  |  |  | 1 | 1 |  |  |  |
| **GH93** | 1 | 1 |  |  | 8 |  |  |  |
| **GH96** |  | 1 |  |  |  |  |  |  |
| **GH105** | 1 | 1 |  |  | 2 |  | 2 |  |
| **GH106** |  |  |  |  | 3 |  |  |  |
| **GH109** | 15 | 28 |  | 43 | 107 |  | 8 | 13 |
| **GH113** |  |  |  |  |  |  |  |  |
| **GH116** |  | 1 |  | 2 | 1 |  |  |  |
| **GH117** |  | 8 |  |  | 4 |  |  |  |
| **GH127** | 1 | 3 |  | 2 | 1 |  |  |  |
| **GH130** |  |  |  | 1 |  |  |  |  |
| **GH139** |  |  |  | 1 |  |  |  |  |
| **GH140** |  |  |  | 1 |  |  |  |  |
| **GH145** |  |  |  | 1 |  |  |  |  |
| **sum GH** | **57** | **96** |  | **94** | **203** |  | **17** | **25** |
| **GT1** |  |  |  |  | 1 |  |  |  |
| **GT2** | 12 | 18 |  | 6 | 16 |  | 9 | 4 |
| **GT3** |  |  |  |  | 1 |  |  |  |
| **GT4** | 15 | 27 |  | 16 | 25 |  | 2 | 6 |
| **GT5** |  | 1 |  |  |  |  |  |  |
| **GT8** | 1 |  |  |  |  |  |  |  |
| **GT9** |  | 3 |  |  |  |  |  |  |
| **GT20** |  |  |  |  |  |  |  | 1 |
| **GT26** | 2 | 1 |  | 2 | 1 |  |  |  |
| **GT27** |  |  |  | 1 | 1 |  |  |  |
| **GT28** | 3 | 2 |  | 2 | 2 |  |  |  |
|  | **Anaerolineae** | |  | **Caldilineae** | |  | **SAR202** | |
|  | **SAG 1B** | **A154** |  | **C141** | **C174** |  | **S152** | **S156** |
| **GT35** |  | 2 |  | 1 | 2 |  |  |  |
| **GT39** | 5 | 4 |  |  | 6 |  | 2 | 1 |
| **GT47** |  |  |  |  |  |  |  |  |
| **GT51** |  | 3 |  | 1 | 3 |  |  |  |
| **GT83** | 16 | 28 |  | 7 | 28 |  | 4 | 2 |
| **GT94** |  |  |  |  | 1 |  |  |  |
| **sum GT** | **54** | **89** |  | **36** | **87** |  | **17** | **14** |
| **PL9** |  | 1 |  |  |  |  |  |  |
| **PL12** |  |  |  | 1 |  |  |  |  |
| **PL15** |  | 1 |  |  |  |  |  |  |
| **PL17** |  |  |  |  | 1 |  |  |  |
| **PL22** | 3 | 2 |  | 2 |  |  |  | 1 |
| **sum PL** | **3** | **4** |  | **3** | **1** |  | **0** | **1** |

Table S3, continued, AA: Auxiliary Activities, CBM: Carbohydrate-Binding Modules, CE: Carbohydrate Esterases, GH: Glycoside Hydrolases, GT: GlycosylTransferases, PL: Polysaccharide Lyases.
