# Supplementary material for: Marine Sponges as Chloroflexi Hot Spots: Genomic Insights and High-Resolution Visualization of an Abundant and Diverse Symbiotic Clade
Source: mSystems. 2018 Dec 26;3(6):e00150-18. doi: 10.1128/mSystems.00150-18 (PMC6306507; doi:10.1128/mSystems.00150-18)
Supplement: TABLE S4 [file sys006182305st4.docx]

Table S4: Absolute abundance of enzymes possibly involved in recalcitrant DOM degradation

| **Annotated function** | **Anaerolineae** | |  | **Caldilineae** | |  | **SAR202** | |
| --- | --- | --- | --- | --- | --- | --- | --- | --- |
|  | **SAG 1B** | **A154** |  | **C141** | **C174** |  | **S152** | **S156** |
| Acyl-CoA-transferase CaiB and other family III transferases | 1 | 2 |  |  | 3 |  | 39 | 13 |
| Flavin-dependent oxidoreductase, luciferase family (includes alkanesulfonate monooxygenase SsuD and methylene tetrahydromethanopterin reductase) | 2 | 1 |  | 2 | 8 |  | 36 | 17 |
| Short chain alcohol dehydrogenase | 7 | 4 |  | 2 | 3 |  | 15 | 7 |
| 2-oxoglutarate:ferrodoxin oxidoreductase |  | 1 |  | 1 | 1 |  | 1 | 2 |
| Carbon-monoxide dehydrogenase subunits | 3 | 3 |  | 8 | 10 |  | 3 | 17 |
| CO or xanthine dehydrogenase | 1 | 4 |  | 2 | 4 |  | 2 | 4 |
| Choline dehydrogenase | 2 | 5 |  |  | 1 |  | 6 | 3 |
| Sarcosine oxidase | 4 | 3 |  | 2 | 2 |  | 1 | 1 |
| serine hydroxymethyltransferase | 2 | 1 |  | 1 | 1 |  | 1 | 1 |
| formaldehyde dehydrogenase |  |  |  | 1 | 1 |  | 1 |  |
| formate dehydrogenase | 2 | 2 |  | 6 | 4 |  | 6 | 3 |
