# Supplementary material for: Marine Sponges as Chloroflexi Hot Spots: Genomic Insights and High-Resolution Visualization of an Abundant and Diverse Symbiotic Clade
Source: mSystems. 2018 Dec 26;3(6):e00150-18. doi: 10.1128/mSystems.00150-18 (PMC6306507; doi:10.1128/mSystems.00150-18)
Supplement: TABLE S5 [file sys006182305st5.docx]

Table S5: Absolute number (and relative values) of A: eukaryotic like proteins (ELPs) and B: secondary metabolite gene cluster identified in analyzed genomes.

|  | **Anaerolineae** | |  | **Caldilineae** | |  | **SAR202** | |
| --- | --- | --- | --- | --- | --- | --- | --- | --- |
| **A: eukaryotic like proteins** | **SAG 1B** | **A 154** |  | **C 141** | **C 174** |  | **S 152** | **S 156** |
| **Ankyrin repeat domain containing protein (ANK)** | 1  (0.0373) | 2  (0.0605) |  | 9  (0.2125) | 1  (0.0178) |  | 5  (0.0930) | 2  (0.0588) |
| **Leucin-rich repeat domain containing protein (LRR)** | 0 | 0 |  | 4  (0.0944) | 12  (0.2142) |  | 10  (0.1859) | 3  (0.0882) |
| **Tetratricopeptide repeat-containing protein (TPR)** | 9  (0.3359) | 17  (0.5141) |  | 9  (0.2125) | 16  (0.2857) |  | 17  (0.3161) | 3  (0.0882) |
| **WD-40 repeat-containing protein** | 0 | 9  (0.2721) |  | 6  (0.1416) | 4  (0.0714) |  | 1  (0.0186) | 2  (0.0588) |
| **B: Secondary metabolite gene cluster** | | | | | | | | |
| **Type 1 PKS** | 0 | 0 |  | 2  (0.0472) | 0 |  | 3  (0.0558) | 1  (0.0294) |
| **other KS** | 0 | 0 |  | 0 | 0 |  | 0 | 0 |
| **Terpene** | 0 | 0 |  | 0 | 1  (0.0179) |  | 4  (0.0744) | 2  (0.0588) |
| **others** | 0 | 0 |  | 0 | 0 |  | 1  (0.0186) | 1  (0.0294) |
